# Brazilian Mining Dam Collapse: Molecular Networking–Guided Metabolomics Reveals Species-Specific Plant Detox

**DOI:** 10.1021/acsomega.5c11096

**Published:** 2026-01-06

**Authors:** Marília Elias Gallon, Eduardo Afonso Silva-Junior, Amanda Roberta Corrado, Maria Das Graças Lins Brandão, Maria Cristina Teixeira Braga Messias, Alberto José Cavalheiro, Norberto Peporine Lopes, Alan Cesar Pilon

**Affiliations:** † University of Sao Paulo, Faculty of Pharmaceutical Sciences of Ribeirao Preto, Biomolecular Sciences, Prof. Dr. Zeferino Vaz Avenue, Ribeirao Preto 14040-903, Brazil; ‡ 67826 Federal University of Mato Grosso, Unidade II, N 6390 Valdon Varjão Avenue, Barra Do Garças 78600-000, Brazil; § 153998 Federal University of Minas Gerais, Faculty of Pharmacy, Centro Especializado Em Plantas Aromáticas, Medicinais E Tóxicas, Museu de História Natural e Jardim Botânico de Belo Horizonte, 1035 Gustavo da Silveira Street, Belo Horizonte 31270-901, Brazil; ∥ 28115 Federal University of Ouro Preto, Institute of Exact and Biological Sciences, Biodiversidade, Evolução e Meio Ambiente, 786 Quatro Street, Ouro Preto 35402-136, Brazil; ⊥ 153997 São Paulo State University, Institute of Chemistry, Biochemistry and Organic Chemistry, 55 Francisco Degni Street, Araraquara 14800-060, Brazil

## Abstract

In November 2015, a catastrophic environmental disaster struck the state of Minas Gerais, Brazil, which was caused by the collapse of the Fundão dam. Over 40 million m^3^ of metal-rich mining waste, containing iron, arsenic, mercury, cadmium, and manganese, contaminated more than 650 km of the Doce River basin, causing severe degradation of terrestrial and aquatic ecosystems. While immediate effects included widespread destruction of local habitats, including indigenous settlements, some endemic plant species exhibited remarkable resilience, adapting their metabolism to tolerate extreme exposure to toxic mining waste. Using an untargeted liquid chromatography-tandem mass spectrometry (LC-MS^n^) metabolomics workflow integrated with multivariate analysis and molecular networking, we profiled metabolic changes in two medicinal plants, *Vernonanthura polyanthes* (Asteraceae) and *Piper aduncum* (Piperaceae). Our results showed that exposure to toxic mining waste elicited species-specific responses, with plants triggering biosynthetic pathways that enhanced production of peptides, especially glutathione and lysine-acetylated derivatives, for *V. polyanthes*, and phenylpropanoids, especially *O*-methylated *C*-glycosylated flavonoids, for *P. aduncum*. These shifts are consistent with defense mechanisms involving metal chelation and redox buffering, whereby glutathione-based peptides and flavonoids mitigate metal toxicity and oxidative stress in plant tissues. Overall, these findings shed light on the mechanisms of plant response under extreme environmental disturbance, providing insights into how metabolic adaptations contribute to ecological stabilization and plant recovery.

## Graphical abstract

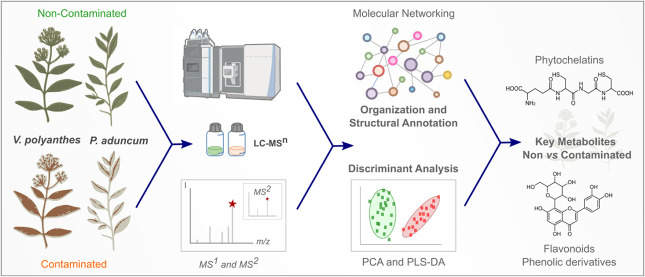

## Introduction

The origins of the mining industry in Brazil date back to the 18th century, when the country experienced an early and intense gold rush period.^1,2^ Over the years, mining exploitation scenario has expanded and, nowadays, Brazil is globally considered one of the largest iron ore producers.^3–5^ To manage waste generated during ore processing, mining companies rely on tailings dams designed to store and contain waste materials (e.g., water, sediments, metal fragments, and other toxic byproducts) and prevent toxic waste from contaminating the environment.^6^ As of September 2025, Brazil had 918 registered mining tailing dams, with 327 located in the state of Minas Gerais.^7^ Despite the sector’s economic relevance, mining profoundly alters ecosystems, causing landscape degradation, loss of native biodiversity, soil contamination, and disturbances to aquatic habitats.^2,3,8,9^

In recent years, catastrophic tailing dam failures have become more frequent.^10^ On November 5, 2015, the Fundão dam (operated by Samarco Mineração SA) collapsed near the city of Mariana in the state of Minas Gerais, releasing over 40 million m^3^ of mining waste containing iron (Fe), arsenic (As), mercury (Hg), manganese (Mn), and cadmium (Cd) into the Doce River basin.^11–13^ The toxic mining waste traveled more than 650 km across the Doce River basin, ultimately reaching the Atlantic Ocean (Figure 1a and b). Consequently, the environmental disaster dramatically impacted the local flora, including relevant plant species, and altered surrounding ecosystems causing persistent ecological damage to both terrestrial and aquatic habitats,^11,14,15^

**Figure 1:**
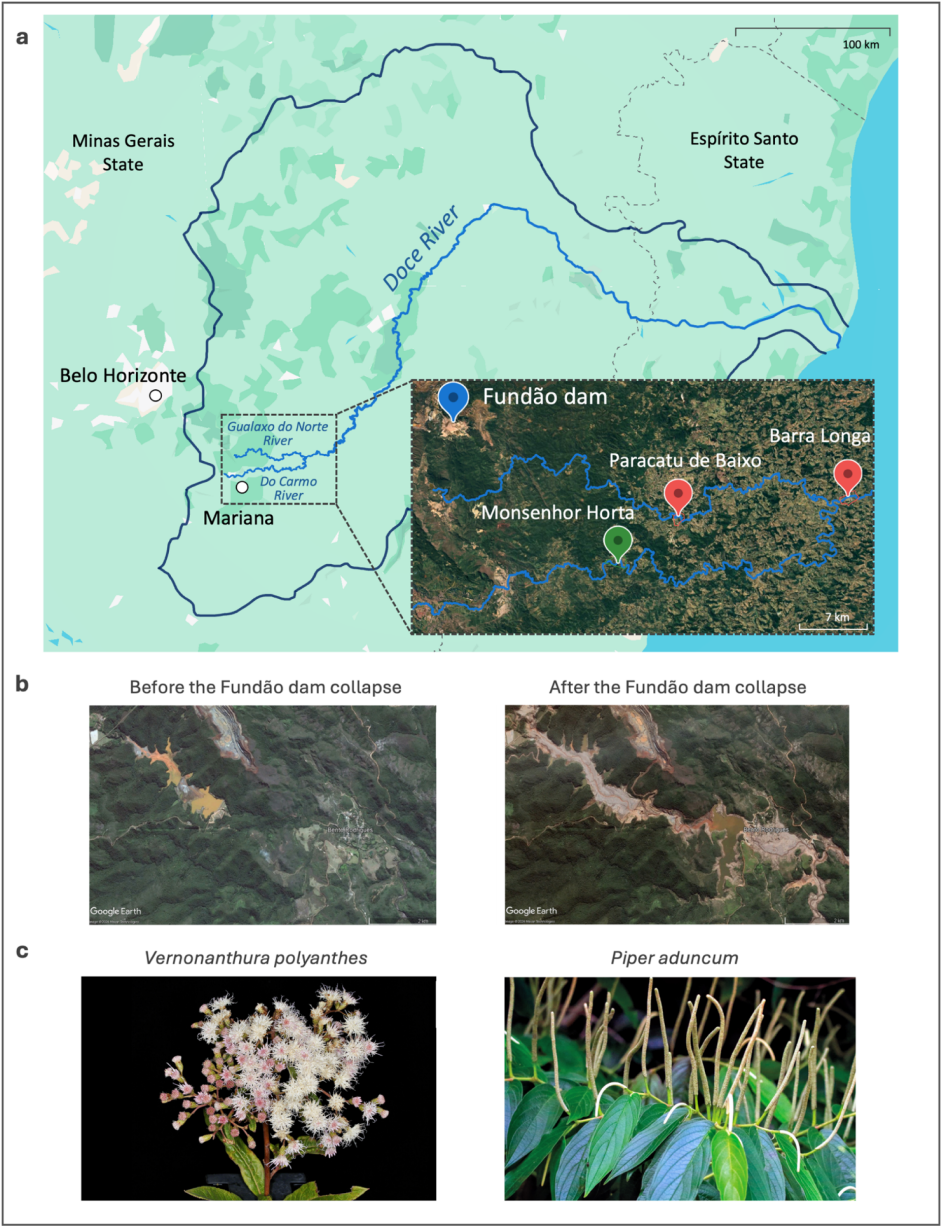
Geographical location of the Fundão dam and photographs of the studied plant species. **a**, Map illustrating the trajectory of mining waste following the Fundão dam collapse across the Doce River basin (outlined in dark blue) and the locations of plant collections in affected (red pins) and unaffected (green pin) areas. **b**, Satellite photographs of the Fundão Dam and the city of Bento Rodrigues in July 2015 (before the dam collapse) and June 2016 (after the dam collapse), sourced from Google Earth Pro. **c**, Photographs of *Vernonanthura polyanthes* and *Piper aduncum* individuals, two traditional Brazilian medicinal plants. Photograph courtesy of Mauricio Mercadante and Marcelo Kuhlmann.^21^ Copyright 2025.

The Doce River basin spans Atlantic Forest biome, particularly in its central and lower sections, while savanna-like zones occur in transitional areas of the upper basin.^16–18^ The region hosts a wide variety of plant species belonging to more than 30 families, including Fabaceae, Myrtaceae, Lauraceae, Asteraceae, Malvaceae, Piperaceae, Bromeliaceae, and Orchidaceae.^19–22^ Considering the environmental footprint of the Fundão dam collapse and the regional importance of medicinal plants for the local communities, we focused our study on two widely used plant species from distinct families: *Vernonanthura polyanthes* (Spreng.) Vega & Dematteis (Asteraceae), known as “*assa-peixe*”, and *Piper aduncum* L. (Piperaceae) locally referred as *jaborandi-do-mato* ^21,23,24–26^ (Figure 1c).

*Vernonanthura polyanthes* is a member of the Asteraceae family and is native to Brazil, where it is predominantly found in the savanna biome. The species was originally named *Vernonia polyanthes* (Spreng.) Less.; however, it was reclassified into the genus *Vernonanthura* based on the taxonomic revision proposed by Robinson (1999).^27–30^ *Vernonanthura polyanthes* is traditionally used to treat bronchitis, pneumonia, gastrointestinal disorders, kidney diseases, and malaria.^31^ Studies revealed that *V. polyanthes* extracts exhibited cytotoxic effects and anti-inflammatory activity associated with the occurrence of flavonoids, such as rutin, luteolin and apigenin.^32,33^ *Piper aduncum* is a flowering plant of the family Piperaceae and widely distributed across Central and South America.^31,34,35^ The species is used in folk medicine to treat diarrhea, nausea, ulcers, and urinary infections, and as antihemorrhagic agent.^36,37^ Studies demonstrated that the essential oil of *P. aduncum* displayed multiple biological activities, notably insecticidal, molluscicidal, acaricidal, antiparasitic, and antibacterial activities.^38,39^

Over the past decade, metabolomics has become central to environmental chemistry and ecology, revealing organismal biochemical responses to ecosystem changes.^40–43^ Recent reviews have emphasized how metabolomics enables a comprehensive understanding of plant responses to abiotic stressors, including heavy metal contamination, through shifts in primary and specialized metabolite pathways.^44,45^ LC-MS-based metabolomic approaches enable system-level coverage of primary and specialized metabolism and have been increasingly implemented in elucidating plant responses to abiotic stressors, including metal exposure.^44,45^

Plants exposed to mining waste containing Fe, Mn, Cd, Hg, As, and other metals commonly deploy two coordinated detoxification pathways: a thiol-based axis centered on glutathione derivatives (including phytochelatins) and a remodeling of phenolic metabolism that enhances redox buffering and metal complexation.^46–49^ Along the thiol axis, multiple studies have reported dose-dependent induction of phytochelatins and the formation of phytochelatin–metal complexes, most prominently under Cd and Hg exposure, accompanied by shifts in glutathione pools (e.g., *Asparagus acutifolius* under Hg and *Silene vulgaris* under Cd), although this response is not universal across taxa or ecotypes (e.g., *Dianthus carthusianorum* preferentially accumulates malate and citrate under zinc (Zn), lead (Pb), and Cd exposure).^50–54^ In parallel, increases in phenolic compounds including hydroxycinnamic acid derivatives, flavonoids, and procyanidins, have been widely observed in metalliferous contexts, often accompanied by elevated peroxidase, superoxide dismutase, and total glutathione levels, with omics data supporting the involvement of phenylpropanoid and glutathione metabolism in Cd tolerance.^46,55–58^ Broader physiological surveys across native species further report dose–response patterns in antioxidant capacity, morpho-anatomical adjustment, and toxicity end points under mixed-metal tailings.^47,59^

Here, we investigate the metabolic responses of *V. polyanthes* and *P. aduncum* collected in tailings-affected and unaffected areas using untargeted liquid chromatography-tandem mass spectrometry (LC-MS^n^) followed by multivariate statistical analyses and molecular networking. Within an exploratory, hypothesis-generating framework, we assessed whether exposure to mining waste is associated with detoxification axes (glutathione and phenolic metabolism), while (i) characterizing exposure-associated chemical shifts, (ii) resolving shared versus species-specific responses, and (iii) highlighting stress-linked pathways.

## Results and Discussion

Initially, an unsupervised model (principal component analysis, PCA) was applied to visualize clustering tendencies and evaluate shifts in the chemical profiles of *V. polyanthes* and *P. aduncum* individuals collected from areas affected by the Fundão dam collapse and unaffected areas. For each species, PCA clearly differentiated plants growing in affected areas from those in unaffected areas, indicating significant differences in their chemical profiles and consequently broad metabolome shifts associated with exposure to mining waste. In *V. polyanthes*, PC1 and PC2 explained 70.40% and 5.73% of the variance, respectively, with affected plants scoring positively along PC1 (R^2^X= 0.792 and Q^2^= 0.710) (Supporting Information, Figure S1). In *P. aduncum*, differentiation occurred primarily along PC2 and PC3, which explained 9.50% and 3.30% of the total variance, respectively (R^2^X= 0.715 and Q^2^= 0.607) (Supporting Information, Figure S2).

To refine our analysis, we applied a supervised model (partial least squares discriminant analysis, PLS-DA) separately for each species to identify metabolites that discriminate plants from affected versus unaffected areas (Figure 2). The model demonstrated a clear distinction between the two groups (i.e., plants growing in affected or unaffected areas) (R^2^Y= 0.996 and Q^2^= 0.952 for *V. polyanthes*, and R^2^Y= 0.999 and Q^2^= 0.886 for *P. aduncum*), supporting the subsequent selection of the top discriminant metabolites.

**Figure 2:**
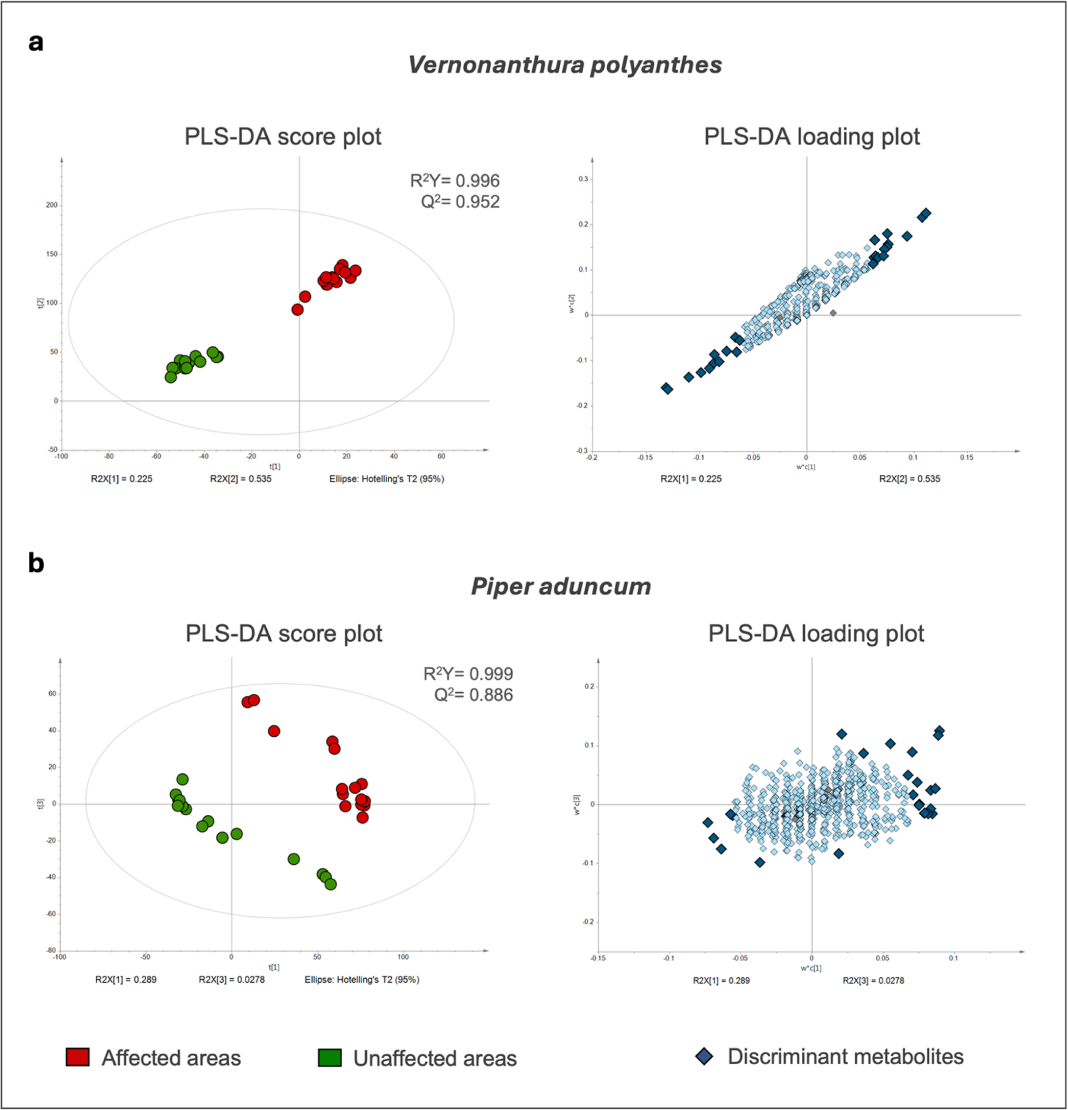
Partial least-squares discriminant analysis (PLS-DA) score and loading plots of LC-MS^n^ data for plants growing in areas affected by the Fundão dam collapse and in unaffected areas. **a**, PLS-DA for *Vernonanthura polyanthes* individuals. **b**, PLS-DA for *Piper aduncum* individuals. In the score plot, red dots represent the samples of individuals growing in areas affected by the Fundão dam collapse, and green dots represent samples of individuals growing in unaffected areas. In the loading plots, dark blue diamonds represent the variables (i.e., metabolites) responsible for discriminating samples between affected and unaffected areas for each plant species. R^2^Y, goodness of fit for the Y variables; Q^2^, goodness of prediction.

Based on the results of PLS-DA, which emphasized the separation between affected and unaffected samples, we assessed the Variable Importance in Projection (VIP) plot to determine discriminant metabolites. For each plant species, we selected the 25 most important variables (i.e., variables with the greatest VIP values), which were crucial for differentiating plants growing in affected or unaffected areas. Thereafter, we utilized the Global Natural Products Social Molecular Networking (GNPS) platform to create molecular networks for each species. In this process, feature-based molecular networking grouped MS^2^ spectra by shared fragments/neutral losses, enabling class-level context for discriminants.^60^

Discriminant metabolites were mapped in the molecular networks by enlarging the node sizes, which were colored based on mining waste exposure (red representing plants from affected areas and green representing plants from unaffected areas). Additionally, pie charts within these nodes were used to illustrate the relative abundance of metabolites in the plants growing in affected and unaffected areas (Supporting Information, Figures S3 and S4). This visualization provided an intuitive way of identifying potential metabolic pathways that were influenced by the presence of mining waste. The final step involved annotating these key metabolites based on their fragmentation profiles (i.e., MS^2^ data). These spectra were compared with reference spectral data deposited in public libraries, including MassBank, GNPS libraries/MASST, NuBBE_DB_, and SIRIUS.^61,62^ To further support the annotation indicated by the GNPS platform and Sirius prediction tools, we manually inspected the LC-MS^n^ data of each discriminant metabolite.

In *V. polyanthes*, among the top 25 discriminant metabolites, 56% were classified as peptides, 20% as phenolic compounds, 12% as peptide-phenolic conjugates, and 12% remained unclassified (Figure 3a). More specifically, we were able to confirm the annotation of 14 peptide-like derivatives (12 detected in plants from affected areas and two in plants from unaffected areas), five phenolic compounds (all detected in plants from unaffected areas), and three peptide-phenolic conjugates (all detected in plants from unaffected areas). For *P. aduncum*, phenolic compounds and terpenoids were indicated as the main discriminant metabolites. Within the 25 most important discriminant metabolites, 32% corresponded to phenolic compounds, 16% to terpenoids, and 12% to terpenoid-phenolic conjugates, while 40% could not be assigned to any class (Figure 3b). By manually inspecting the MS^n^ data, we confirmed the annotation of eight phenolic compounds (seven in plants from affected areas and one in plants from unaffected areas), four terpenoids (three in plants from affected areas and one in plants from unaffected areas), and three terpenoid-phenolic conjugates (two in plants from affected areas and one in plants from unaffected areas).

**Figure 3:**
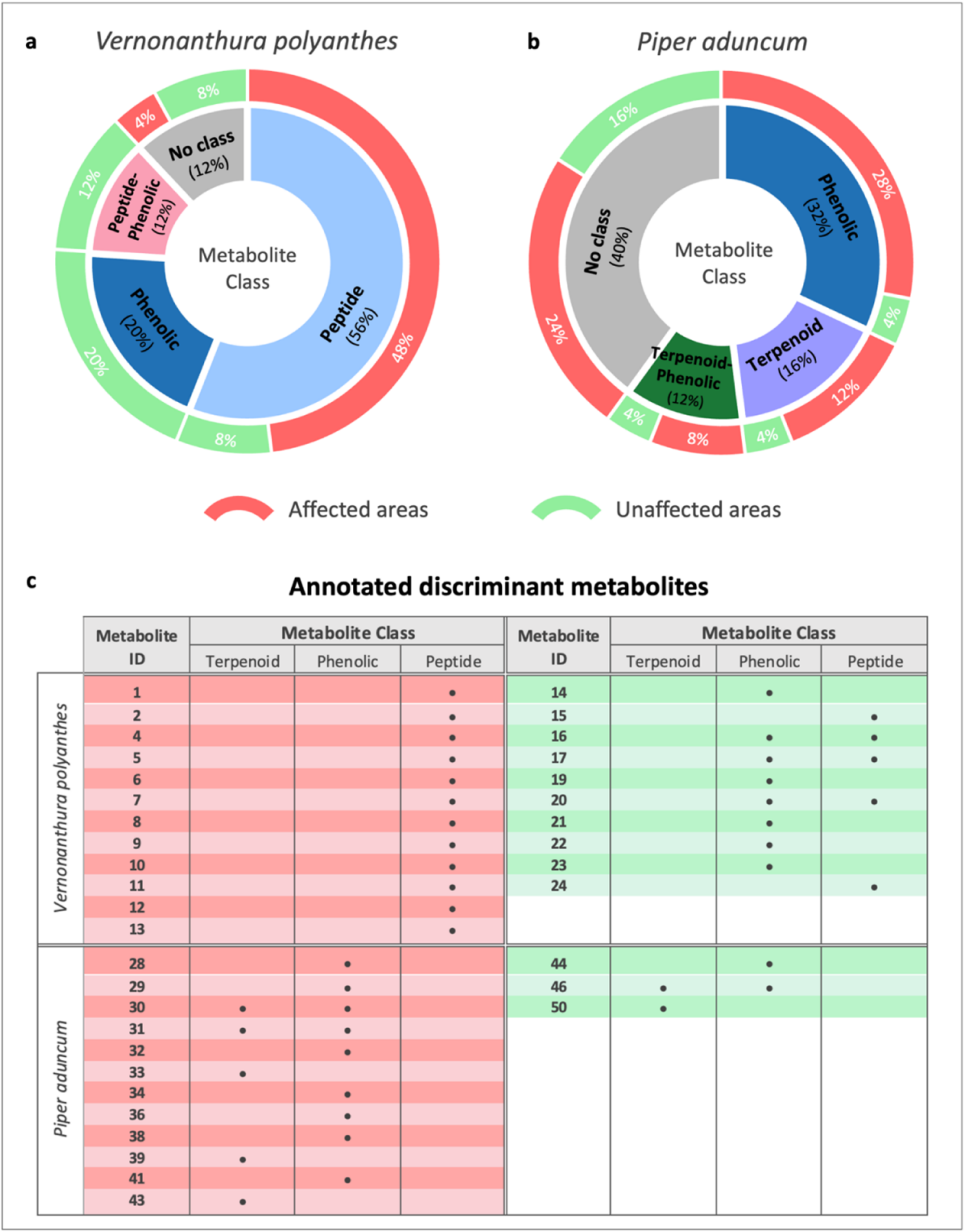
Metabolite classes of the top 25 selected discriminants for (**a**) *Vernonanthura polyanthes* and (**b**) *Piper aduncum* individuals collected in areas affected by the Fundão dam collapse and in unaffected areas. (**c**) Discriminant metabolites annotated for each plant species.

Interestingly, phenolic compounds were not assigned as discriminant metabolites for *V. polyanthes* individuals growing in areas affected by the Fundão dam collapse, whereas they did act as discriminants for *P. aduncum* individuals collected in those areas (Figure 3c). In *V. polyanthes*, phenolics appeared as discriminant metabolites only in individuals collected from unaffected areas.

Molecular networking analysis for *V. polyanthes* individuals enabled an intuitive visualization of the distribution of discriminant metabolites between plants from areas affected by the Fundão dam collapse and those from unaffected areas. Most of the annotated peptides were assigned as glutathione derivatives (metabolites **1**, **4**, **5**, **6,** and **11**). Additionally, peptides with an acetylated lysine moiety were also annotated (metabolites **4**, **6**, **11**, **12,** and **13**), as well as one dipeptide conjugated to a caffeoyl unit (metabolite **16**). All annotated phenolic compounds were assigned to the phenylpropanoid class and further classified as caffeoylquinic acid derivatives (metabolites **14**, **19**, **22,** and **23**) (Figure 4 and Supporting Information, Figures S5–S16). Spectrometric and spectroscopic data of the discriminant metabolites are presented in the Supporting Information (Table S1 and Figures S27–S51). These findings revealed that glutathione derivatives and/or lysine-acetylated peptides were enriched or majority detected in plants growing in affected areas, whereas phenylpropanoids, notably caffeoylquinic acid derivatives, prevailed in plants from unaffected areas. This profile is consistent with a chelation/sequestration-first strategy centered on the glutathione axis, with lysine acetylation signatures aligning with stress-related post-translational regulation.^63^

**Figure 4:**
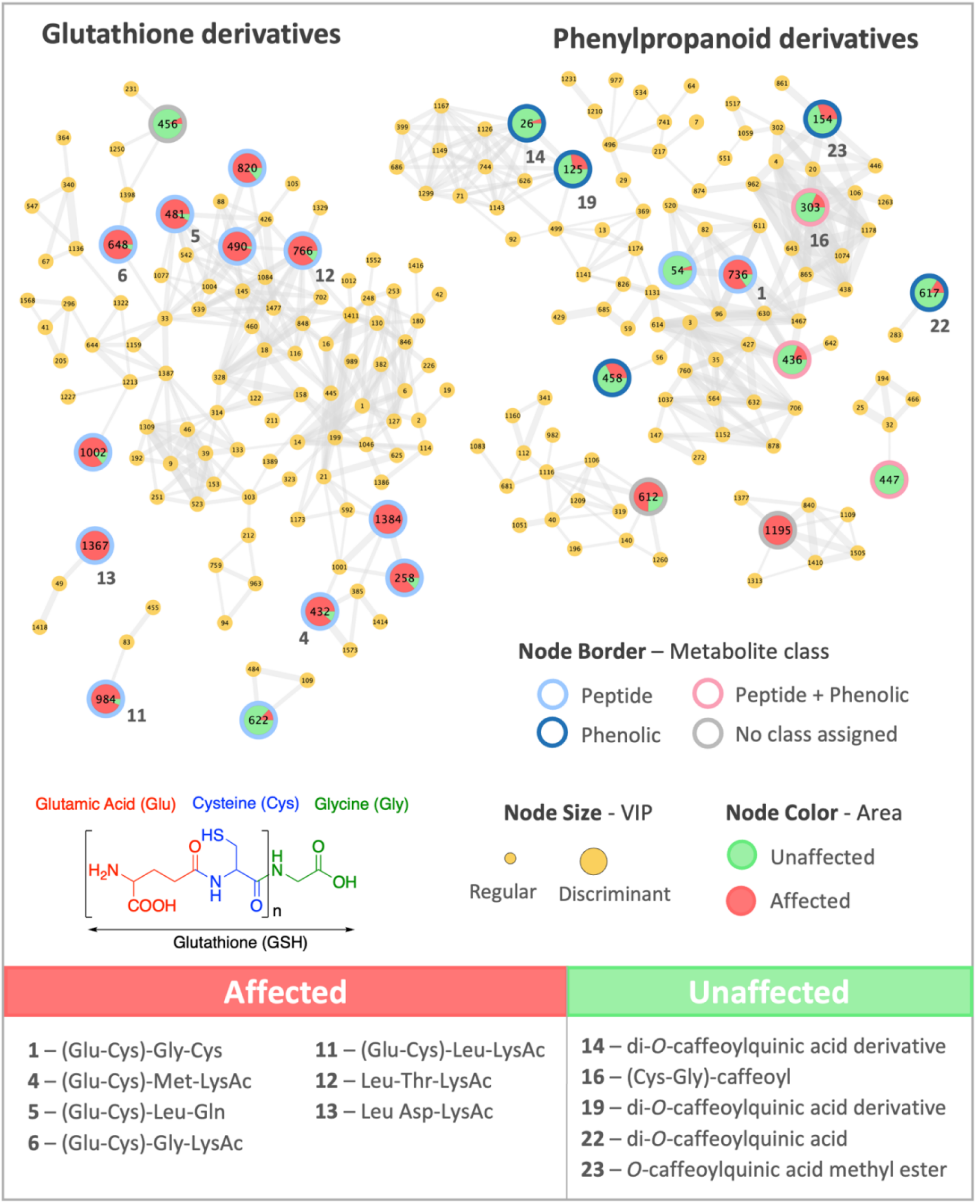
LC-MS^n^-based molecular networking for *Vernonanthura polyanthes*. Main clusters of the molecular networking for individuals grown in areas affected by the Fundão dam collapse (red nodes) and unaffected areas (green nodes), highlighting the annotated glutathione and phenylpropanoid derivatives. Large node sizes represent the top 25 discriminant metabolites. The backbone of phytochelatin-like derivatives, composed of glutamic acid, cysteine, and glycine residues, is displayed below the molecular networks. The main annotated discriminant metabolites for each area are shown at the bottom of the figure.

Interestingly, all annotated peptides (except metabolite **16,** a peptide conjugated with caffeoyl unit) were detected in *V. polyanthes* individuals collected in areas affected by the Fundão dam collapse, whereas all phenylpropanoid derivatives (including metabolite **16**) were detected in *V. polyanthes* individuals from unaffected areas. These findings indicated that biosynthetic pathways for nitrogen-based compounds, particularly peptides, may have been triggered as part of the adaptive metabolic responses of *V. polyanthes* to toxic mining waste exposure.

Analysis of sedimentary quality after the Fundão dam collapse revealed that the sediment profile contained high levels of diverse trace metals, such as Fe, Mn, Zn, Pb, Cu, Ni, and Cr, which reached contamination levels after the environmental disaster.^64^ Specifically, an increase in Cd content to levels above the National Environment Council limits was reported in the mining mud.^65^ In addition, further studies showed that Fe concentrations in river sediments remained elevated compared to precollapse levels.^11^ Although Fe is essential for enzymatic activities in plants, excessive free Fe disrupts cell redox balance and induces oxidative stress, thereby compromising plant survival and ecosystem stability.^66^

Studies have highlighted the protective roles of glutathione derivatives in enhancing plant resilience against stresses caused by metalloids. These compounds are crucial in the synthesis of sulfur-rich peptides and amino acids, as well as nitrogenous osmolytes that play significant roles in detoxification and antioxidant defense mechanisms.^67,68^ Additional research has also underscored the importance of glutathione derivatives in the chelation of heavy metals, providing an important defense mechanism against soil contamination.^69^ Therefore, the detection of glutathione derivatives as discriminant metabolites in *V. polyanthes* individuals collected in areas affected by the Fundão dam collapse suggests that these metabolites play a role in the plant’s defense response to toxic mining waste.

In addition, several of the annotated peptides and glutathione derivatives were characterized by the presence of an acetylated lysine moiety. Recent studies have revealed that lysine acetylation is an important post-translational modification that contributes to the regulation of plant stress adaptation by modulating fundamental biological processes, including signal transduction, energy metabolism, respiration, protein synthesis, and amino acid metabolism.^70,71^ Our findings suggest that lysine acetylation is involved in the adaptive response of *V. polyanthes* to toxic mining waste, given that lysine-acetylated derivatives were detected as discriminant metabolites exclusively in individuals collected from areas affected by the Fundão dam collapse. These patterns indicate that *V. polyanthes* prioritizes a peptide-centered detoxification strategy under tailings exposure.

For *P. aduncum*, molecular networking analysis revealed the presence of metabolites classified within the phenylpropanoid class and further characterized as flavonoids, including eight flavonoid glycosides derived from luteolin (metabolites **28**, **29**, **34,** and **41**) and apigenin (metabolites **32**, **36**, **38,** and **44**). Additionally, seven prenylated derivatives were annotated, including three prenylated flavonoids (metabolites **30**, **31,** and **46**) (Figure 5 and Supporting Information, Figures S17–S25). The discriminant metabolites, along with the corresponding spectrometric and spectroscopic data, are listed in the Supporting Information (Table S2 and Figures S52–S76).

**Figure 5:**
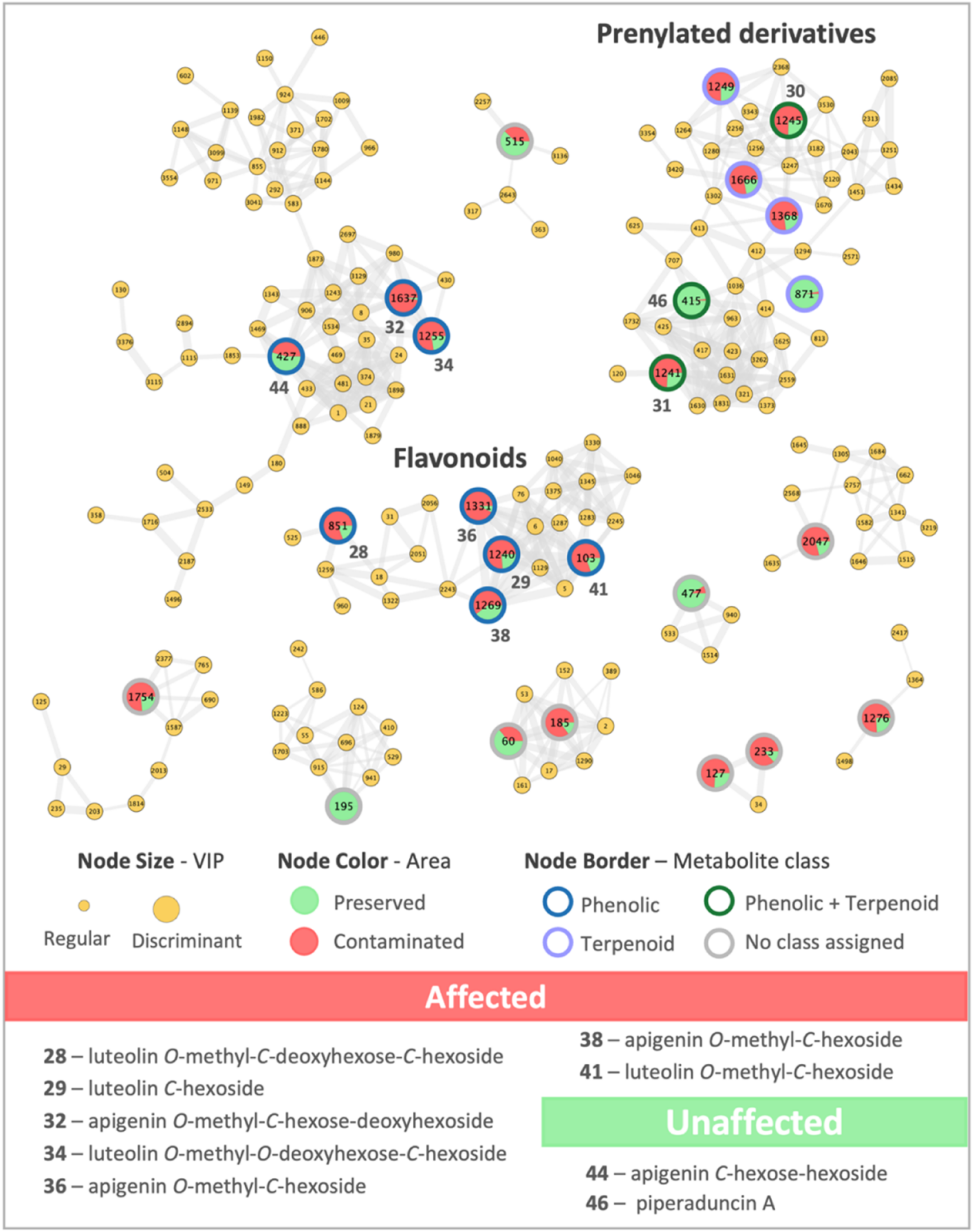
LC-MS^n^-based molecular networking for *Piper aduncum*. Main clusters of the molecular networking were obtained for individuals growing in areas affected by the Fundão dam collapse (red nodes) and unaffected areas (green nodes), highlighting the annotated flavonoids and prenylated derivatives. Large node sizes represent the top 25 discriminant metabolites. The main annotated discriminant metabolites for each area are shown at the bottom of the figure.

Flavonoid glycosides were detected as the main discriminant metabolites for *P. aduncum* individuals collected in areas affected by the Fundão dam collapse, with some prenylated phenolics also annotated as discriminants. Interestingly, individuals growing in affected areas were characterized mostly by the presence of flavonoid glycosides containing a methyl (CH_3_) group attached to the aglycone via an ether bond, while a flavonoid glycoside without an attached OCH_3_ group (apigenin *C*-hexose-hexoside, metabolite **44**) was annotated as discriminant for individuals growing in unaffected areas. Additionally, all annotated flavonoids exhibited at least one sugar moiety attached to the aglycone through a carbon–carbon bond (C–C bond).

At the structural level, *C*-glycosylation (C–C linkage, typically at C-6/C-8 of ring A) produces distinctive MS^2^ cross-ring cleavages and confers hydrolytic stability, enabling the compound to persist under acidic and oxidative conditions.^72^ By contrast, *O*-methylation attenuates phenolic acidity and increases hydrophobicity/membrane partitioning, potentially tuning radical-scavenging kinetics, while prenylation further enhances bilayer affinity.^73,74^ Functionally, this suite provides complementary protection in which polyhydroxylated cores support metal binding (catechol-type sites) and radical scavenging, while *O*-methylation/prenylation optimizes localization and reduces autoxidation. The combination of *O*-methylation, *C*-glycosylation, and prenylation confers increased hydrophobicity, stability, and membrane affinity to the metabolites, thereby enhancing their potential for redox buffering, metal complexation, and membrane protection under multimetal stress. Therefore, shifts in phenolic biosynthesis in *P. aduncum* indicate plant adaptations to oxidative stress, reflecting ecological responses to severe exposure to toxic mining waste. These alterations are supported by findings from various studies, which suggest that these metabolic adjustments are part of a broader strategy employed by plants to mitigate the adverse effects of environmental contaminants.^75,76^

## Conclusions

The catastrophic collapse of the Fundão dam, which dispersed over 40 million m^3^ of mining waste across the Doce River basin, resulted in profound and long-lasting impacts on both the environment and surrounding communities. Our findings revealed species-specific metabolic strategies in response to metal-induced environmental stress, with *V. polyanthes* enhancing peptide-based pathways, especially glutathione derivatives containing an acetylated lysine residue, and *P. aduncum* relying on phenolic compounds, notably *O*-methylated flavonoid *C*-glycosides and prenylated phenolics. Taken together, these profiles are consistent with a peptide-centered detoxification axis in *V. polyanthes* and a phenolic-centered redox/defense axis in *P. aduncum*, reflecting the two principal detoxification pathways reported for plants under multimetal stress. Additionally, the significant increase in peptides and phenylpropanoids represents an important adaptive response of these plant species, potentially contributing to ecosystem resilience and stability under environmental disturbance. Therefore, our study offers insights that advance the understanding of ecological stabilization and recovery after severe environmental disturbances, providing a framework for future investigations into plant resilience under multimetal stress conditions.

## Materials and Methods

### Plant Collection, Sample Preparation, and LC-MS^n^ Analyses

Leaves of *P. aduncum* and *V. polyanthes* were collected in areas affected by the Fundão dam collapse in the regions of Paracatu de Baixo (20°18′23“S 43°13′48”W) and Barra Longa (20°16′57“S 43°02′27”W) alongside the Gualaxo do Norte River, and in unaffected areas in the district of Monsenhor Horta (20°20′43“S 43°17′37”W), near to the Carmo River and to the city of Mariana, state of Minas Gerais, Brazil (Supplementary Material, Figure S26). Plant collections were conducted in August 2018. For each plant species, we collected full developed leaves of 15 individuals growing in affected areas and of 15 individuals growing in unaffected areas (i.e., a total of 30 individuals per species were sampled). Plant materials were deposited and registered in the José Baldini Herbarium at the Federal University of Ouro Preto (UFOP) under register OUPR 30686 for *V. polyanthes* and OUPR 30685 for *P. aduncum*. Details about the collection can be accessed and searched at https://specieslink.net. All samples were individually dried over silica gel at ambient conditions, powdered using a bench mill (A11 basic, IKA, Wilmington, NC, USA), and stored in sealed vials until further chemical extraction.

Plant extracts were prepared based on established protocols for plant metabolomics and analyzed by liquid chromatography coupled to an ultraviolet diode array detector and to an ion-trap mass spectrometer.^77–81^ Aliquots of 50 mg of each sample were weighed into microtubes and added to 1 mL of a mixture of methanol and water in the proportion of 3:1 (v:v). After vortex agitation (15 s, room temperature, *AV-2*, Gehaka, Grupo Kaufmann, São Paulo, SP, Brazil), the extracts were subjected to an ultrasonic bath (15 min, room temperature, *UltraSonic Cleaner* 1400, 40 kHz, UNIQUE, Indaiatuba, SP, Brazil) and centrifugation (25150g, 10 min, room temperature, *M-240R*, BOECO Germany, Hamburg, Germany), followed by filtration of the supernatant through PTFE syringe filters (0.22 μm, Analitica, Diadema, SP, Brazil) into glass vials.

LC-MS^n^ analyses were performed in a Shimadzu UFLC system (two LC20AD pumps, CTO20A oven, SIL20A automatic injector, and CBM20A controller) coupled to an ultraviolet diode array detector (UV-DAD) and to an ion-trap mass spectrometer (AmaZon SL, Bruker Daltonics Inc., Billerica, MA, USA). The samples were analyzed using a C18 column (5.0 μm, 250 × 4.6 mm, Phenomenex, Torrance, CA, USA) and eluted with a gradient of water (solvent A) and acetonitrile (solvent B), both containing 0.1% of formic acid. The mobile phase flow was set at 1 mL·min^–1^, and the following gradient was employed: 0–1 min (10% B), 1–45 min (10% to 100% B), 45–55 min (100% B), 55–58 min (100%–10% B), and 58–63 min (10% B).

The mass spectrometer was operated in positive ionization mode with the following parameters: capillary voltage, 3.5 kV; nebulizer pressure, 60 psi; N_2_ as drying gas at a flow of 10 L.min^–1^; drying temperature, 330 °C; auto MS/MS acquiring data between *m*/*z* 50 and 1300, with an average of 3 spectra per second; UltraScan mode for MS/MS; acquisition at the rate of 3 spectra per second; exclusion of a particular ion after 3 spectra for 30 s. The mass spectrometer was controlled with Hystar software (Bruker Daltonics Inc., Billerica, MA, USA). To minimize acquisition-order effects, injections were randomized within species and exposure status (affected/unaffected areas). Solvent blanks were also included to monitor background signals throughout the batch.

### Molecular Networking and Multivariate Statistical Analyses

The molecular networking and multivariate statistical analyses were based on parameters previously used by our group and other research groups.^82–84^ The LC-MS^n^ data were converted to *.mzXML format using MSConvert software (version 3 for Windows, Proteowizard Software Foundation, Palo Alto, CA, USA). The resulting data of *V. polyanthes* and *P. aduncum* were separately processed by MzMine software (version 2.51 for Windows, BMC Bioinformatics, United Kingdom) using the following parameters: mass detection, mass detector – centroid (noise level, 1.0E^5^ for MS level 1 and 1.0E^4^ for MS level 2); ADAP chromatogram builder (min group size in # of scans, 5; group intensity threshold, 1.0E;^5^ min highest intensity, 1.0E;^6^ *m*/*z* tolerance, 0.3 *m*/*z* or 0 ppm); chromatogram deconvolution, algorithm – wavelets (ADAP) (S/N threshold, 10; S/N estimator, intensity window SN; min feature height, 1.0E;^7^ coefficient/area threshold, 50; peak duration range, 0.2–2.0; RT wavelet range, 0.02–0.20), *m*/*z* center calculation – median, *m*/*z* range for MS^2^ scan pairing, 0.3; RT for MS2 scan pairing, 0.2; isotopic peak grouper (*m*/*z* tolerance, 0.5 *m*/*z* or 0 ppm; retention time tolerance, 0.2 min (absolute); maximum charge, 2; representative isotope, most intense); alignment, join aligner (*m*/*z* tolerance, 0.3 *m*/*z* or 0 ppm; weight for *m*/*z*, 50; retention time tolerance, 0.2 min (absolute); weight for retention time, 50). After processing, data were exported as a *.mgf file and *.csv quantification table for GNPS and multivariate analyses.

Multivariate statistical analyses were performed using SIMCA software (ver. 13.0.3.0, Umetrics, Sweden). Unsupervised (PCA) and supervised (PLS-DA) statistical analyses were carried out for each plant species separately, using the *.csv quantification table, which was previously log10-transformed. The classes for PLS-DA were determined according to the location where the samples were collected, representing unaffected (group 1) and affected areas (group 2). Discriminant metabolites were identified based on the PLS-DA loading plot and the VIP plot, considering the 25 variables with the highest VIP values.

The molecular networks for *V. polyanthes* and *P. aduncum* were constructed using the GNPS online platform (https://gnps.ucsd.edu/ProteoSAFe/static/gnps-splash.jsp). Using the advanced analysis tools, we uploaded the *.mgf file and *.csv quantification table and performed feature-based molecular networking. The following parameters were employed: quantification table source, MZmine; precursor ion mass tolerance, 2.0 Da; fragment ion mass tolerance, 0.5 Da; min pairs cosine score, 0.7; minimum matched fragment ions, 4; maximum shift between precursors, 500 Da; network topK, 10; maximum connected component size, 100; library search min matched peaks, 4; score threshold, 0.7; search analogues, do not search; maximum analog search mass difference, 100 Da; top results to report per query, 1; minimum peak intensity, 0; filter precursor window, filter; filter library, filter library; filter peaks in 500 Da window, filter; normalization per file, no norm; aggregation method for peak abundances per group, sum; PCoA distance metric, cosine; run stats and plots, no; run dereplicator, run. The resulting molecular networks were edited and analyzed with Cytoscape (version 3.8, Institute for Systems Biology, Seattle, WA, USA). All raw data, including spectrometric and spectroscopic information, as well as the processed data (.mgf files and .csv spreadsheets) used as inputs for molecular network and multivariate analyses were deposited at the Mass Spectrometry Interactive Virtual Environment (MassIVE) website. The publicly available data set can be accessed at http://massive.ucsd.edu under the identifier MSV000095153 (doi:10.25345/C5FB4WZ0T).

Annotation of the discriminant metabolites was carried out by visually inspecting and interpreting the chromatographic data (retention time) along with the spectroscopic (*m*/*z* and fragmentation pattern) and spectroscopic (UV absorption) data of each selected variable (i.e., discriminant metabolite). When sufficient information was available, metabolites were assigned to major classes, terpenoids, peptides, and phenolic compounds, and further categorized into subclasses such as flavonoids, phenylpropanoid derivatives, and glutathione derivatives, with their respective names and molecular formulas reported. Considering that most terpenoids exhibit minimal absorption in the UV range, except for highly conjugated terpenoids, which typically absorb between 400 and 500 nm, terpenoids were characterized based on the occurrence of neutral losses of 56 Da in their MS^n^ spectra. These neutral losses typically correspond to the cleavage of prenyl side chains or related structures, resulting in a C_4_H_8_ fragment that serves as a diagnostic feature for identifying terpenoid-related molecules (i.e., those containing isoprene units as structural building blocks) [87]. Peptides were determined based on characteristic neutral losses in the MS/MS spectra–such as 146 Da for glutamylcysteine (GluCys) or glutathione residues, 129 Da for aliphatic glutathione residues, 178 Da for glycylcysteine (GlyCys) residues, 86 Da for acetylated lysine (LysAc) residues, and 42 Da for *N*-acetyl derivatives) and typical UV absorption between 214 and 220 nm [88,89]. Phenolic compounds were determined based on their characteristic UV absorption, attributed to aromatic rings and conjugated systems, which typically exhibit two maximum absorbance bands around 270–295 nm and 320–330 nm [90,91]. Confidence level for metabolite annotation was determined according to the proposed minimum reporting standards for chemical analysis implemented by the Chemical Analysis Working Group (CAWG) Metabolomics Standard Initiative (MSI) [92,93]. For flavonoids, positions of the substituents were not assigned. Sugar moieties were indicated as hexoses or deoxyhexoses (i.e., no stereochemistry determination was carried out).

## Supplementary Material



## Notes

Additional multivariate statistical analyses and information for metabolite annotation, including score plots of the principal component analysis, full LC-MS^n^-based molecular networks, and tables with spectrometric and spectroscopic data, along with MS, MS/MS, and UV spectra for all discriminant metabolites (PDF)
