# Supplementary material for: Brazilian Mining Dam Collapse: Molecular Networking–Guided Metabolomics Reveals Species-Specific Plant Detox
Source: ACS Omega. 2026 Jan 6;11(9):14835–46. doi: 10.1021/acsomega.5c11096 (PMC12980215; doi:10.1021/acsomega.5c11096)
Supplement: Supplementary file 1 [file ao5c11096_si_001.pdf]

SUPPORTING INFORMATION

Brazilian Mining Dam Collapse: Molecular Networking–Guided Metabolomics Reveals Species-Specific Plant Detox

Marília Elias Gallon[1§], Eduardo Afonso Silva-Junior[2§], Amanda Roberta Corrado[3], Maria das Graças Lins Brandão[3], Maria Cristina Teixeira Braga Messias[4], Alberto José Cavalheiro[5], Norberto Peporine Lopes[1], Alan Cesar Pilon[5*]

1- University of Sao Paulo, Faculty of Pharmaceutical Sciences of Ribeirao Preto, BioMolecular Sciences, Prof. Dr. Zeferino Vaz Avenue, Ribeirao Preto, 14040-903, Brazil.

2- Federal University of Mato Grosso, Unidade II, N 6390 Valdon Varjão Avenue, Barra do Garças, 78600-000, Brazil

3- Federal University of Minas Gerais, Faculty of Pharmacy, Centro Especializado em Plantas Aromáticas, Medicinais e Tóxicas, Museu de História Natural e Jardim Botânico de Belo Horizonte, 1035 Gustavo da Silveira Street, Belo Horizonte, 31270-901, Brazil.

4- Federal University of Ouro Preto, Institute of Exact and Biological Sciences, Biodiversidade, Evolução e Meio Ambiente, 786 Quatro Street, Ouro Preto, 35402-136, Brazil.

5- São Paulo State University, Institute of Chemistry, Biochemistry and Organic Chemistry, 55 Francisco Degni Street, Araraquara, 14800-060, Brazil.

[§] These authors equally contributed to this work.

*Correspondence to alan.pilon@unesp.br

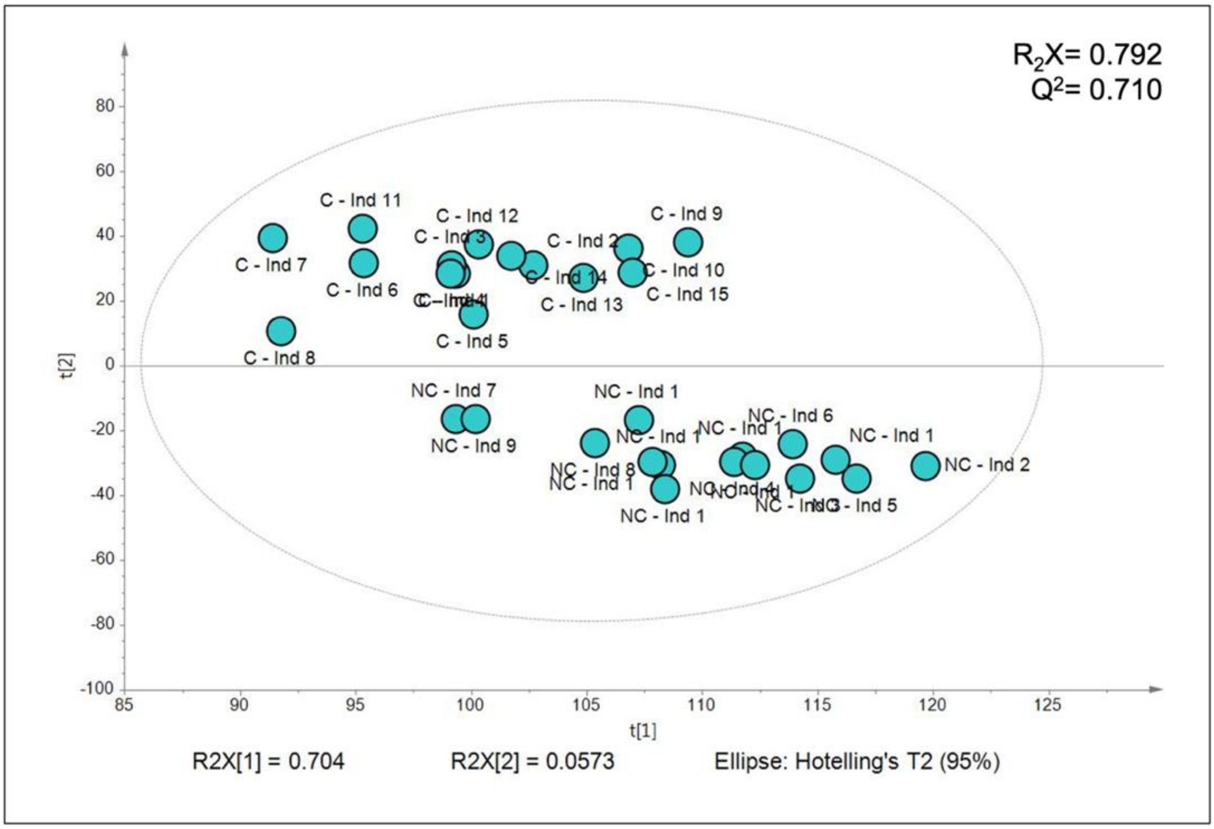

Figure S1. Principal component analysis plot for Vernonanthura polyanthes samples analyzed by LC-MS^n (ion-trap) in the positive ionization mode, showing a clustering tendency based on the location where the samples were collected (samples from affected areas were clustered in the upper part of the plot while samples from unaffected areas were clustered in the lower part of the plot). C, indicates samples from affected areas; NC, indicates samples from unaffected areas; Ind, indicates the plant individual being represented.

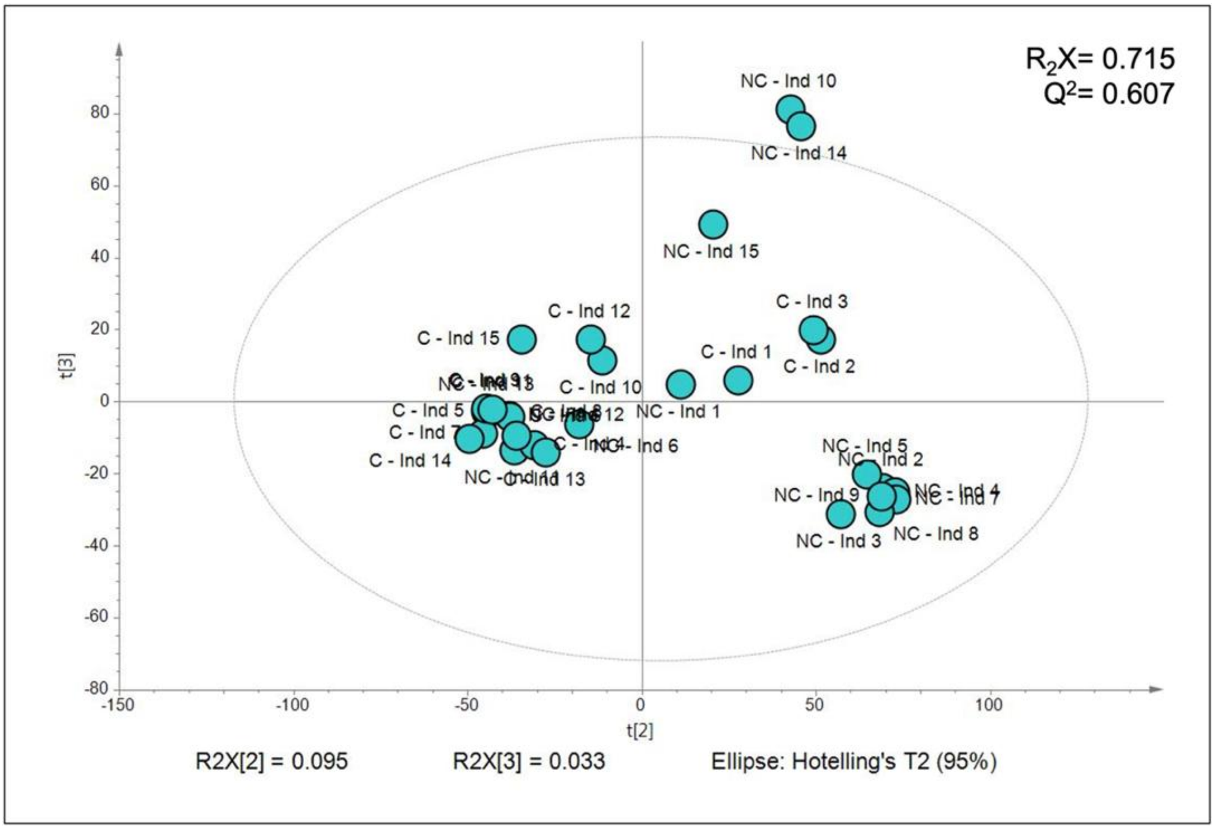

Figure S2. Principal component analysis plot for *Piper aduncum* samples analyzed by LC-MS^n (ion-trap) in the positive ionization mode, showing a clustering tendency based on the location where the samples were collected (samples from affected areas were mostly clustered in the left quadrants of the plot while samples from unaffected areas were mostly clustered in the lower right quadrant of the plot). C, indicates samples from affected areas; NC, indicates samples from unaffected areas; Ind, indicates the plant individual being represented.

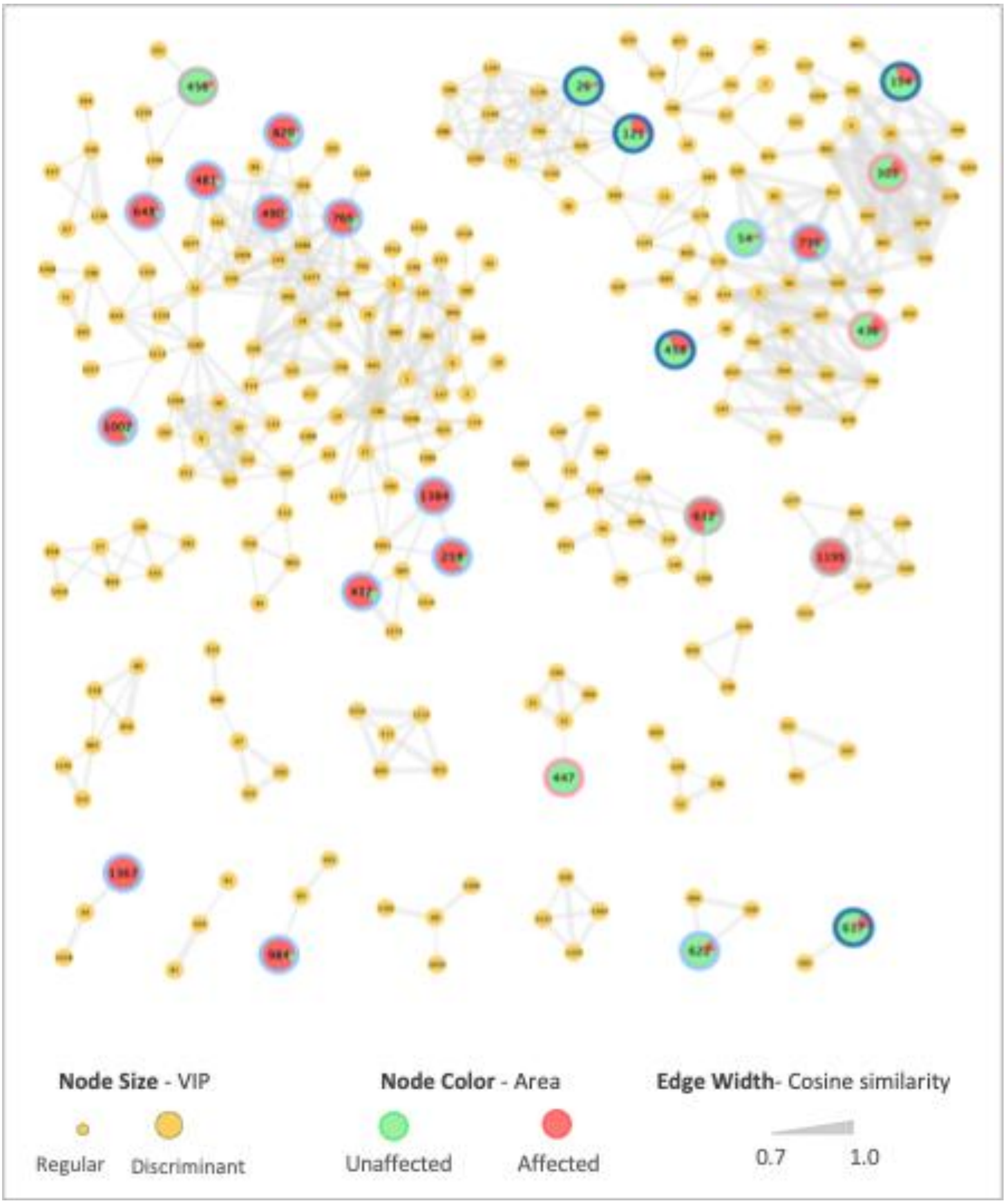

Figure S3. LC-MS^n-based molecular networking for samples of Vernonanthura polyanthes collected near the Gualaxo do Norte River after the Fundão dam collapse. Red and green nodes represent samples from affected and unaffected areas, respectively. Large nodes represent the 25 most important variables appointed by the PLS-DA model as discriminants between affected and unaffected areas. The width of the edges represents the degree of similarity among the nodes in the molecular network.

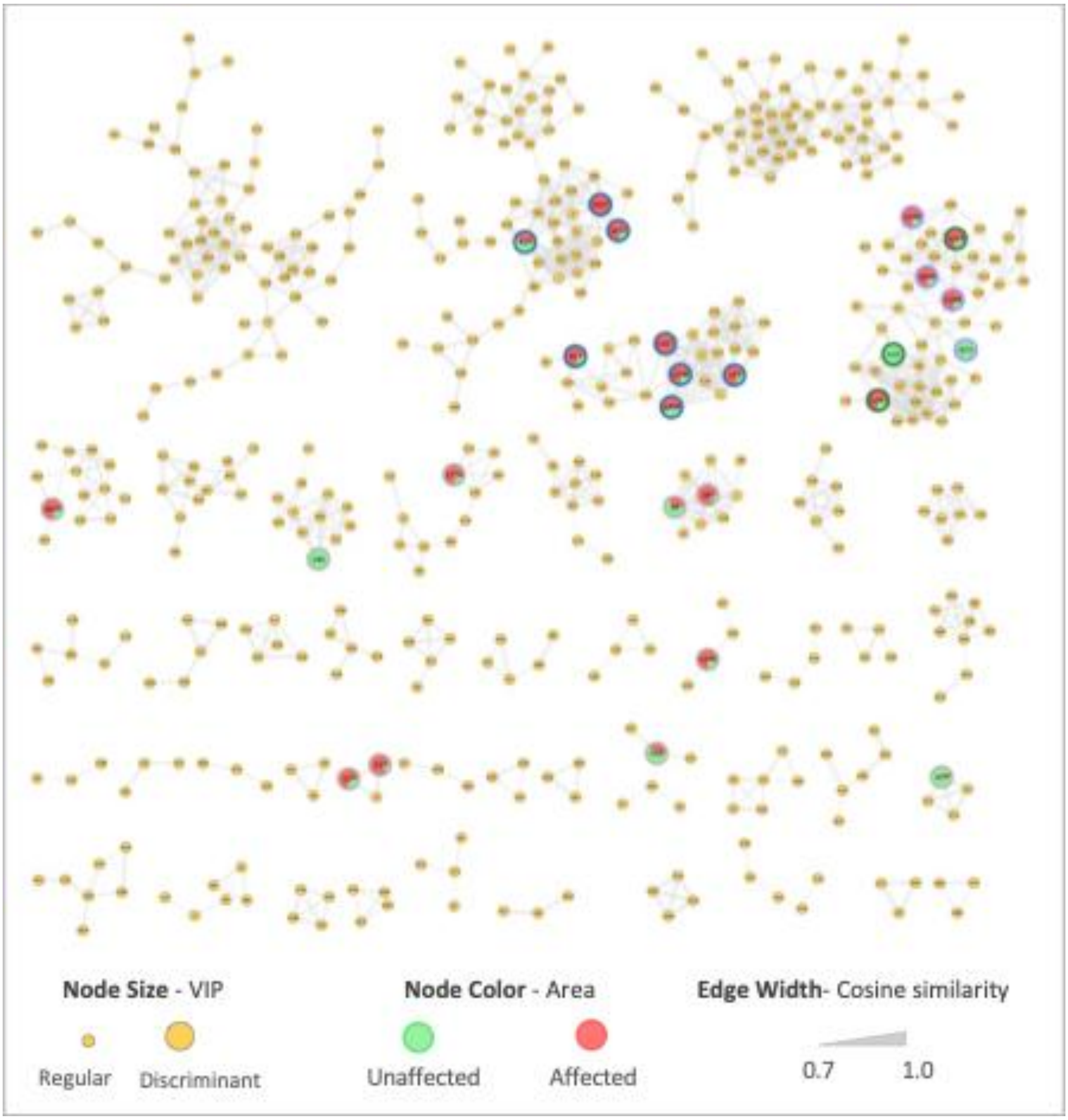

Figure S4. LC-MS^n-based molecular network for samples of Piper aduncum collected near the Gualaxo do Norte River after the Fundão dam collapse. Red and green nodes represent samples from affected and unaffected areas, respectively. Large nodes represent the 25 most important variables appointed by the PLS-DA model as discriminants between affected and unaffected areas. The width of the edges represents the degree of similarity among the nodes in the molecular network.

Metabolite 1: (Glu-Cys)-Gly-Cys

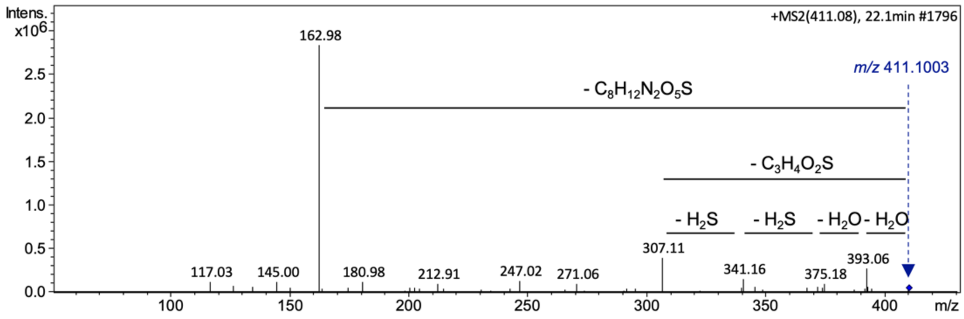

Protonated molecule

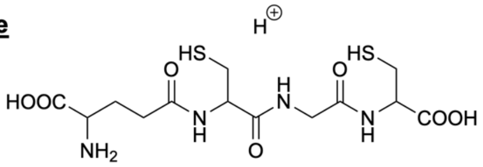

m/z 411.1003

Losses of H_2O

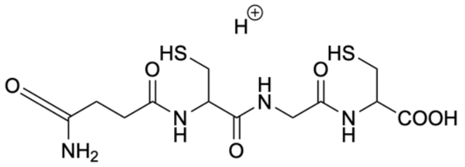

m/z 393

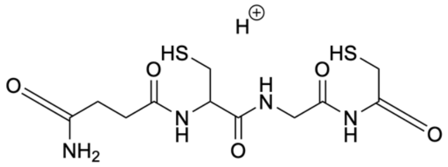

m/z 375

Losses of H_2S

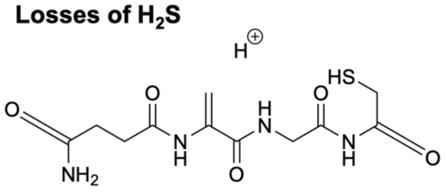

m/z 341

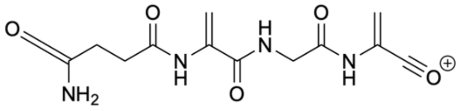

m/z 307

Loss of $C_3H_4O_2S$

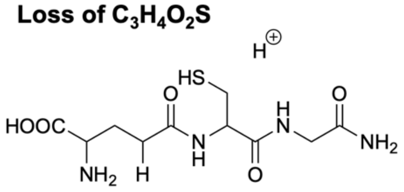

m/z 307

Loss of $C_8H_{12}N_2O_5S$

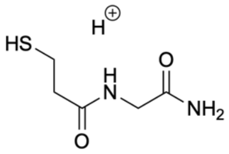

m/z 163

Figure S5: MS^2 spectrum, protonated molecule and fragments of metabolite **1**, annotated as (Glu-Cys)-Gly-Cys in level 2 of confidence.

Metabolite 4: (Glu-Cys)-Met-LysAc

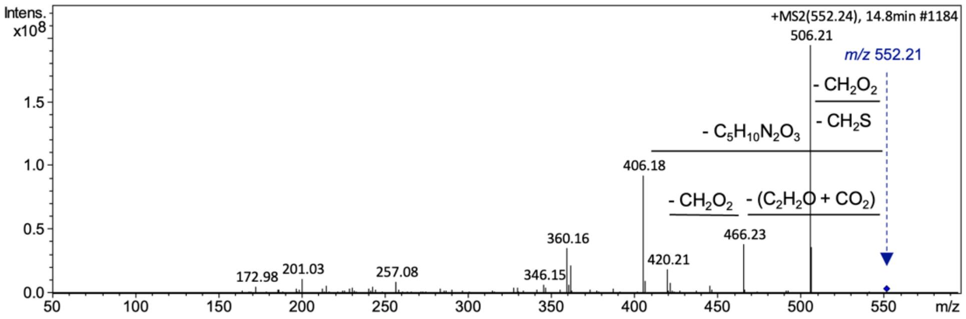

Protonated molecule

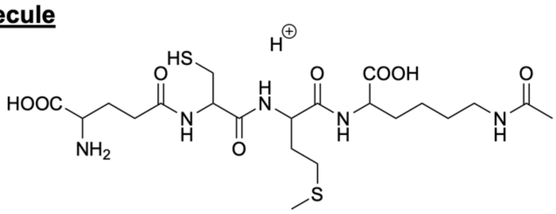

m/z 552.2156

Loss of CH_2O_2

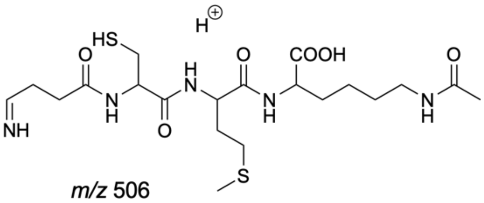

m/z 506

Loss of CH_2S

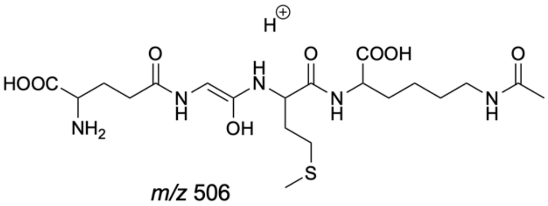

m/z 506

Loss of $C_2H_2O + CO_2$

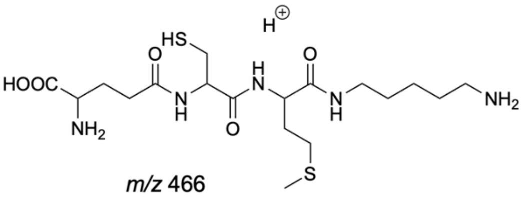

m/z 466

Loss of $C_2H_2O + CO_2 + CH_2O_2$

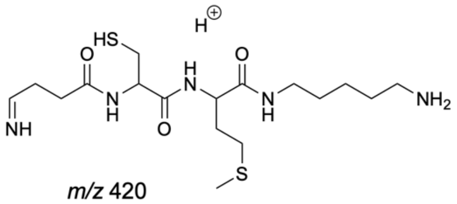

m/z 420

Loss of $C_5H_{10}N_2O_3$

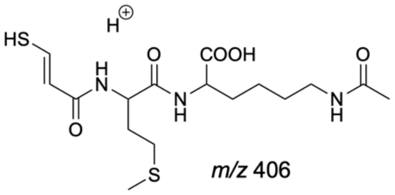

m/z 406

Figure S6: MS^2 spectrum, protonated molecule and fragments of metabolite **4**, annotated as (Glu-Cys)-Met-LysAc in level 2 of confidence.

Metabolite 5: (Glu-Cys)-Leu-Gln

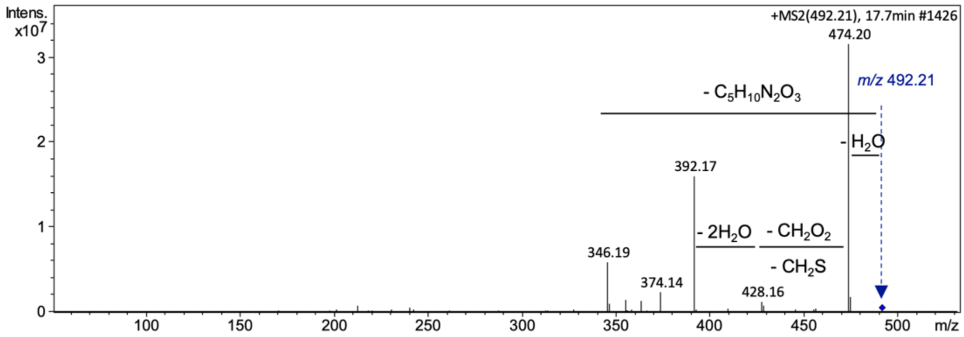

Protonated molecule

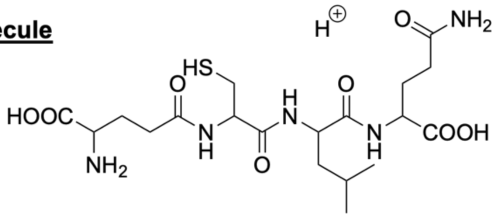

m/z 492.2123

Loss of H_2O

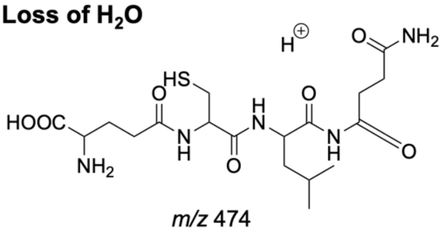

m/z 474

Loss of CH_2O_2

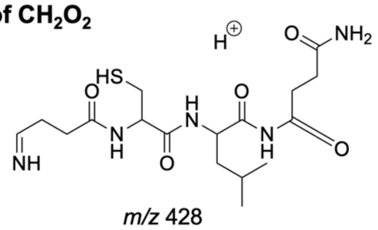

m/z 428

Loss of CH_2S

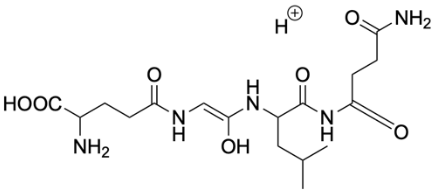

m/z 428

Loss of CH_2S followed by loss of two H_2O

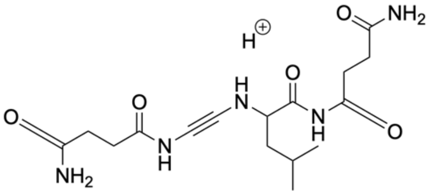

m/z 392

Loss of $C_5H_{10}N_2O_3$

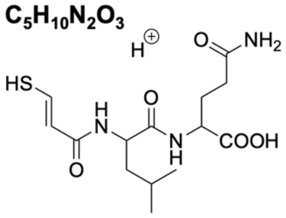

m/z 346

Figure S7: MS^2 spectrum, protonated molecule and fragments of metabolite **5**, annotated as (Glu-Cys)-Leu-Gln in level 2 of confidence.

Metabolite 6: (Glu-Cys)-Gly-LysAc

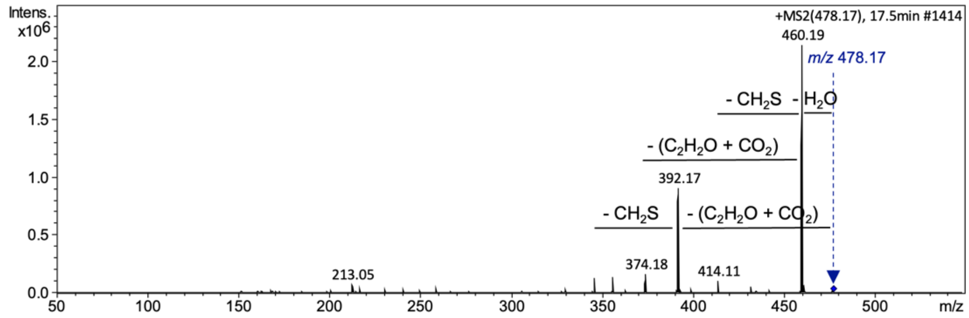

Protonated molecule

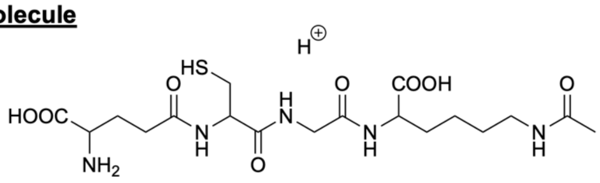

m/z 478.1966

Loss of H_2O

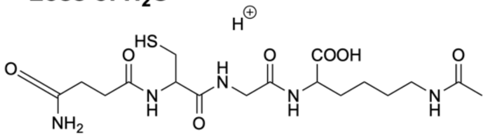

m/z 460

Loss of H_2O followed by CH_2S

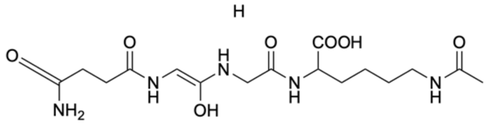

m/z 414

Loss of H_2O followed by $C_2H_2O + CO_2$

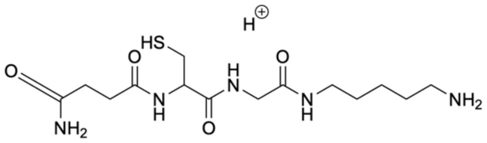

m/z 374

Loss of $C_2H_2O + CO_2$

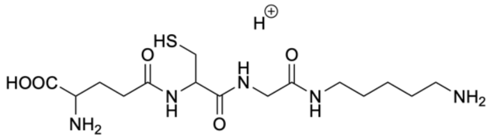

m/z 392

Loss of $C_2H_2O + CO_2$ followed by CH_2S

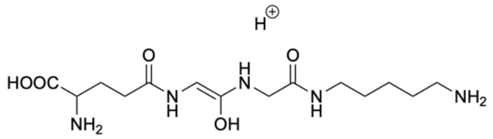

m/z 346

Figure S8: MS^2 spectrum, protonated molecule and fragments of metabolite **6**, annotated as (Glu-Cys)-Gly-LysAc in level 2 of confidence.

Metabolite 11: (Glu-Cys)-Leu-LysAc

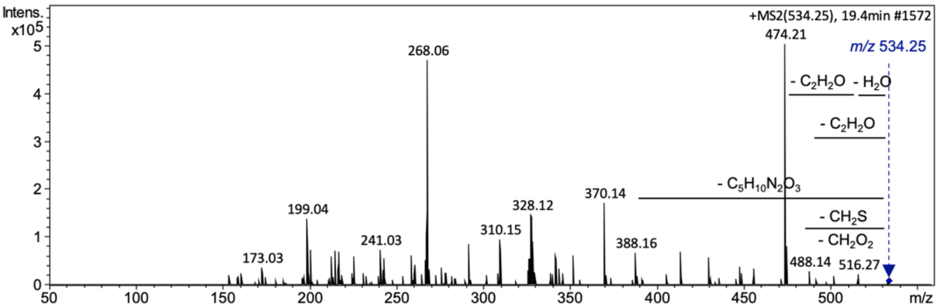

Protonated molecule

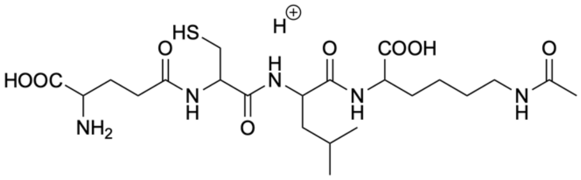

m/z 534.2592

Loss of H_2O

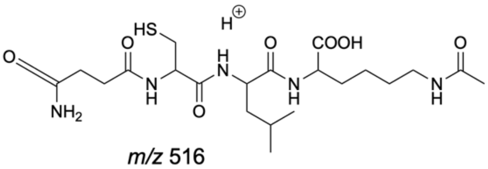

m/z 516

Loss of C_2H_2O

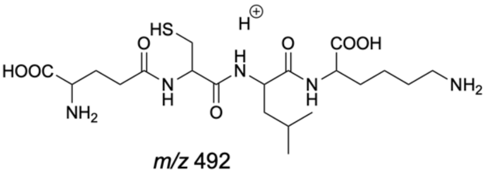

m/z 492

Loss of CH_2O_2

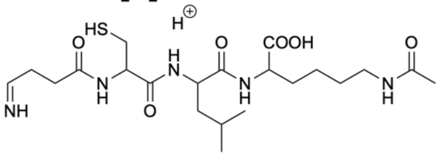

m/z 488

Loss of CH_2S

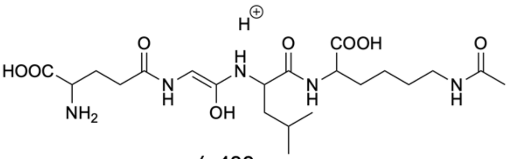

m/z 488

Loss of H_2O followed by C_2H_2O

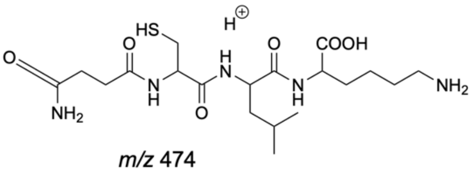

m/z 474

Loss of glutamic acid residue ($C_5H_{10}N_2O_3$)

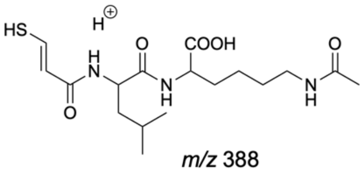

m/z 388

Figure S9: MS^2 spectrum, protonated molecule and fragments of metabolite **11**, annotated as (Glu-Cys)-Leu-LysAc in level 2 of confidence.

Metabolite 12: Leu-Thr-LysAc

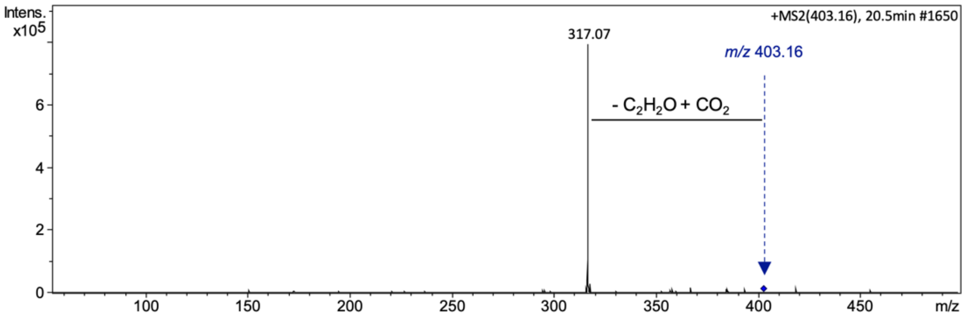

Protonated molecule

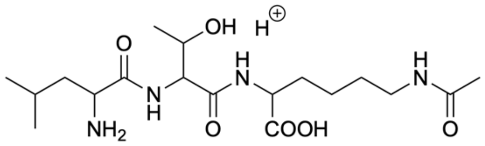

m/z 403.2551

Loss of $C_2H_2O + CO_2$

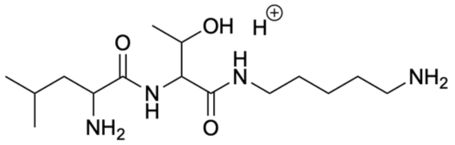

m/z 317

Figure S10: MS^2 spectrum, protonated molecule and fragments of metabolite **12**, annotated as Leu-Thr-LysAc in level 2 of confidence.

Metabolite 13: Leu-Asp-LysAc

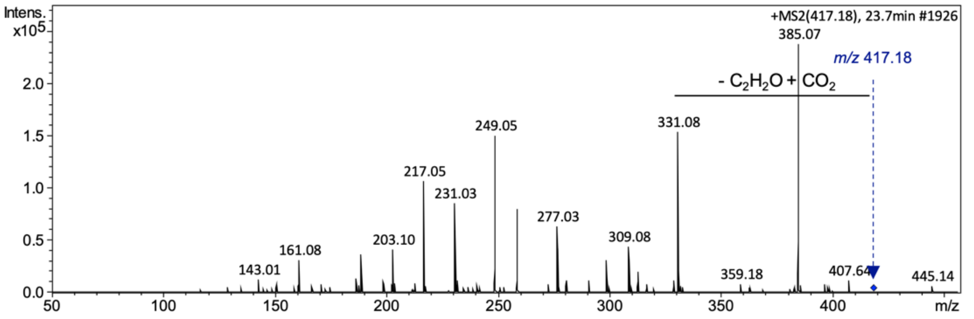

<u>**Protonated molecule**</u>

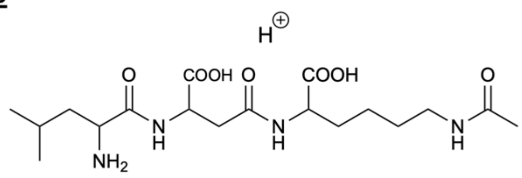

m/z 417.2344

Loss of $C_2H_2O + CO_2$

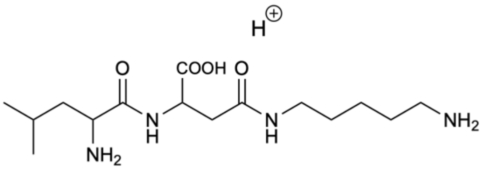

m/z 331

Figure S11: MS^2 spectrum, protonated molecule and fragments of metabolite **13**, annotated as Leu-Asp-LysAc in level 2 of confidence.

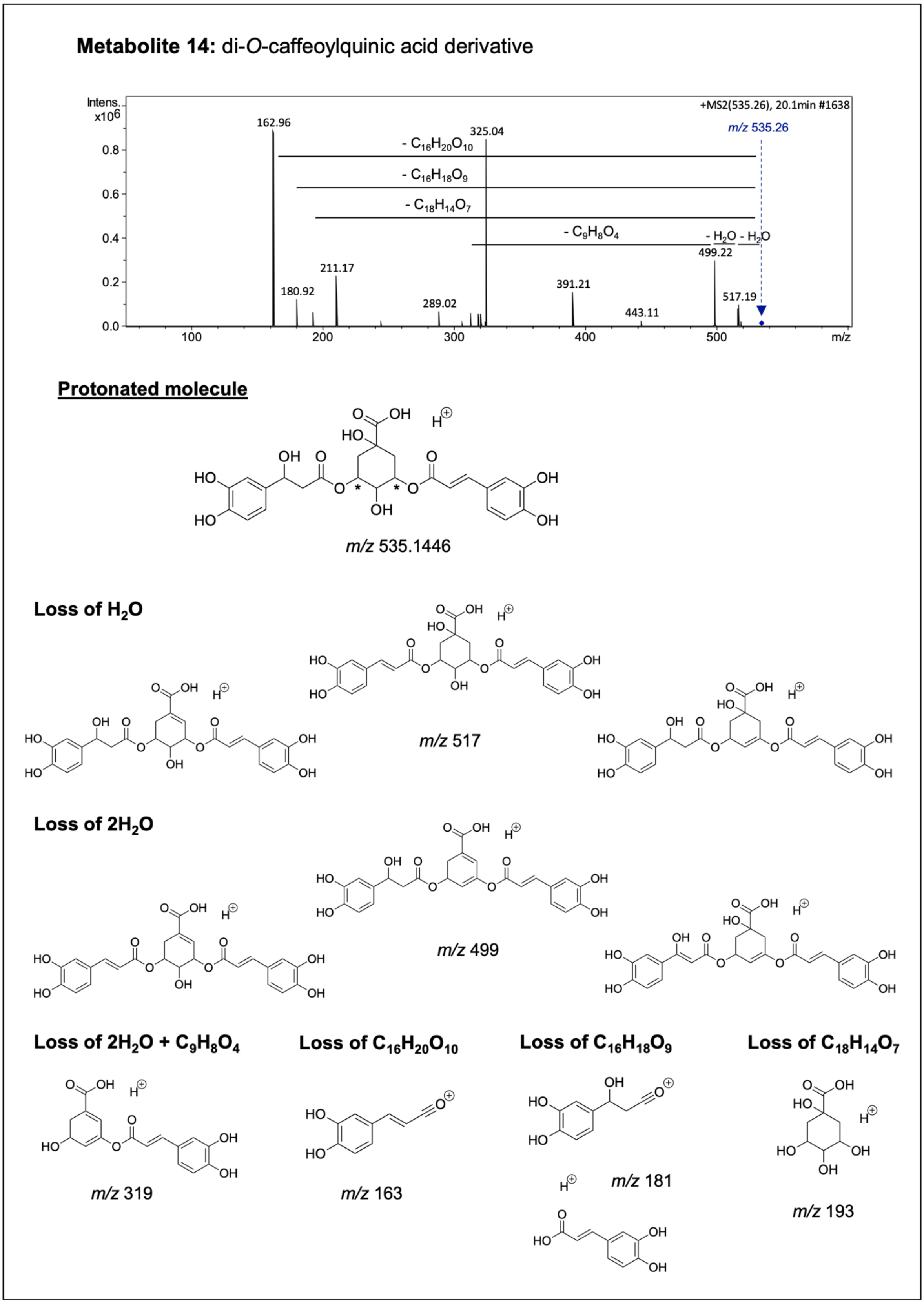

Figure S12: MS^2 spectrum, protonated molecule and fragments of metabolite **14**, annotated as di-*O*-caffeoylquinic acid derivative in level 2 of confidence. *Positions of the caffeoyl groups could not be assigned with confidence and were represented only for illustrating purposes.

Metabolite 16: (Cys-Gly)-caffeoyl

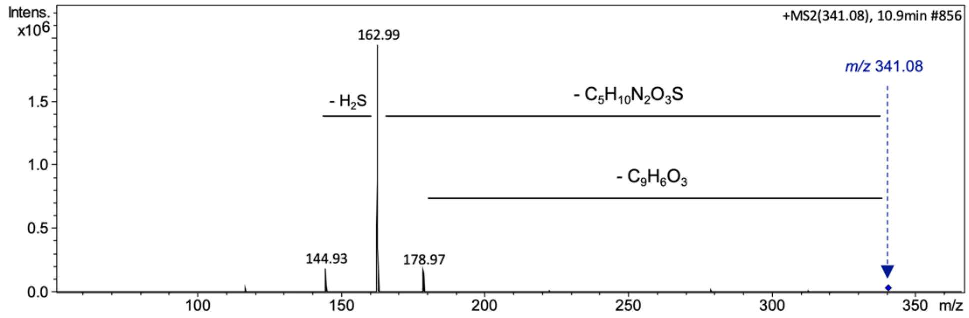

Protonated molecule

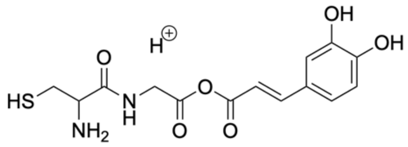

m/z 341.0802

Loss of $C_9H_6O_3$

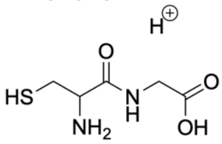

m/z 179

Loss of $C_5H_{10}N_2O_3S$

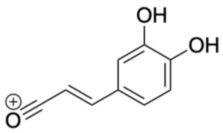

m/z 163

Loss of $C_9H_6O_3$ + H_2S

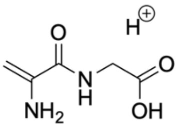

m/z 145

Figure S13: MS^2 spectrum, protonated molecule and fragments of metabolite **16**, annotated as (Gly-Cys)-caffeoyl in level 2 of confidence.

Metabolite 19: di-*O*-caffeoylquinic acid derivative

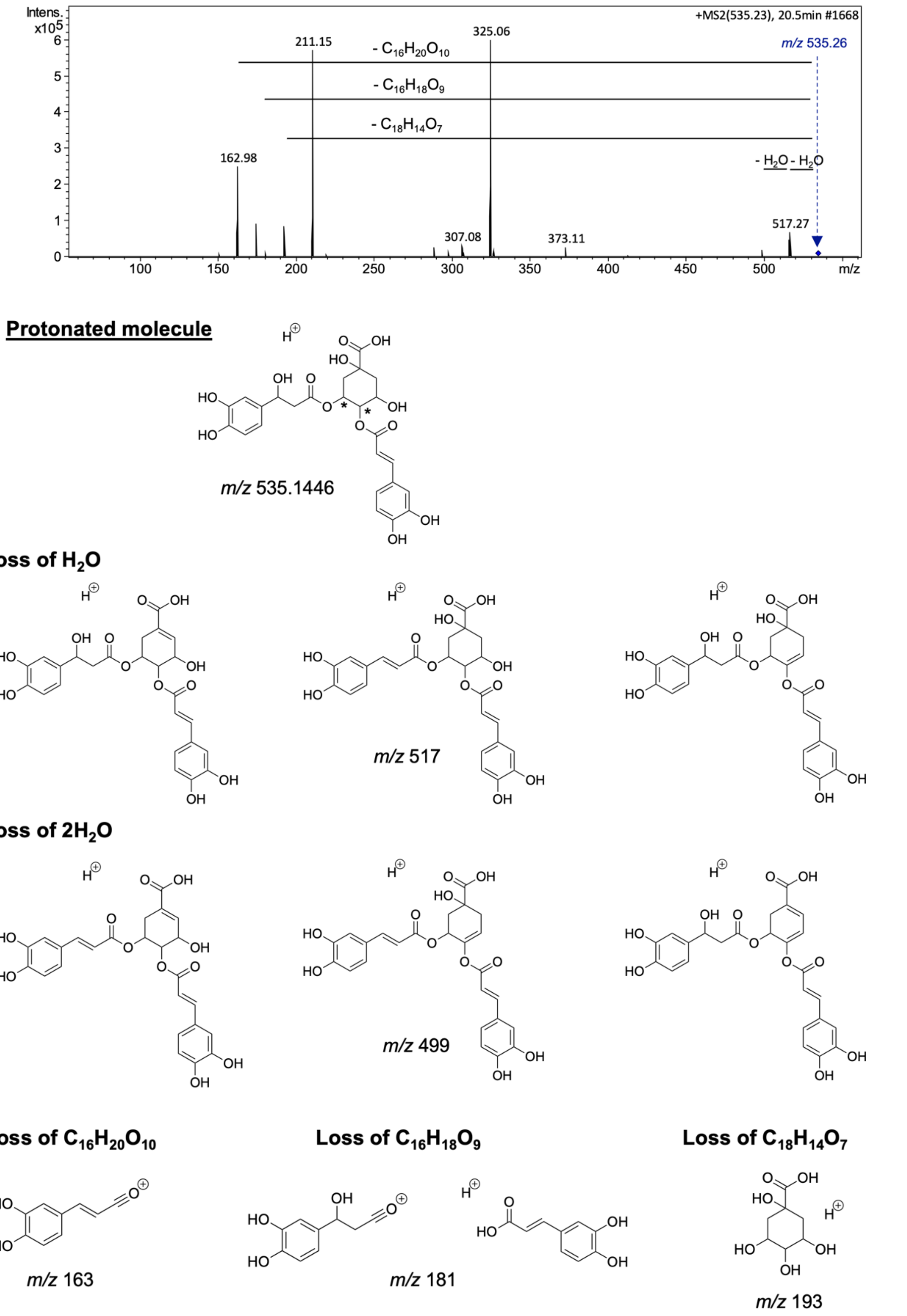

Figure S14: MS^2 spectrum, protonated molecule and fragments of metabolite **19**, annotated as di-*O*-caffeoylquinic acid derivative in level 2 of confidence. *Positions of the caffeoyl groups could not be assigned with confidence and were represented only for illustrating purposes.

Metabolite 22: di-*O*-caffeoylquinic acid

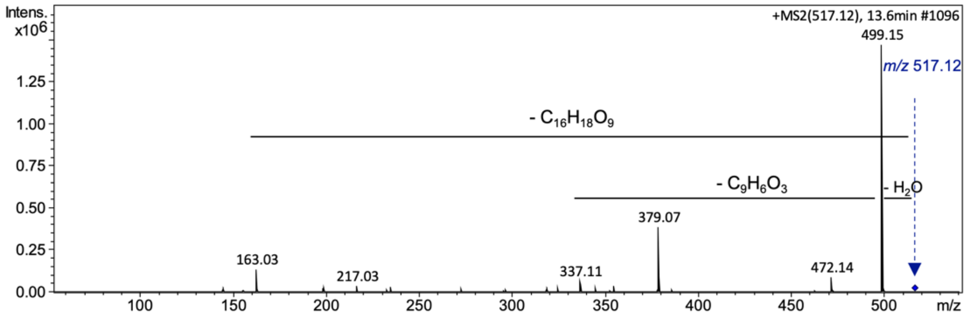

Protonated molecule

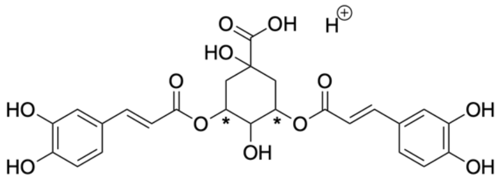

m/z 517.1341

Loss of H_2O

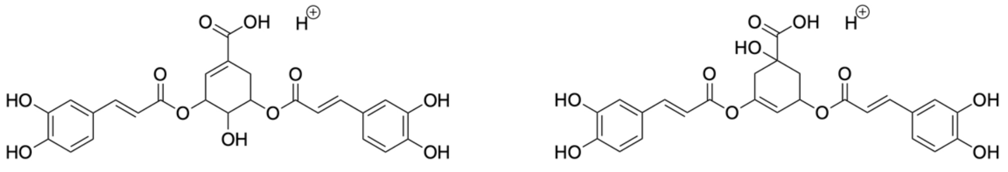

m/z 499

Loss of H_2O + caffeoyl moiety ($C_9H_6O_3$)

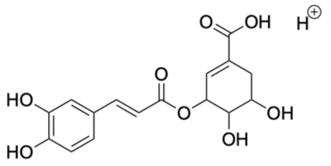

m/z 337

Loss of caffeoylquinic acid moiety ($C_{16}H_{18}O_9$)

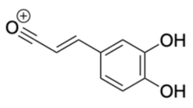

m/z 163

Figure S15: MS^2 spectrum, protonated molecule and fragments of metabolite **22**, annotated as di-*O*-caffeoylquinic acid in level 2 of confidence. *Positions of the caffeoyl groups could not be assigned with confidence and were represented only for illustrating purposes.

Metabolite 23: *O*-caffeoylquinic acid methyl ester

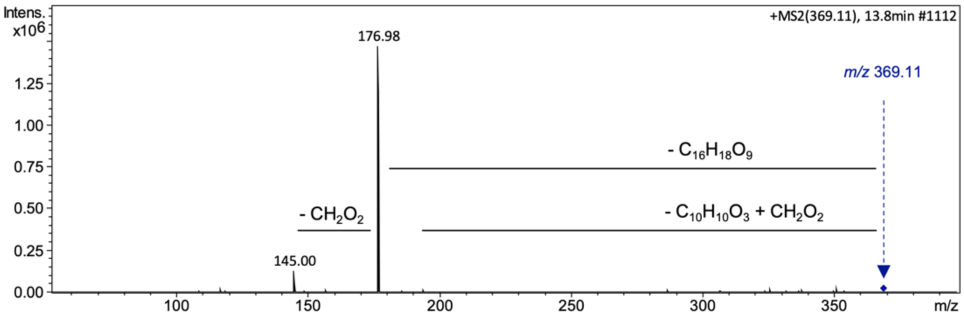

Protonated molecule

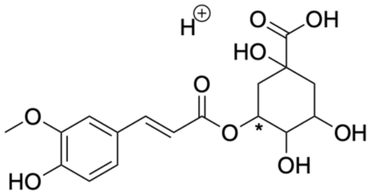

m/z 369.1180

Loss of quinic acid moiety ($C_7H_{12}O_6$)

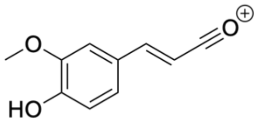

m/z 177

Loss of caffeoyl moiety ($C_{10}H_{10}O_3$)

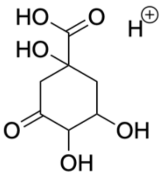

m/z 191

Loss of caffeoyl moiety ($C_{10}H_{10}O_3$) followed by CH_2O_2

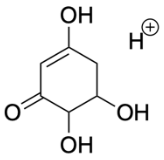

m/z 145

Figure S16: MS^2 spectrum, protonated molecule and fragments of metabolite **23**, annotated as *O*-caffeoylquinic acid methyl ester in level 2 of confidence. *Positions of the caffeoyl group could not be assigned with confidence and were represented only for illustrating purposes.

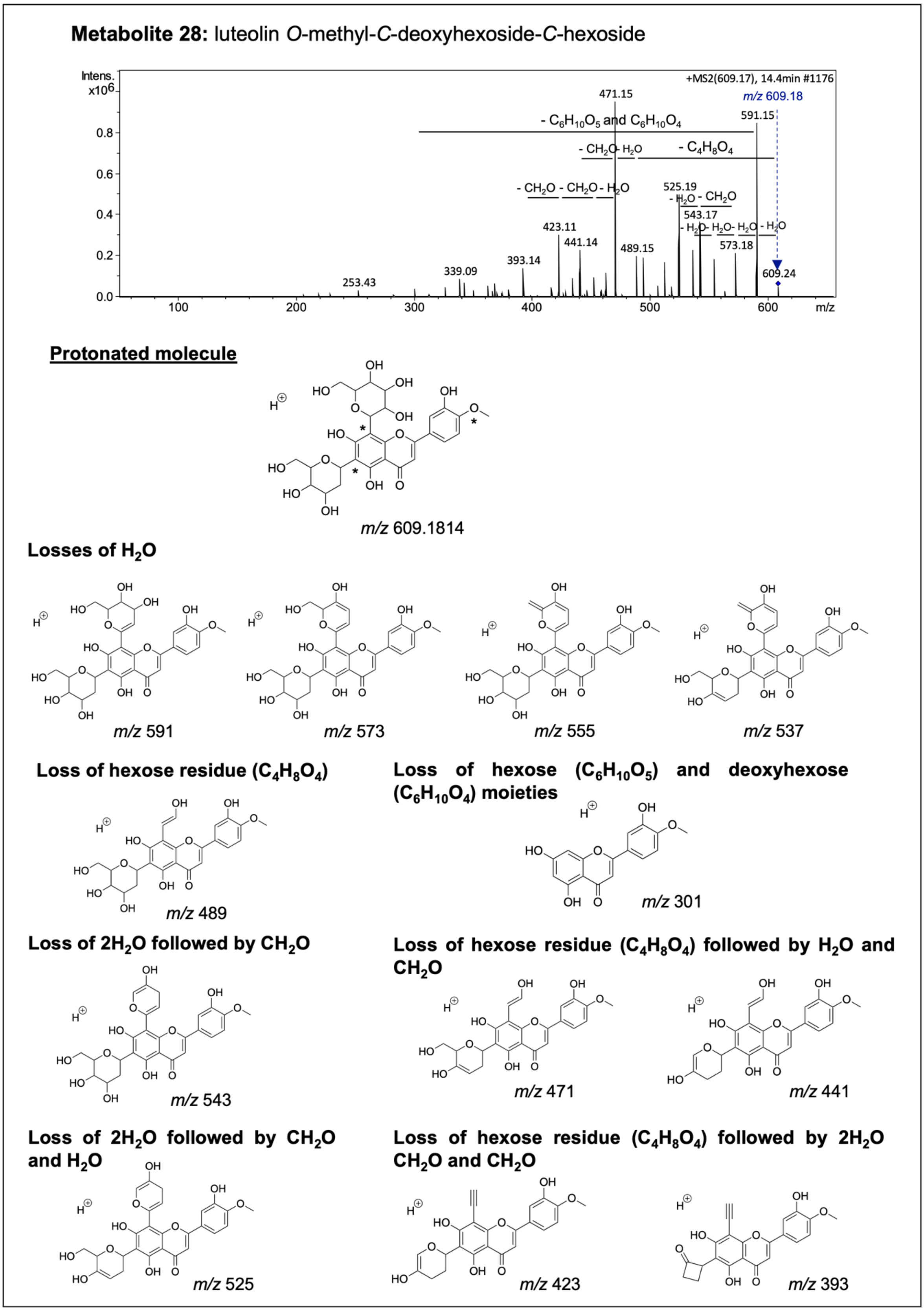

Figure S17: MS[2] spectrum, protonated molecule and fragments of metabolite **28**, annotated as luteolin *O*-methyl-*C*-deoxyhexoside-*C*-hexoside in level 2 of confidence. *Positions of the sugar moieties and OCH_3 group could not be assigned with confidence and were represented only for illustrating purposes.

Metabolite 29: luteolin *C*-hexoside

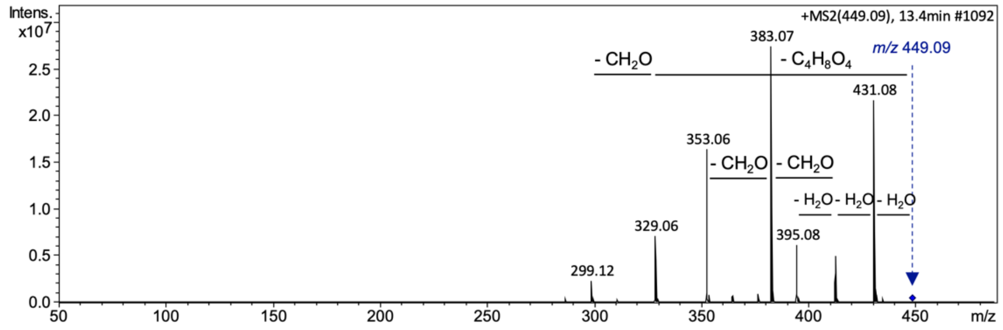

Protonated molecule

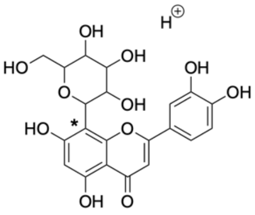

m/z 449.1078

Losses of H_2O

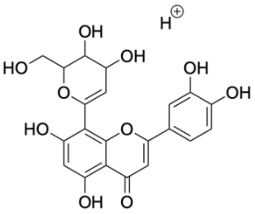

m/z 431

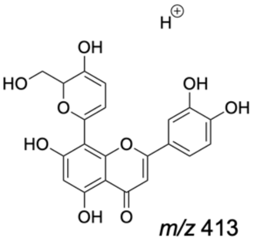

m/z 413

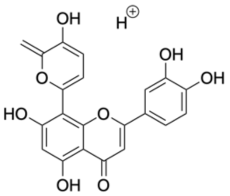

m/z 395

Loss of $2H_2O$ followed by CH_2O

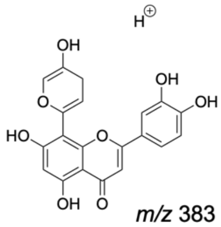

m/z 383

Loss of hexose residue ($C_4H_8O_4$)

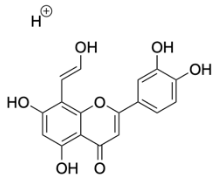

m/z 329

Loss of $2H_2O$ followed by $2CH_2O$

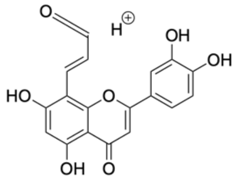

m/z 353

Loss of hexose residue ($C_4H_8O_4$) followed by CH_2O

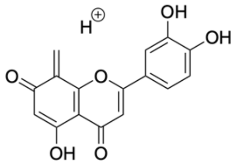

m/z 299

Figure S18: MS^2 spectrum, protonated molecule and fragments of metabolite **29**, annotated as luteolin *C*-hexoside in level 2 of confidence. *Positions of the sugar moieties could not be assigned with confidence and were represented only for illustrating purposes.

Metabolite 32: apigenin *O*-methyl-*C*-hexose-deoxyhexoside

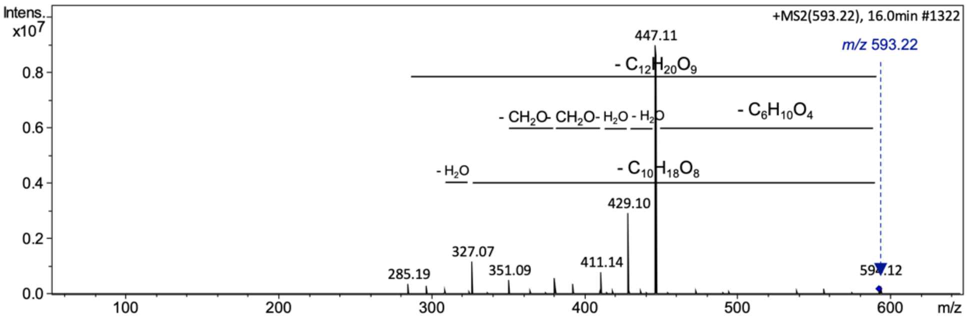

Protonated molecule

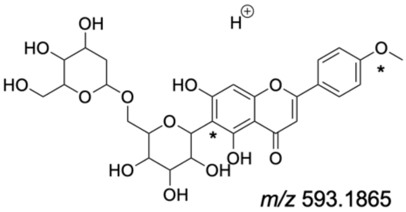

m/z 593.1865

Loss of deoxyhexose moiety ($C_6H_{10}O_4$)

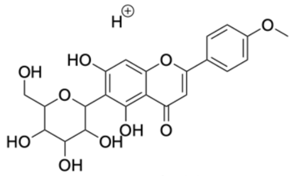

m/z 447

Loss of hexose-deoxyhexose moiety ($C_{12}H_{20}O_9$)

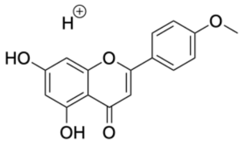

m/z 285

Loss of deoxyhexose moiety ($C_6H_{10}O_4$) followed by losses of H_2O

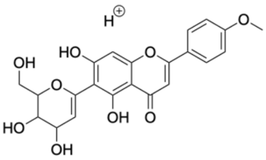

m/z 429

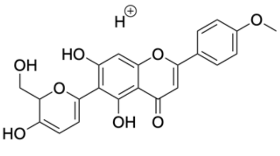

m/z 411

Loss of hexose-deoxyhexose residue ($C_{10}H_{18}O_8$)

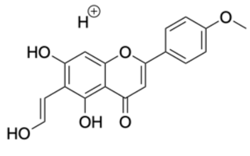

m/z 327

Loss of deoxyhexose moiety ($C_6H_{10}O_4$) followed by loss of $2H_2O$ and losses of CH_2O

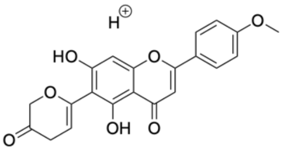

m/z 381

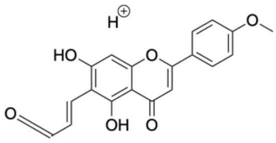

m/z 351

Loss of hexose-deoxyhexose residue ($C_{10}H_{18}O_8$) followed by H_2O

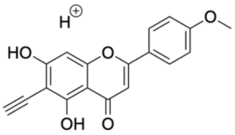

m/z 309

Figure S19: MS^2 spectrum, protonated molecule and fragments of metabolite **32**, annotated as apigenin *O*-methyl-*C*-hexose-deoxyhexoside in level 2 of confidence. *Positions of the sugar moieties and OCH_3 group could not be assigned with confidence and were represented only for illustrating purposes.

Metabolite 34: luteolin *O*-methyl-*O*-deoxyhexoside-*C*-hexoside

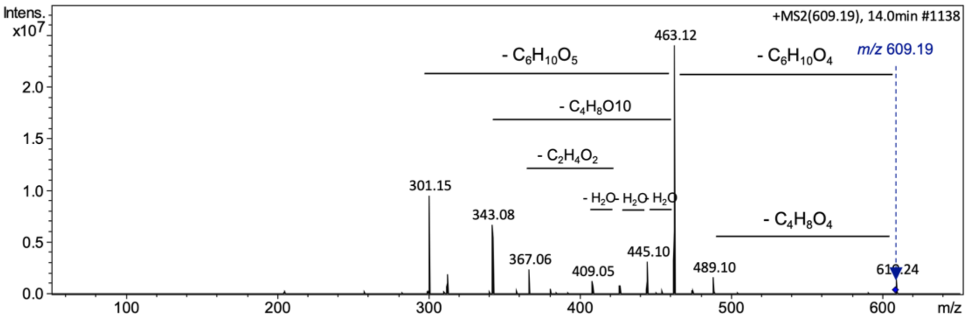

Protonated molecule

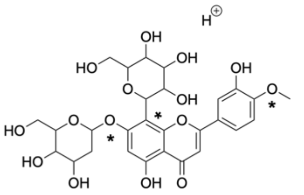

m/z 609.1814

Loss of deoxyhexose moiety ($C_6H_{10}O_4$)

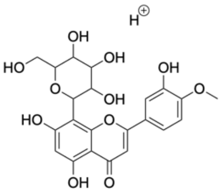

m/z 463

Loss of deoxyhexose moiety ($C_6H_{10}O_4$) and hexoside moiety ($C_6H_{10}O_5$)

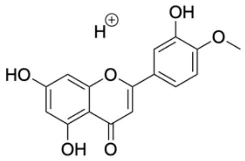

m/z 301

Loss of deoxyhexose moiety ($C_6H_{10}O_4$) followed by losses of H_2O

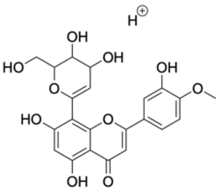

m/z 445

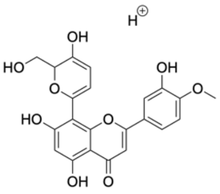

m/z 427

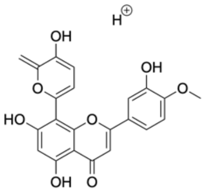

m/z 409

Loss of hexose residue ($C_4H_8O_4$)

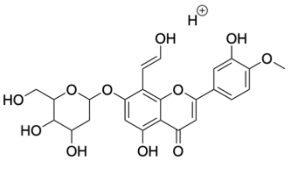

m/z 489

Loss of deoxyhexose moiety ($C_6H_{10}O_4$) followed by loss of hexose residue ($C_4H_8O_4$)

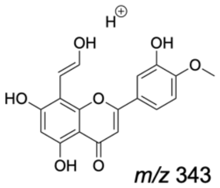

m/z 343

Loss of deoxyhexose moiety ($C_6H_{10}O_4$) followed by loss of $2H_2O$ and $C_2H_4O_2$

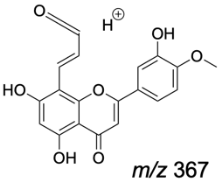

m/z 367

Figure S20: MS[2] spectrum, protonated molecule and fragments of metabolite **34**, annotated as luteolin *O*-methyl-*O*-deoxyhexoside-*C*-hexoside in level 2 of confidence. *Positions of the sugar moieties and OCH_3 group could not be assigned with confidence and were represented only for illustrating purposes.

Metabolite 36: apigenin *O*-methyl-*C*-hexoside

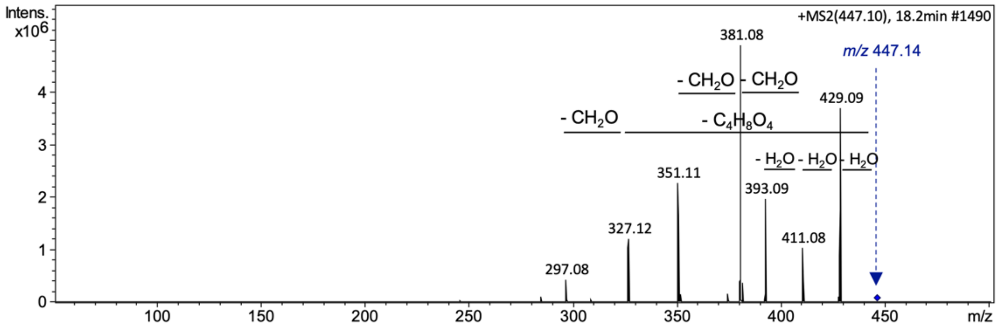

Protonated molecule

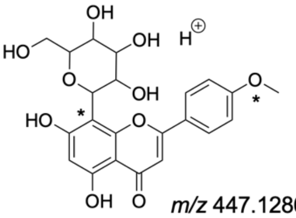

m/z 447.1286

Losses of H_2O

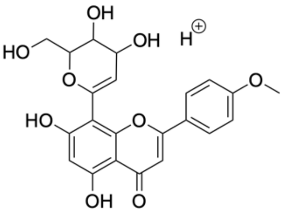

m/z 429

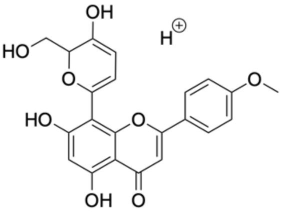

m/z 411

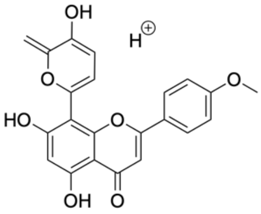

m/z 393

Loss of $2H_2O$ followed by CH_2O

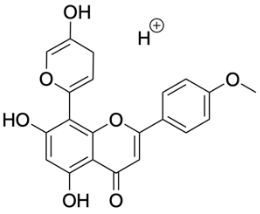

m/z 381

Loss of $2H_2O$ followed by $2CH_2O$

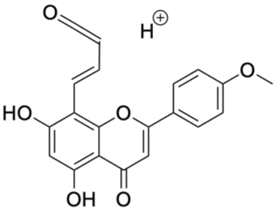

m/z 351

Loss of hexose residue ($C_4H_8O_4$)

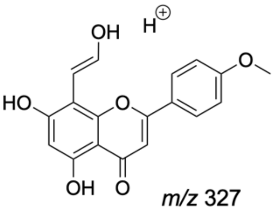

m/z 327

Loss of hexose residue ($C_4H_8O_4$) followed by CH_2O

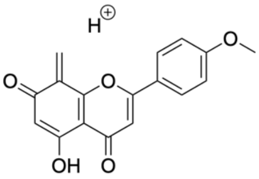

m/z 297

Figure S21: MS^2 spectrum, protonated molecule and fragments of metabolite **36**, annotated as apigenin-*O*-methyl-*C*-hexoside in level 2 of confidence. *Positions of the sugar moieties and OCH_3 group could not be assigned with confidence and were represented only for illustrating purposes.

Metabolite 38: apigenin *O*-methyl-*C*-hexoside

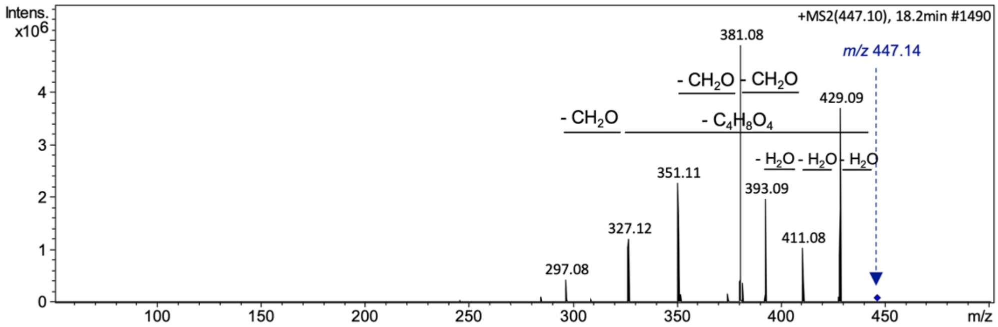

Protonated molecule

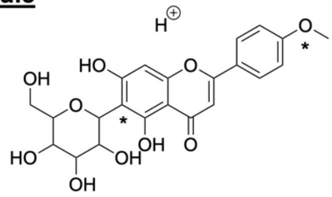

m/z 447.1286

Losses of H_2O

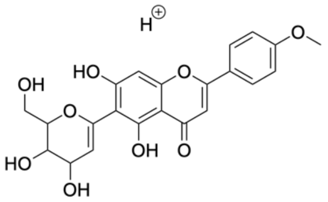

m/z 429

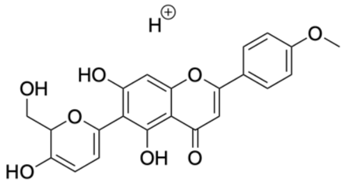

m/z 411

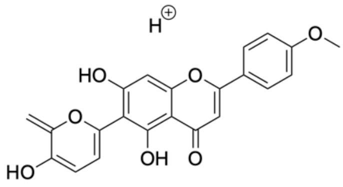

m/z 393

Loss of $2H_2O$ followed by CH_2O

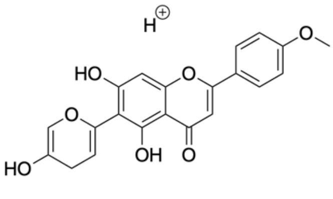

m/z 381

Loss of $2H_2O$ followed by $2CH_2O$

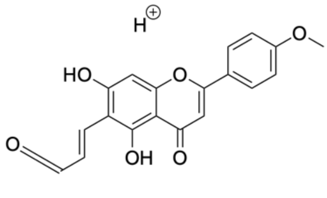

m/z 351

Loss of hexose residue ($C_4H_8O_4$)

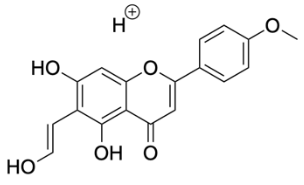

m/z 327

Loss of hexose residue ($C_4H_8O_4$) followed by CH_2O

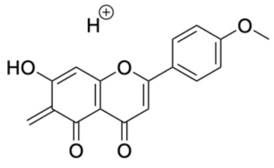

m/z 297

Figure S22: MS^2 spectrum, protonated molecule and fragments of metabolite **38**, annotated as apigenin-*O*-methyl-*C*-hexoside in level 2 of confidence. *Positions of the sugar moieties and OCH_3 group could not be assigned with confidence and were represented only for illustrating purposes.

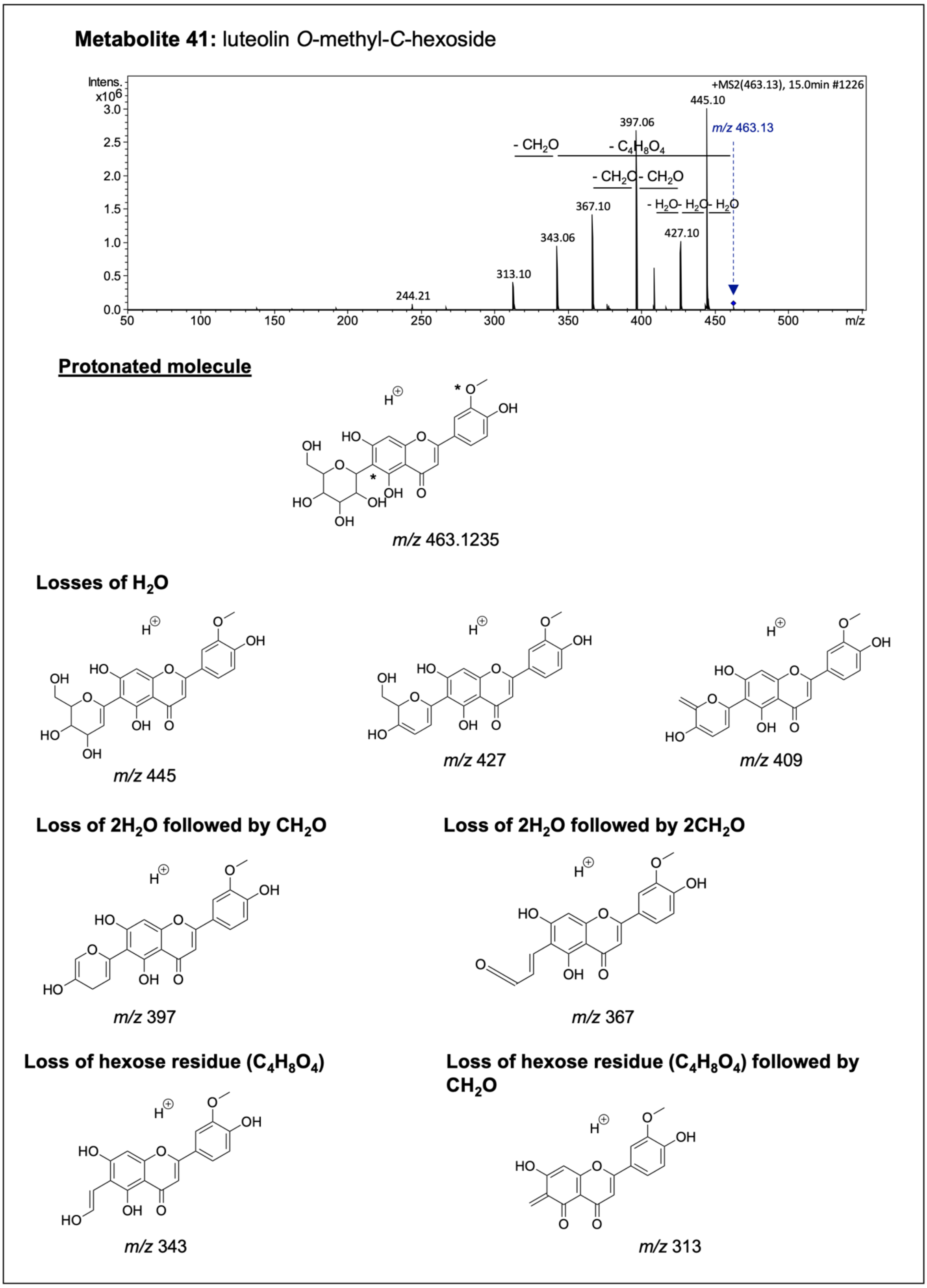

Figure S23: MS^2 spectrum, protonated molecule and fragments of metabolite **41**, annotated as luteolin-*O*-methyl-*C*-hexoside in level 2 of confidence. *Positions of the sugar moieties and OCH_3 group could not be assigned with confidence and were represented only for illustrating purposes.

Metabolite 44: apigenin *C*-hexose-hexoside

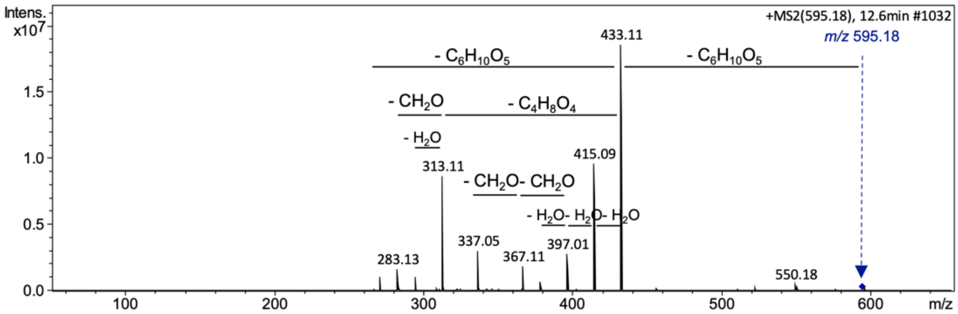

Protonated molecule

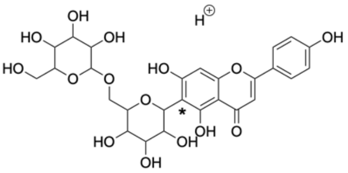

m/z 595.1657

Loss of hexose moiety ($C_6H_{10}O_5$)

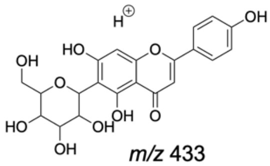

m/z 433

Loss of hexose-hexose moiety ($2C_6H_{12}O_5$)

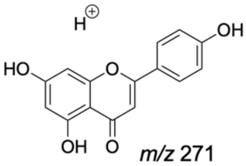

m/z 271

Losses hexose moiety ($C_6H_{10}O_5$) followed by losses of H_2O

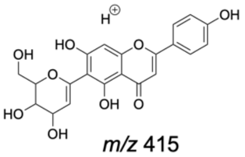

m/z 415

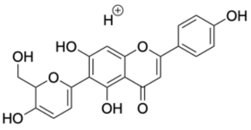

m/z 397

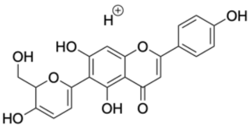

m/z 379

Loss of hexose moiety ($C_6H_{10}O_5$) and hexose residue ($C_4H_8O_4$)

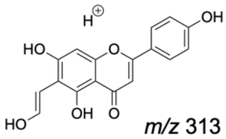

m/z 313

Loss of $2H_2O$ followed by CH_2O

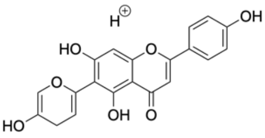

m/z 367

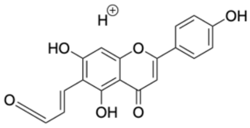

m/z 337

Loss of hexose residue ($C_4H_8O_4$) followed by H_2O

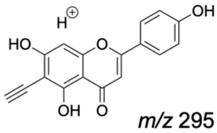

m/z 295

Loss of hexose residue ($C_4H_8O_4$) followed by CH_2O

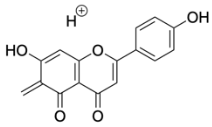

m/z 283

Figure S24: MS^2 spectrum, protonated molecule and fragments of metabolite **44**, annotated as apigenin *C*-hexose-hexoside in level 2 of confidence. *Positions of the sugar moieties could not be assigned with confidence and were represented only for illustrating purposes.

Metabolite 46: piperaduncin A

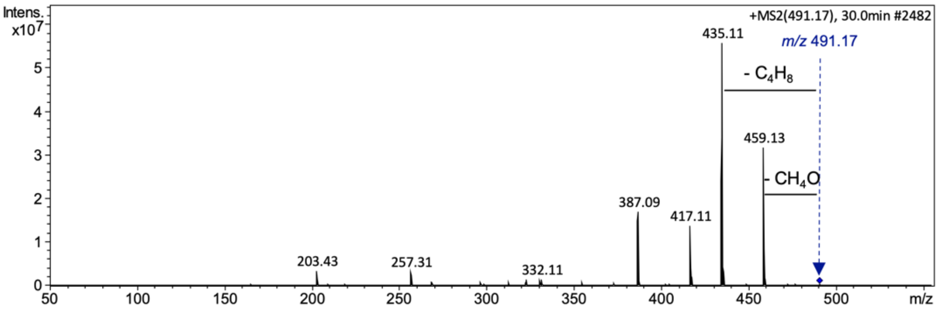

Protonated molecule

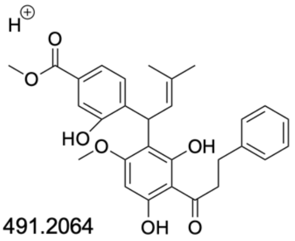

m/z 491.2064

Loss of CH_4O

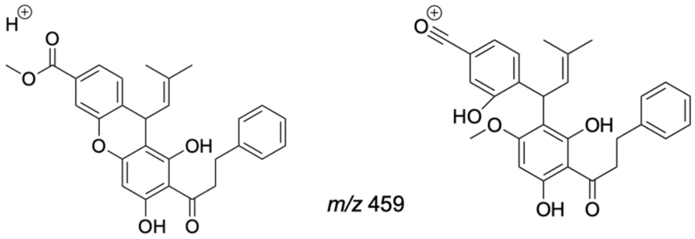

Loss of C_4H_8

m/z 435

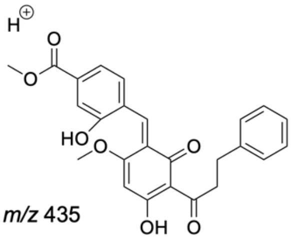

Figure S25: MS^2 spectrum, protonated molecule and fragments of metabolite **46**, annotated as piperaduncin A in level 2 of confidence. *Positions of the sugar moieties could not be assigned with confidence and were represented only for illustrating purposes.

Table S1: Spectrometric and spectroscopic data obtained by LC-UV(DAD)-MS (Ion trap) in the positive ionization mode for the 25 most important discriminant metabolites of *Vernonathura polyanthes* individuals growing in areas affected by the Fundão dam collapse and in unaffected areas along with the indication of putative classes, putative metabolites and molecular formulae within a certain confidence level.

Peak ID	Node ID	VIP	Rt	*m/z*	Precursor ion → Fragments	λ_{max} (nm)	Characteristic neutral loss	Putative Class and Subclass	Putative metabolite and Molecular Formula	Confidence level
**Affected areas**										
**1**	736V	2.41	22.1	411.09 [M+H]	411 → 393, <u>375</u>, **341**, **307**, 271, 247, **212**, 181, 163 bp, 145, 117	222	H_2S: 34 Da (375 → 341) (341 → 307) $C_5H_7NO_3$: 129 Da (341 → 212)	**Peptide** Glutathione derivative	(Glu-Cys)-Gly-Cys **$C_{13}H_{22}N_4O_7S_2$**	Level 2
**2**	490V	2.33	14.5	624.23 [M+H]	<u>624</u> → 606, 578, **538** bp, **520**, 452, 434, 392	216	$CH_2CO + CO_2$: 86 Da (624 → 538) (520 →434) $C_5H_{10}N_2O_3$: 146 Da (538 → 392)	**Peptide** Glutathione/LysAc derivative	-	Level 3
**3**	1195V	1.98	16.9	509.20 [M+H]	509 → 477 bp, 390	218	-	-	-	Level 4

**4**	432V	1.75	14.8	552.24 [M+H]	552 → **506bp**, **466**, 420, 406, 360, 257, 201, 173	213, 252, 265 sh 345	$C_5H_{10}N_2O_3$: 146 Da (552 → 406) $CH_2CO + CO_2$: 86 Da (552 → 466) CH2O2 or CH_2S: 46 Da (552 → 506)	**Peptide** Glu-Cys/LysAc derivative	(Glu-Cys)-Met-LysAc **$C_{21}H_{37}N_5O_8S_2$**	Level 2
**5**	481V	1.69	17.7	492.22 [M+H]	492 → 474 bp, **428**, 346	219, 300, 320 sh	$C_5H_{10}N_2O_3$: 146 Da (492 → 346) CH_2O_2 or CH_2S: 46 Da (474 → 428)	**Peptide** Glu-Cys derivative	(Glu-Cys)-Leu-Gln **$C_{19}H_{33}N_5O_8S$**	Level 2
**6**	648V	1.62	17.4	460.20* [Frag]	478 → 460 bp, **414**, 392, **374**, 364, 346 460→ 442, 428, 414 bp, 387, 374, 356, 346, 328, 312, 288, 241, 215, 189, 171, 143	219, 300 sh, 330	$C_2H_2O + CO_2$: 86 Da (478 → 392) (460 → 374) CH_2S: 46 Da (460→ 414) (392 → 346)	**Peptide** Glutathione/LysAc derivative	(Glu-Cys)-Gly-LysAc **$C_{18}H_{31}N_5O_8S$**	Level 2
**7**	820V	1.57	19.4	528.23 [M+H]	528 → 510 bp, 496, **464**, **442**, 424, 406, 396, 241	221	$CH_2CO + CO_2$: 86 Da (528 → 442) (510 → 424) HCOOH: 46 Da (510 → 464)	**Peptide** LysAc derivative	-	Level 3

**8**	1384V	1.56	13.9	654.23 [M+H]	654 → 636, **608**, 594, **568**, 508 bp, 490, 480, 464, 446, 434, 408, 374, 328, 292, 243	216, 293 sh, 332	$C_5H_{10}N_2O_3$: 146 Da (654 → 508)<br>$CH_2CO + CO_2$: 86 Da (654 → 568)<br>HCOOH: 46 Da (654 → 608)<br>CH_2CO: 42 Da (636 →594)	**Peptide**<br>Glutathione/LysAc/N-acetyl derivative	-	Level 3
**9**	258V	1.55	14.6	580.23 [M+H]	580 → 562, 538, **534**, 520, 502, **494**, 448, 434 bp, 374, 328, 292, 243	217, 289, 229	$CH_2CO + CO_2$: 86 Da (580 → 494)<br>HCOOH: 46 Da (580 → 534)<br>CH2CO: 42 Da (562 → 520)	**Peptide**<br>LysAc/N-acetyl derivative	-	Level 3
**10**	1002V	1.46	22.9	351.18 [M+H]	351 → 333, 319, **309**, 291, **273 bp**, 259, 241, 231, 213, 203, 169	222	CH_2CO: 42 Da (551 → 309)<br>HCOOH: 46 Da (319 → 273)	**Peptide**<br>N-acetyl derivative	-	Level 3
**11**	984V	1.40	19.3	534.23 [M+H]	534 → 516, **492**, **488**, 474 bp, **388,** 370, 328, 310, 292, 268, 241, 199, 173	221	$C_5H_{10}N_2O_3$: 146 Da (534 → 388)<br>CH2CO: 42 Da (534 → 492)<br>CH_2O_2 or CH_2S: 46 Da (534 → 488)<br>$CH_2CO + CO_2$: 86 Da (474 → 388)	**Peptide**<br>Glu-Cys/LysAc/N-acetyl derivative	(Glu-Cys)-Leu-LysAc<br>**$C_{22}H_{39}N_5O_8S$**	Level 2

**12**	766V	1.36	20.4	403.14 [M+H]	403 → 317	220	$CH_2CO + CO_2$: 86 Da (403 → 317)	**Peptide** LysAc derivative	Leu-Thr-LysAc **$C_{18}H_{34}N_4O_6$**	Level 2
**13**	1367V	1.34	23.6	417.14 [M+H]	417 → 385 bp, **331**, 309, 291, 249, 161, 143	222	$CH_2CO + CO_2$: 86 Da (417 → 331)	**Peptide** LysAc derivative	Leu-Asp-LysAc **$C_{18}H_{32}N_4O_7$**	Level 2
**Unaffected areas**										
**k14**	26V	2.72	20.0	535.23 [M+H]	535 → 517, 499, 443, 391, 325, 319, 289, 211, 193, 181, 163 bp	221, 298 sh, 326		**Phenolic** Phenylpropanoid	di-*O*-caffeoylquinic acid derivative **$C_{25}H_{26}O_{13}$**	Level 2
**15**	54V	2.68	21.1	411.08* [Frag]	621 → 603, 559, 411 bp, 393 411 → 393, **307**, 271, 247, 213, 181, 163 bp, 145, 135	221	$CH_2CO + CO_2$: 86 Da (393 → 307) - 104	**Peptide** LysAc derivative	-	Level 3
**16**	303V	2.28	10.9	341.08 [M+H]	341 → 179, 163 bp, 145	211, 299 sh, 325	$C_5H_{10}N_2O_3S$: 178 Da (341 → 163)	**Peptide/Phenolic** Gly-Cys/Phenylpropanoid derivative	(Cys-Gly)-caffeoyl **$C_{14}H_{16}N_2O_6S$**	Level 2
**17**	447V	2.03	17.0	343.21 [M+H]	343 → 326, 280, 214 bp, 179, 161	217, 299 sh, 327	$C_5H_7NO_3$: 129 Da (343 → 214) HCOOH: 46 Da (326 → 280)	**Peptide/Phenolic** Glu-Cys/ Phenylpropanoid derivative	-	Level 3
**18**	456V	1.88	12.0	343.07 [M+H]	343 → 325 bp, 297	200, 232 sh, 284	-	-	-	Level 4

**19**	125V	1.82	20.4	535.22 [M+H]	535 → 517, 499, 373, 325 bp, 307, 211, 193, 181, 163	220, 299 sh, 328		**Phenolic** Phenylpropanoid	di-*O*-caffeoylquinic acid derivative **$C_{25}H_{26}O_{13}$**	Level 2
**20**	436V	1.80	17.7	487.12 [M+H]	487 → 469, 442, 353, **307**, 163 bp, 145	218, 298 sh, 325	HCOOH: 46 Da (353 → 307)	**Peptide/Phenolic** Phenylpropanoid derivative	-	Level 3
**21**	458V	1.71	17.1	451.18 [M+H]	451 → 433, 415, 379, 346, 271, 253 bp, 235, 217, 189, 163, 143	216, 298 sh, 326		**Phenolic** Phenylpropanoid	-	Level 3
**22**	617V	1.57	13.6	517.13 [M+H]	517 → 499 bp, 379, 337, 163	202, 210 sh, 293 sh, 327		**Phenolic** Phenylpropanoid	di-*O*-caffeoylquinic acid **$C_{25}H_{24}O_{12}$**	Level 2
**23**	154V	1.46	13.9	369.11 [M+H]	369 → 177 bp, 145	202, 214 sh, 298 sh, 326		**Phenolic** Phenylpropanoid	*O*-caffeoylquinic acid methyl ester **$C_{17}H_{20}O_9$**	Level 2
**24**	622V	1.36	17.8	596.21 [M+H]	596 → 565, 525, **457**, 373, 325, 295, 277 bp, **231**, 167	218, 298 sh, 325	CH_3NH_2: 31 Da (596 → 565) C_7H_8O: 108 Da (565 → 457) HCOOH: 46 Da (277 → 231)	**Peptide** Aromatic N-methyl/tyrosyl derivative	-	Level 3
**25**	612V	1.34	13.0	380.22 [M+H]	380 → 362 bp, 344	215, 295 sh, 330	-	-	-	Level 4

Pead ID, identification of the peak; Node ID, identification of the node; VIP, value of the variable importance in projection for the corresponding feature; R_t, retention time in minutes; *m/z* $[M+H]^+$, mass-to-charge ration of the protonated molecule; λ_{max}, maximum absorbance in the ultraviolet spectra in nanometers; * indicates a fragment ion.

Table S2: - obtained by LC-UV(DAD)-MS (Ion trap) in the positive ionization mode for the 25 most important discriminant metabolites of *Piper aduncum* individuals growing in areas affected by the Fundão dam collapse and unaffected areas along with the indication of putative classes, putative metabolites and molecular formulae within a certain confidence level.

Peak ID	Node ID	VIP	Rt	*m/z*	Precursor ion → Fragments	λ_{max} (nm)	Putative Class	Putative Class and Subclass	Putative Metabolite and Molecular Formula	Confidence level
**Affected areas**										
26	185P	2.39	44.0	607.31 $[M+H]^+$	607 → 579, 575, 547 bp, 473	227, 408, 665	CH_3OH: 32 Da (607 → 575) (575 → 547)	-	-	Level 4
27	233P	2.36	39.7	609.29 $[M+H]^+$	609 → 591 bp, 577, 559, 541, 531, 515	226	-	-	-	Level 4
28	851P	2.22	14.5	609.18 $[M+H]^+$	609 → 591, 573, 555, 543, 537, 525, 489, 471 bp, 441, 423, 393, 339, 301, 253	214, 259 sh, 270, 341	$C_4H_8O_4$: 120 Da (571 → 471) $C_6H_{10}O_5$ + $C_6H_{10}O_4$: 308 Da (609 → 301) CH_2O: 30 Da (573 →543) (555 → 525) (471 → 441) (423 → 393)	**Phenolic** Flavonoid	luteolin *O*-methyl-*C*-deoxyhexose-*C*-hexoside **$C_{28}H_{32}O_{15}$**	Level 2
29	1240P	1.96	13.4	449.105 $[M+H]^+$	449 → 431, 413, 395, 383 bp, 353, 329, 299	205, 220 sh, 256 sh, 269, 347	$C_4H_8O_4$: 120 Da (449 → 329) CH_2O: 30 Da (413 →383) (383 → 353) (329 → 299)	**Phenolic** Flavonoid	luteolin *C*-hexoside **$C_{21}H_{20}O_{11}$**	Level 2

30	1245P	1.90	35.5	531.24 [M+H]+	531 → 513, 475 bp, 457, 381, 325, 299, 273, 217	217, 265 sh, 286	C_4H_8: 56 Da (531 → 475) (381 → 325) (273 → 217)	**Phenolic/Terpenoid** Prenylated flavonoid	-	Level 3
31	1241P	1.89	30.3	507.20 [M+H]+	507 → 493, 475, 451 bp, 433, 419, 401, 359, 327, 303, 207	221, 264 sh, 286	C_4H_8: 56 Da (507 → 451) (359 → 303) CH_3OH: 32 Da (507 → 475) (433 → 401)	**Phenolic/Terpenoid** Prenylated flavonoid	-	Level 3
32	1637P	1.88	16.0	593.19 [M+H]+	593 → 447 bp, 429, 411, 381, 351, 327, 309, 285	214, 269, 329	$C_6H_{10}O_4$: 146 Da (593 → 447) $C_6H_{10}O_5$: 162 Da (447 → 285) $C_6H_{10}O_5$ + $C_6H_{10}O_4$: 308 Da (593 → 285) CH_2O: 30 Da (411 →381) (381 → 351) (329 → 299)	**Phenolic** Flavonoid	apigenin *O*-methyl-*C*-hexose-deoxyhexoside **$C_{28}H_{32}O_{14}$**	Level 2
33	1249P	1.84	38.7	545.26 [M+H]+	545 → 531, 513, 489 bp, 475, 457, 325, 287, 231	226	C_4H_8: 56 Da (545 → 489) CH_3OH: 32 Da (545 → 513) (487 → 457)	**Terpenoid** Prenylated derivate	-	Level 3

34	1255P	1.83	14.0	609.18 [M+H]$^+$	609 → 489, 463 bp, 445, 409, 367, 343, 301	202, 257, 267 sh, 351	$C_6H_{10}O_4$: 146 Da (609 → 463) (489 → 343) $C_4H_8O_4$: 120 Da (609 → 489) (463 → 343) $C_6H_{10}O_5$ + $C_6H_{10}O_4$: 308 Da (609 → 301)	**Phenolic** Flavonoid	luteolin *O*-methyl-*O*-deoxyhexoside-*C*-hexoside **$C_{28}H_{32}O_{15}$**	Level 2
35	1754P	1.82	17.6	459.20 [M+H]$^+$	459 → 441, 423, 405, 397, 379, 319, 231, 211 bp, 193, 175, 135	218, 270, 335	-	-	-	Level 4
36	1331P	1.76	18.1	447.13 [M+H]$^+$	447 → 429, 411, 393, 381 bp, 351, 327, 297	215, 269, 335	$C_4H_8O_4$: 120 Da (447 → 327) CH_2O: 30 Da (411 →381) (381 → 351) (327 → 297)	**Phenolic** Flavonoid	apigenin *O*-methyl-*C*-hexoside **$C_{22}H_{22}O_{10}$**	Level 2
37	127P	1.76	42.7	623.29 [M+H]$^+$	623 → 605 bp, 573, 545	226	-	-	-	Level 4
38	1269P	1.69	14.9	447.13 [M+H]$^+$	447 → 429 bp, 411, 393, 381, 351, 327, 297	214, 271, 285 sh, 336	$C_4H_8O_4$: 120 Da (447 → 327) CH_2O: 30 Da (411 →381) (381 → 351) (327 → 297)	**Phenolic** Flavonoid	apigenin *O*-methyl-*C*-hexoside **$C_{22}H_{22}O_{10}$**	Level 2

39	1368P	1.65	32.2	529.23 [M+H]$^+$	529 → 511, 473 bp, 455, 425, 351, 323, 269, 217	224	C_4H_8: 56 Da (529 → 473)	**Terpenoid** Prenylated derivative	-	Level 3
40	2047P	1.64	31.9	507.21 [M+H]$^+$	507 → 489 bp, 475, 457, 417, 357, 325	224, 283	-	-	-	Level 4
41	103P	1.55	15.1	463.13 [M+H]$^+$	463 → 445 bp, 427, 409, 397, 367, 343, 313	214, 269 sh, 272, 340	$C_4H_8O_4$: 120 Da (463 → 343) CH_2O: 30 Da (427 →397) (397 → 367) (343 → 313)	**Phenolic** Flavonoid	luteolin *O*-methyl-*C*-hexoside **$C_{22}H_{22}O_{11}$**	Level 2
42	1276P	1.53	18.0	342.18 [M+NH$_4$]$^+$	342 → 325 bp, 307, 293, 221, 210, 193, 175, 161	216, 270, 325	-	-	-	Level 4
43	1666P	1.52	28.9	463.18 [M+H]$^+$	463 → 445, 407 bp, 389, 313, 271	224	C_4H_8: 56 Da (463 → 407)	**Terpenoid** Prenylated derivative	-	Level 3
**Unaffected areas**										
44	427P	1.91	12.6	595.17 [M+H]$^+$	595 → 433 bp, 415, 397, 379, 367, 337, 313, 295, 283, 271	213, 268, 334	$C_6H_{10}O_5$: 162 Da (595 → 433) (433 → 271) $C_6H_{10}O_5$ + $C_4H_8O_4$: 282 Da (595 → 313) CH_2O: 30 Da (397 →367) (367 → 337) (313 → 283)	**Phenolic** Flavonoid	apigenin *C*-hexose-hexoside **$C_{27}H_{30}O_{15}$**	Level 2

45	515P	1.80	17.7	441.18 [M+H]$^+$	441 → 397 bp, 355, 245	218	-	-	-	Level 4
46	415P	1.64	30.0	491.18 [M+H]$^+$	491 → 459, 435 bp, 417, 387, 297, 257	213, 269 sh, 298, 346	C_4H_8: 56 Da (491 → 435) CH_3OH: 32 Da (491 → 459) CH_2O: 30 Da (417 →387)	**Terpenoid/Phenolic** Prenylated flavonoid	piperaduncin A **$C_{29}H_{30}O_7$**	Level 2
47	477P	1.62	37.3	393.21 [M+NH]$^+$	393 → 376, 337, 323, 297, 289, 283, 269 bp, 233, 165	225	-	-	-	Level 4
48	60P	1.57	43.5	607.31 [M+H]$^+$	607 → 575, 547 bp, 473	227	-	-	-	Level 4
49	195P	1.52	32.8	347.23 [M+H]$^+$	347 → 329, 277, 265 bp, 249, 235, 203, 173	224	-	-	-	Level 4
50	871P	1.37	27.3	489.19 [M+H]$^+$	489 → 475, 457 bp, 433, 415, 385, 353, 337, 233	223	C_4H_8: 56 Da (489 → 433) CH_3OH: 32 Da (489 → 457) (385 → 353) CH_2O: 30 Da (415 →385)	**Terpenoid** Prenylated derivative	-	Level 3

Pead ID, identification of the peak; Node ID, identification of the node; VIP, value of the variable importance in projection for the corresponding feature; R_t, retention time in minutes; *m/z* [M+H]$^+$, mass-to-charge ration of the protonated molecule; λ_{max}, maximum absorbance in the ultraviolet spectra in nanometers; * indicates a fragment ion.

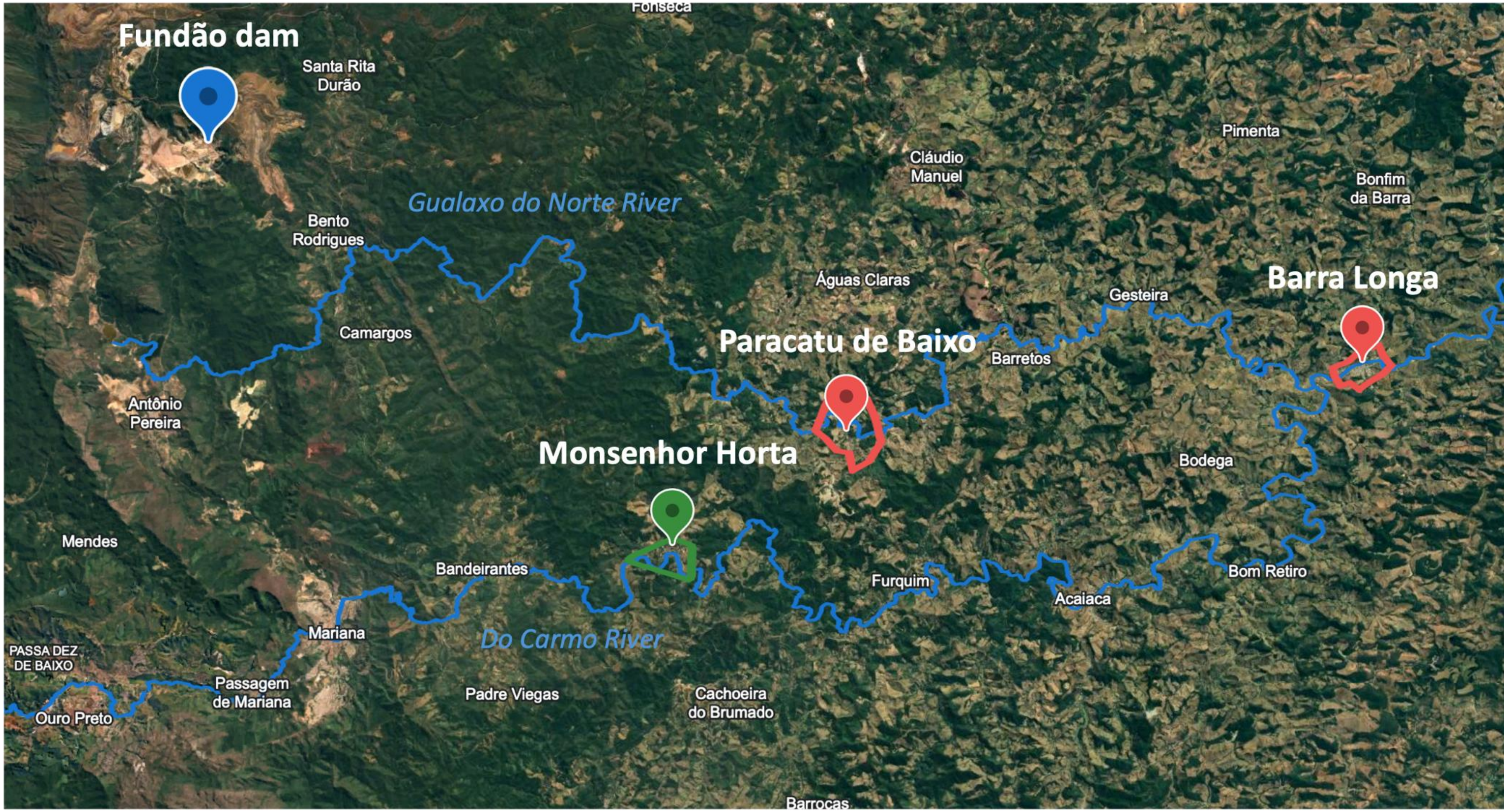

Figure S26. Location of the Fundão dam and where *Vernonanthura polyanthes* and *Piper aduncum* individuals were collected, in areas affected by the Fundão dam collapse (Paracatu de Baixo e Barra Longa, red pins) and in unaffected areas (Monsenhor Horta, green pin), Google Earth Pro.

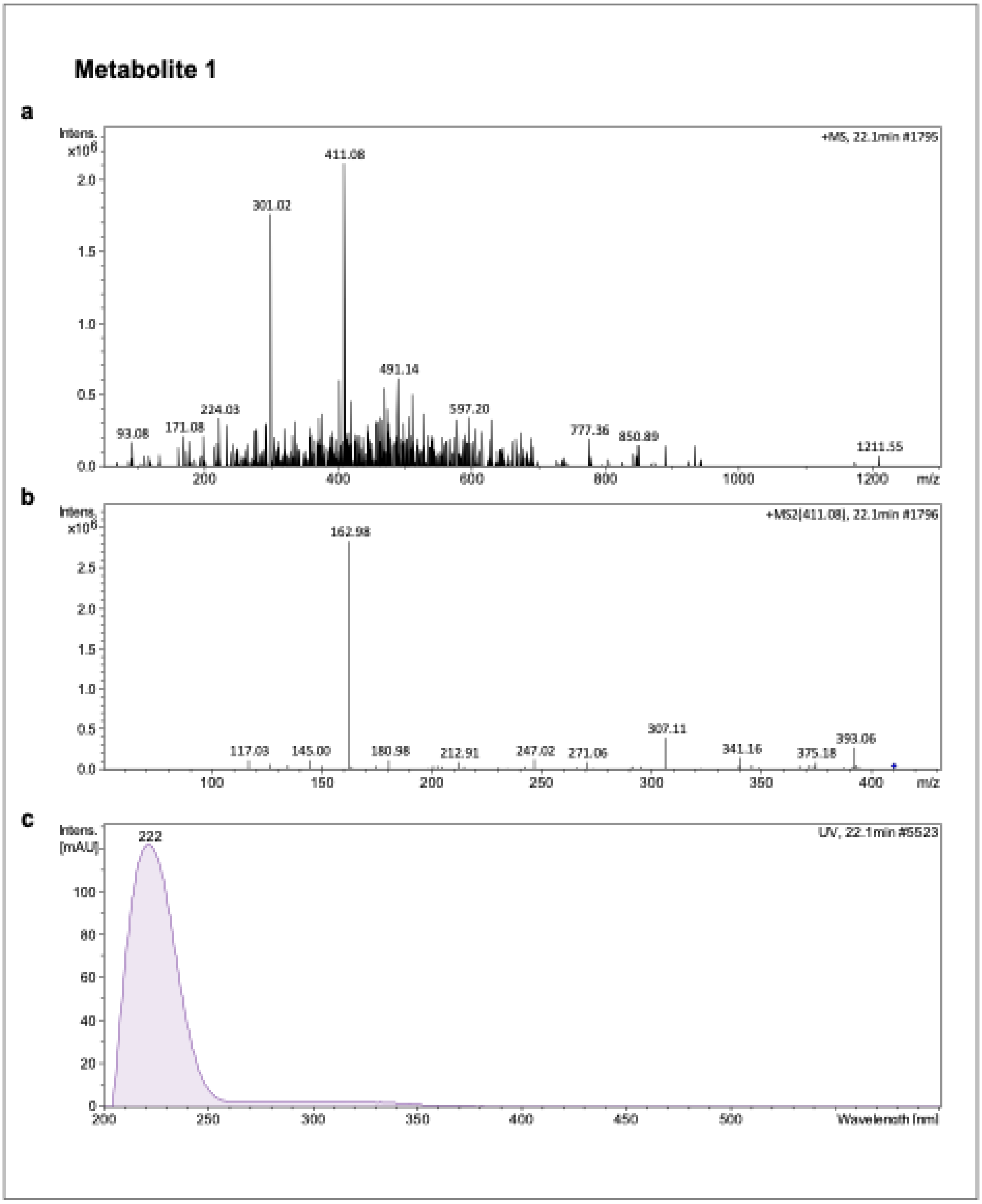

Figure S27. Spectrometric and spectroscopic data of metabolite **1**, indicated as a discriminant compound for *Vernonanthura polyanthes*, including MS spectra (**a**), MS^n spectra (**b**) and UV spectra (**c**).

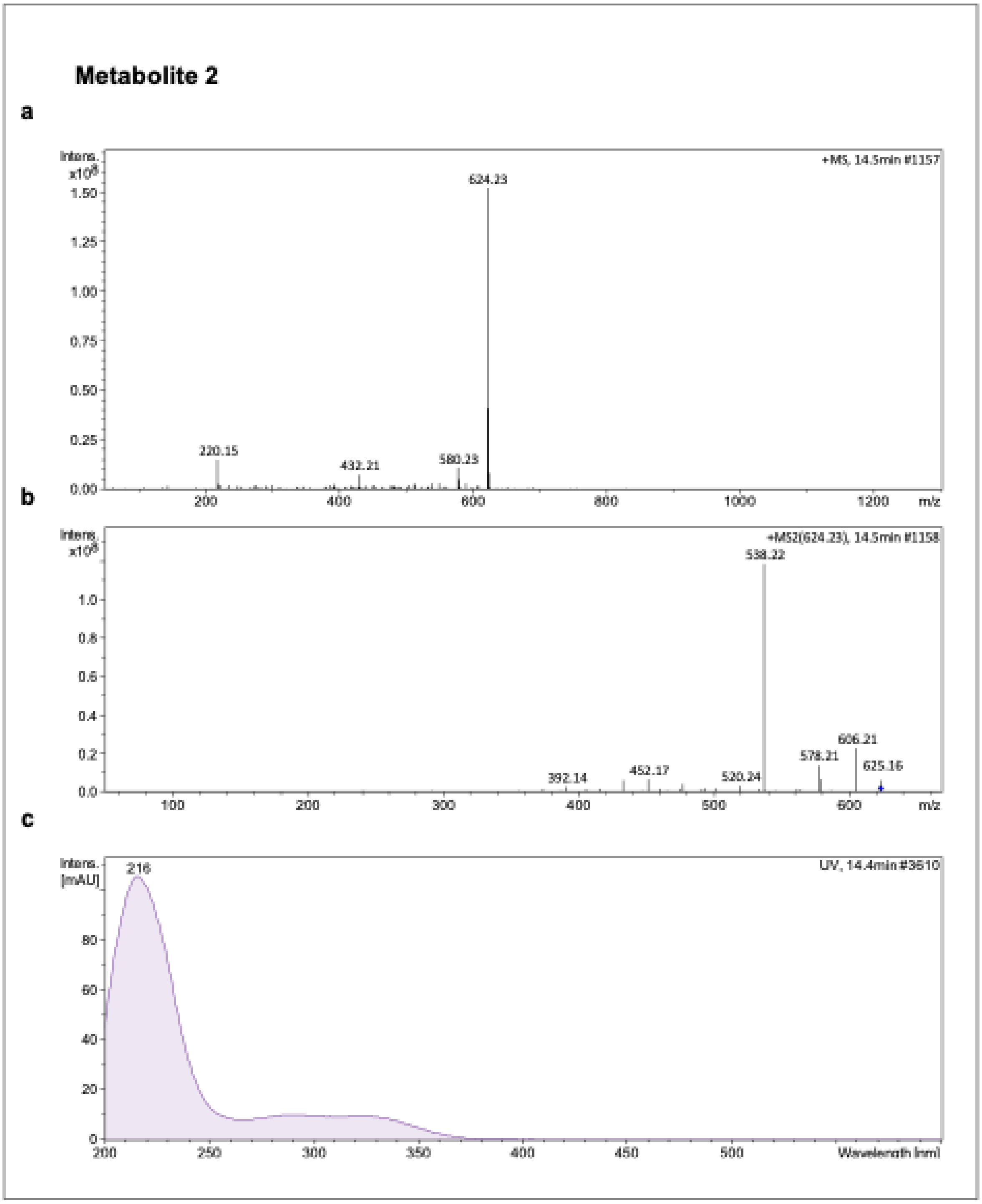

Figure S28. Spectrometric and spectroscopic data of metabolite **2**, indicated as a discriminant compound for *Vernonanthura polyanthes*, including MS spectra (**a**), MS^n spectra (**b**) and UV spectra (**c**).

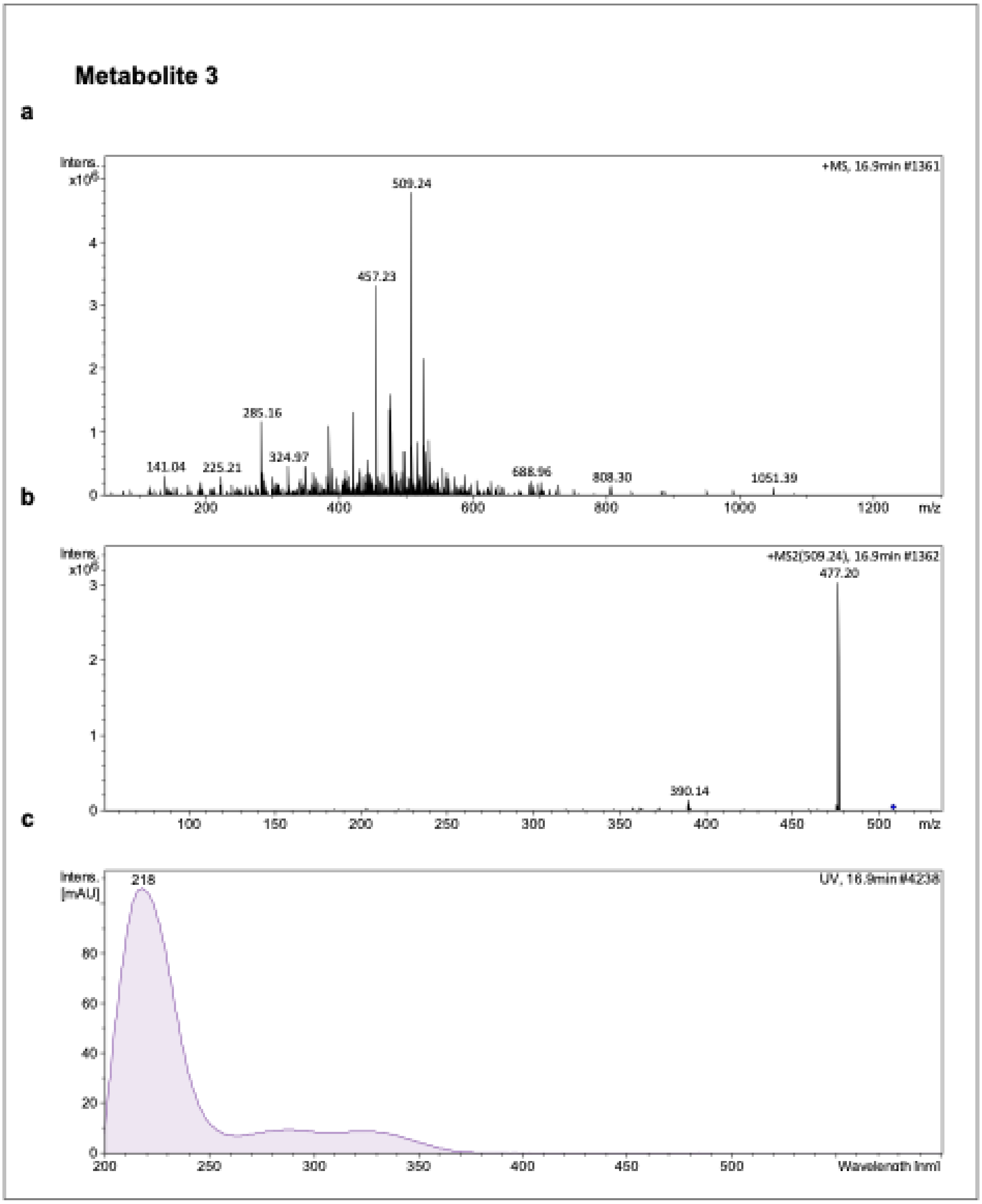

Figure S29. Spectrometric and spectroscopic data of metabolite **3**, indicated as a discriminant compound for *Vernonanthura polyanthes*, including MS spectra (**a**), MS^n spectra (**b**) and UV spectra (**c**).

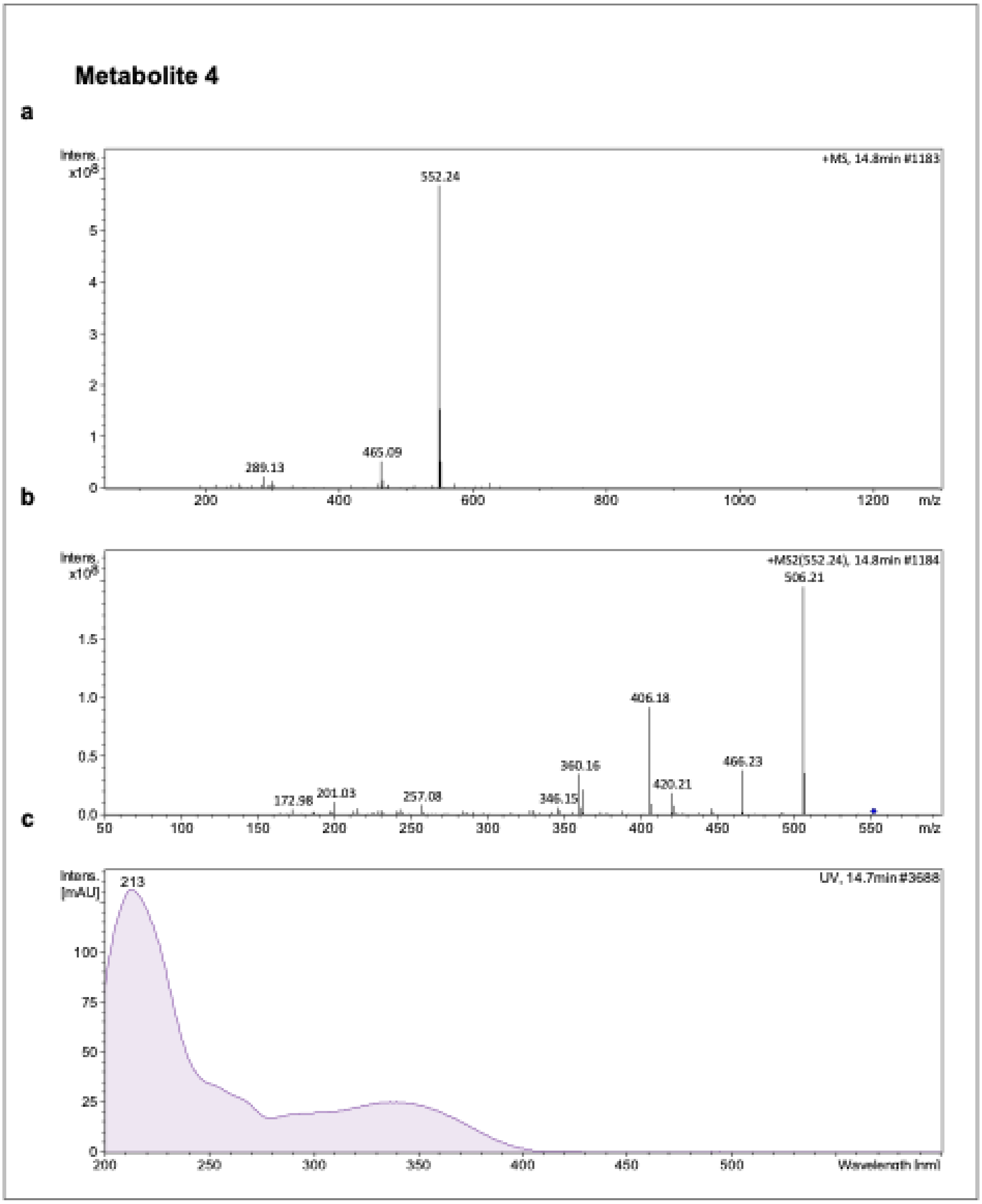

Figure S30. Spectrometric and spectroscopic data of metabolite **4**, indicated as a discriminant compound for *Vernonanthura polyanthes*, including MS spectra (**a**), MS^n spectra (**b**) and UV spectra (**c**).

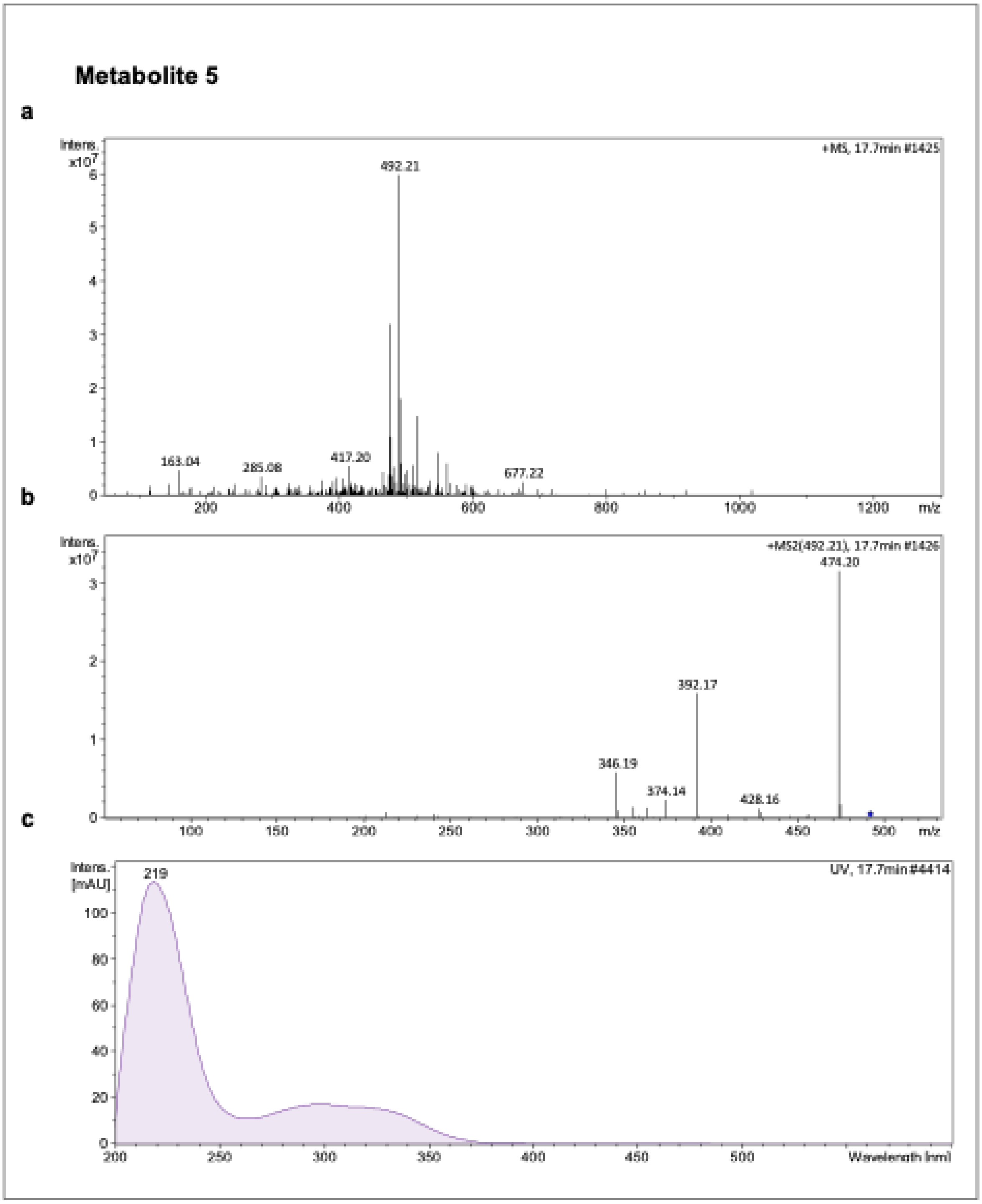

Figure S31. Spectrometric and spectroscopic data of metabolite **5**, indicated as a discriminant compound for *Vernonanthura polyanthes*, including MS spectra (**a**), MS^n spectra (**b**) and UV spectra (**c**).

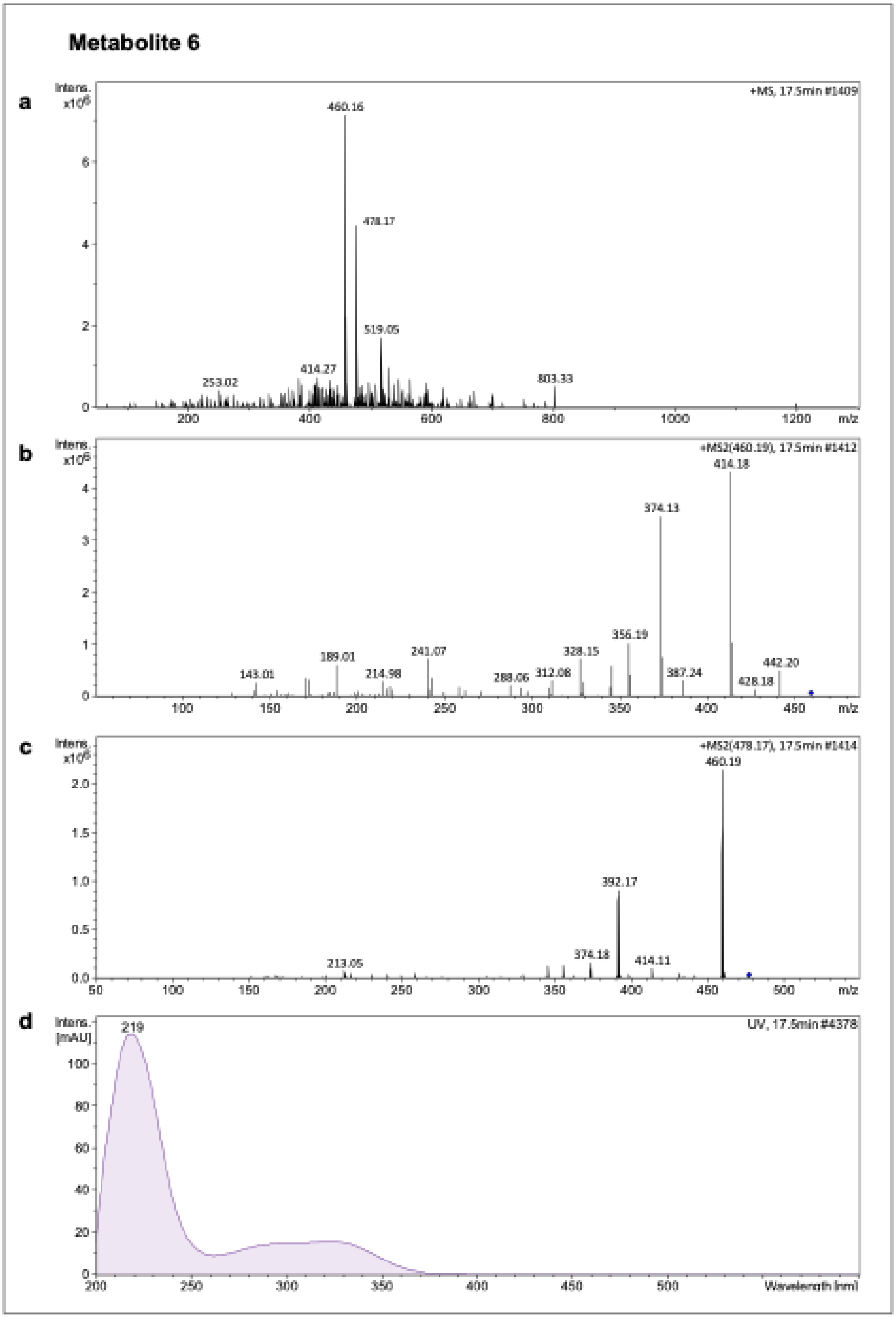

Figure S32. Spectrometric and spectroscopic data of metabolite **6**, indicated as a discriminant compound for *Vernonanthura polyanthes*, including MS spectra (**a**), MS^n spectra (**b** and **c**) and UV spectra (**d**).

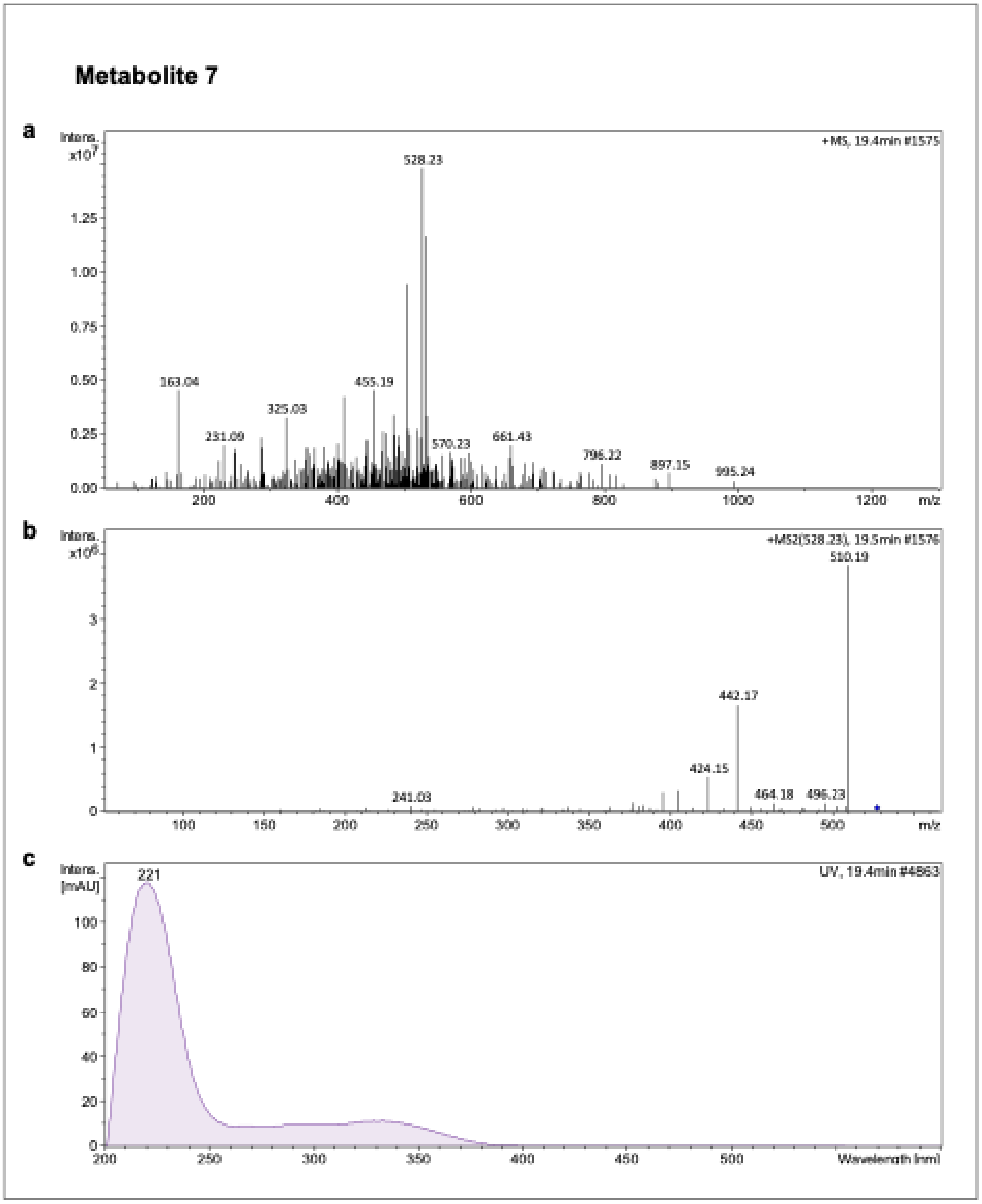

Figure S33. Spectrometric and spectroscopic data of metabolite **7**, indicated as a discriminant compound for *Vernonanthura polyanthes*, including MS spectra (**a**), MS^n spectra (**b**) and UV spectra (**c**).

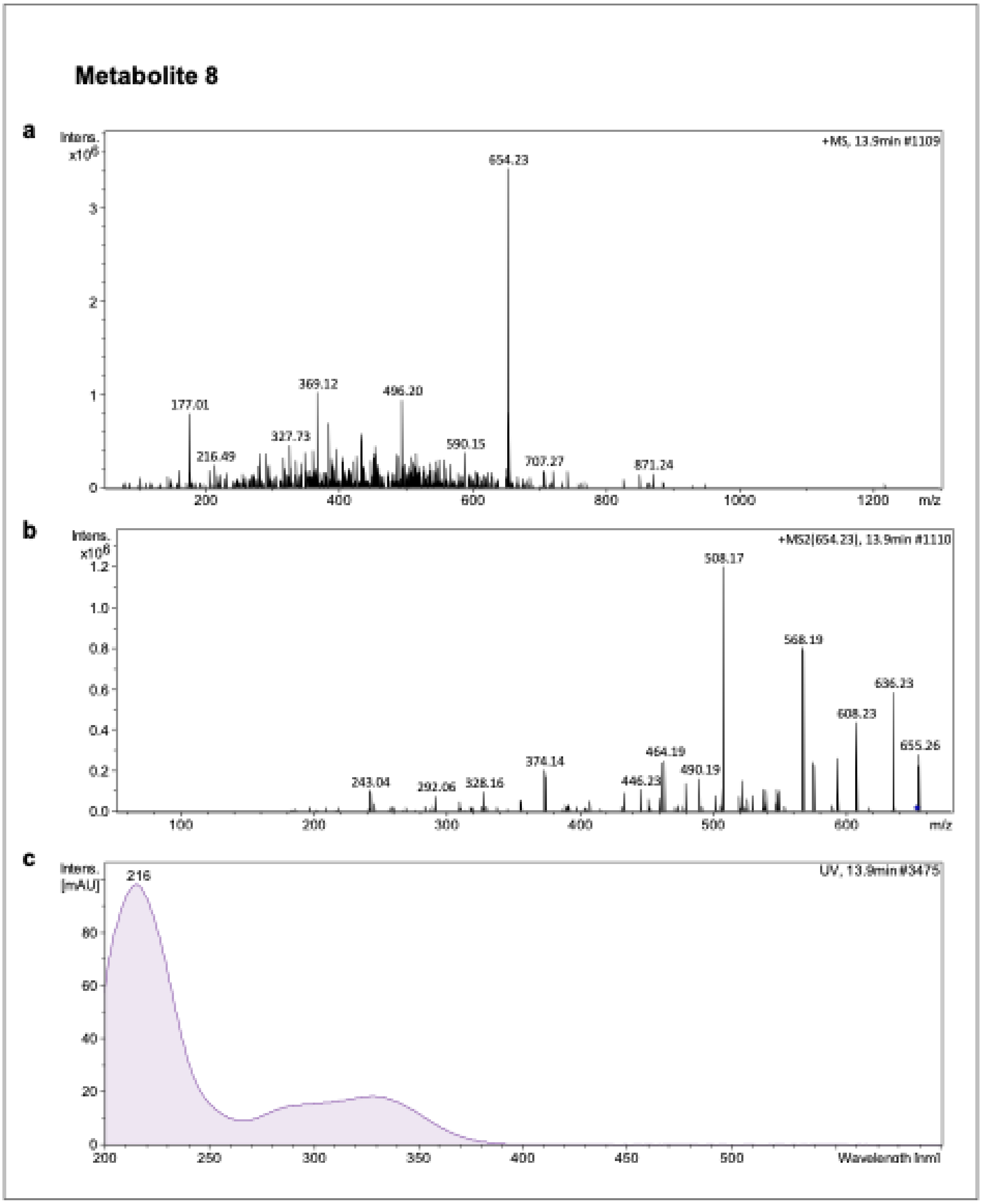

Figure S34. Spectrometric and spectroscopic data of metabolite **8**, indicated as a discriminant compound for *Vernonanthura polyanthes*, including MS spectra (**a**), MS^n spectra (**b**) and UV spectra (**c**).

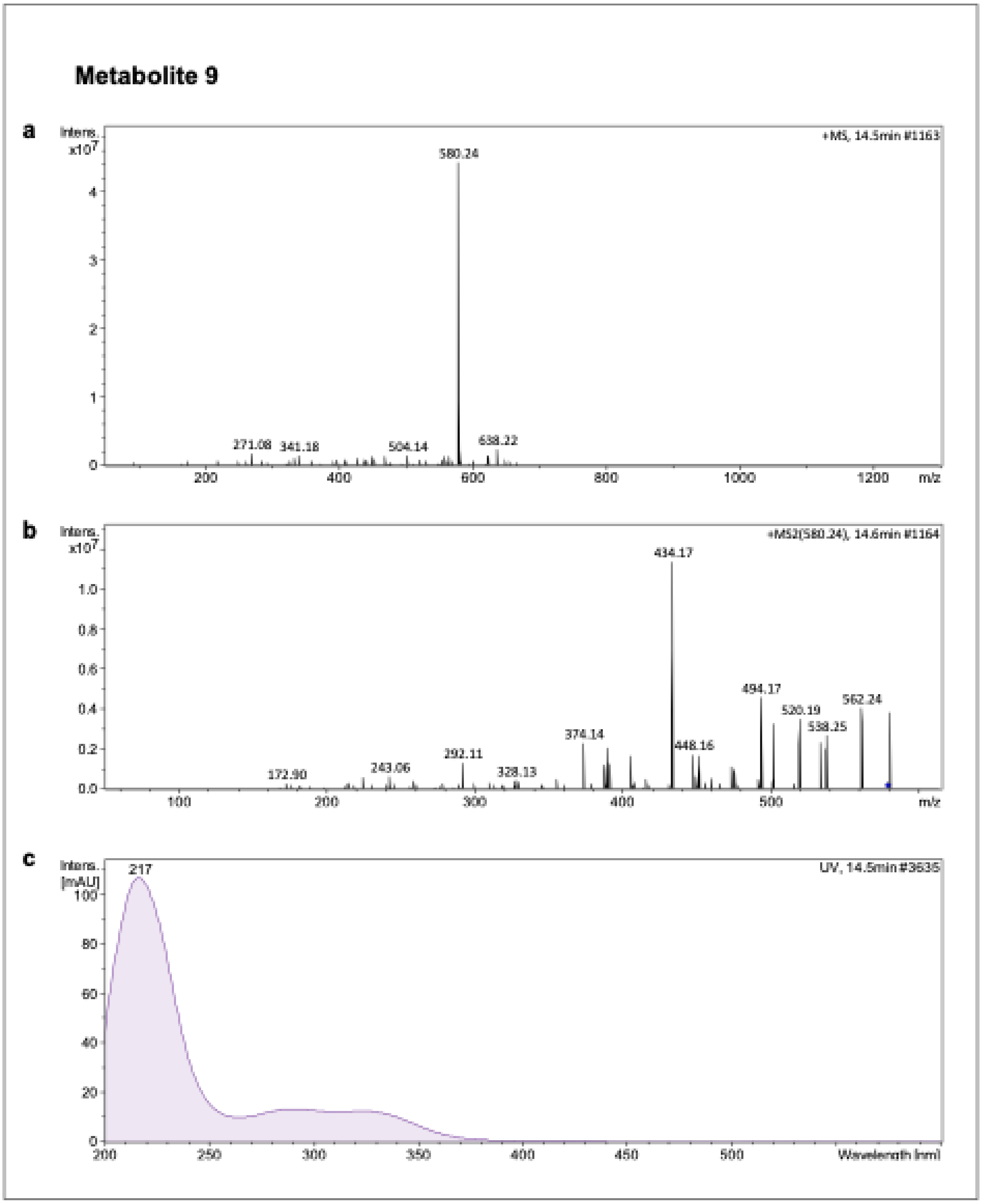

Figure S35. Spectrometric and spectroscopic data of metabolite **9**, indicated as a discriminant compound for *Vernonanthura polyanthes*, including MS spectra (**a**), MS^n spectra (**b**) and UV spectra (**c**).

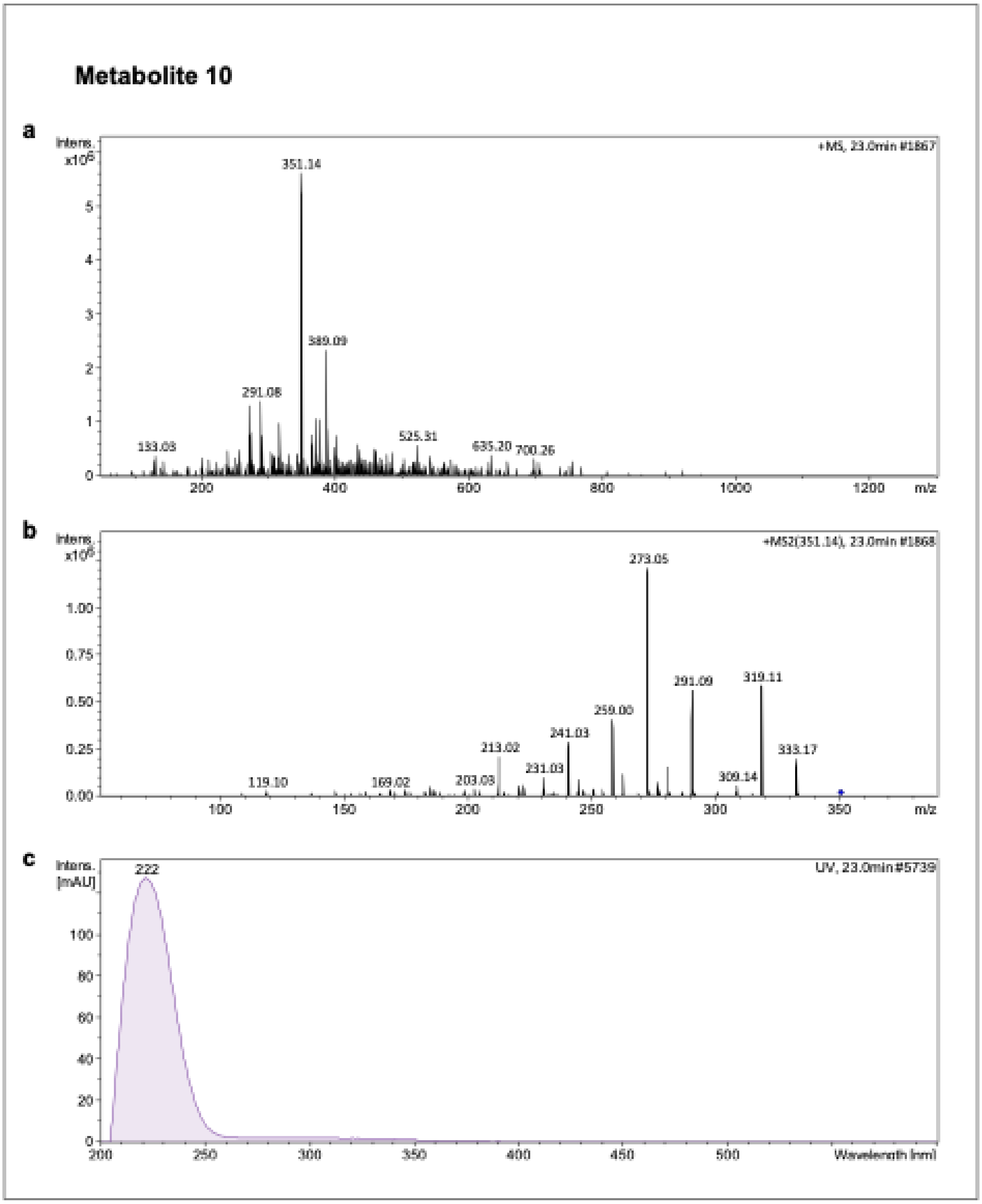

Figure S36. Spectrometric and spectroscopic data of metabolite **10**, indicated as a discriminant compound for *Vernonanthura polyanthes*, including MS spectra (**a**), MS^n spectra (**b**) and UV spectra (**c**).

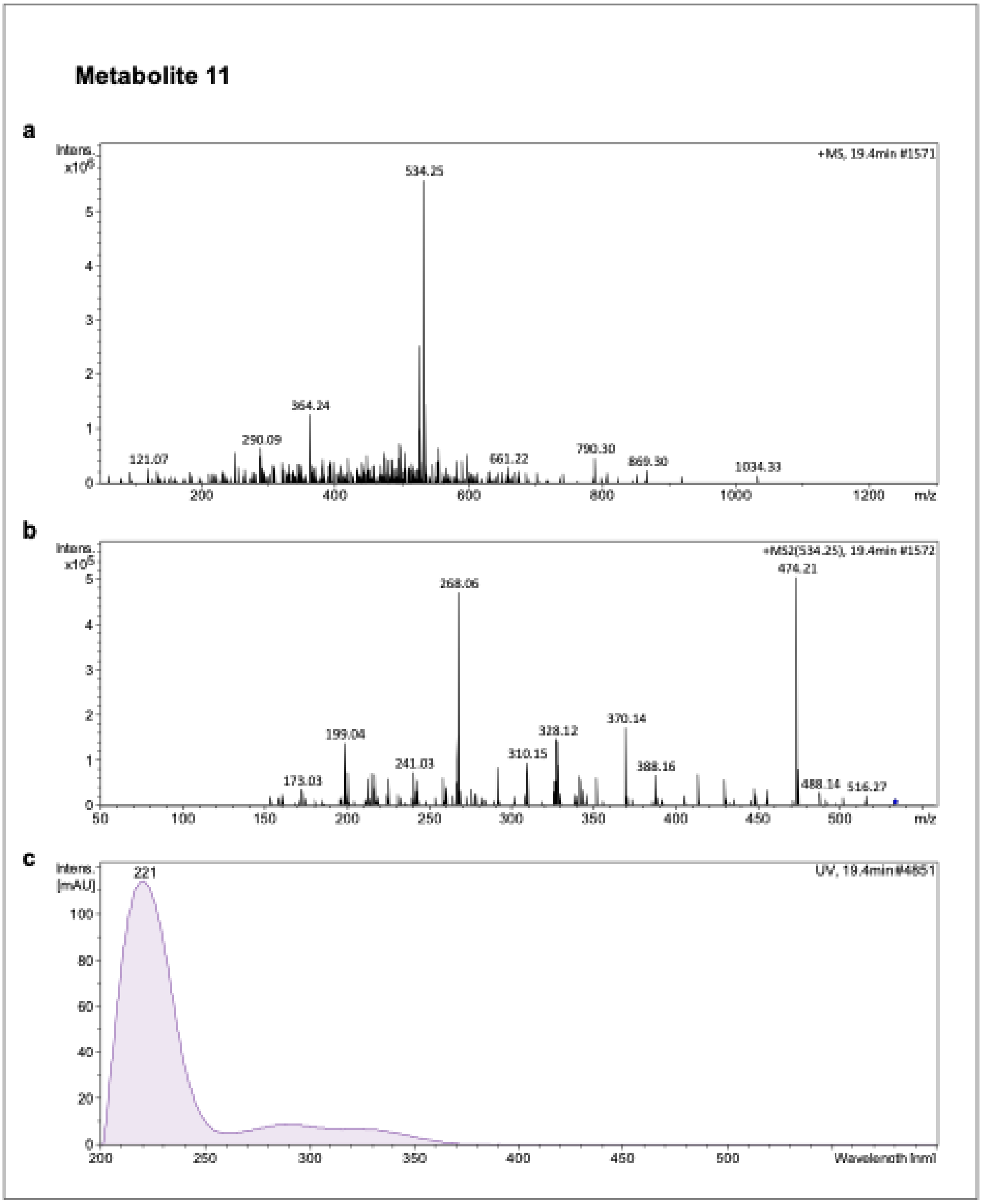

Figure S37. Spectrometric and spectroscopic data of metabolite **11**, indicated as a discriminant compound for *Vernonanthura polyanthes*, including MS spectra (**a**), MS^n spectra (**b**) and UV spectra (**c**).

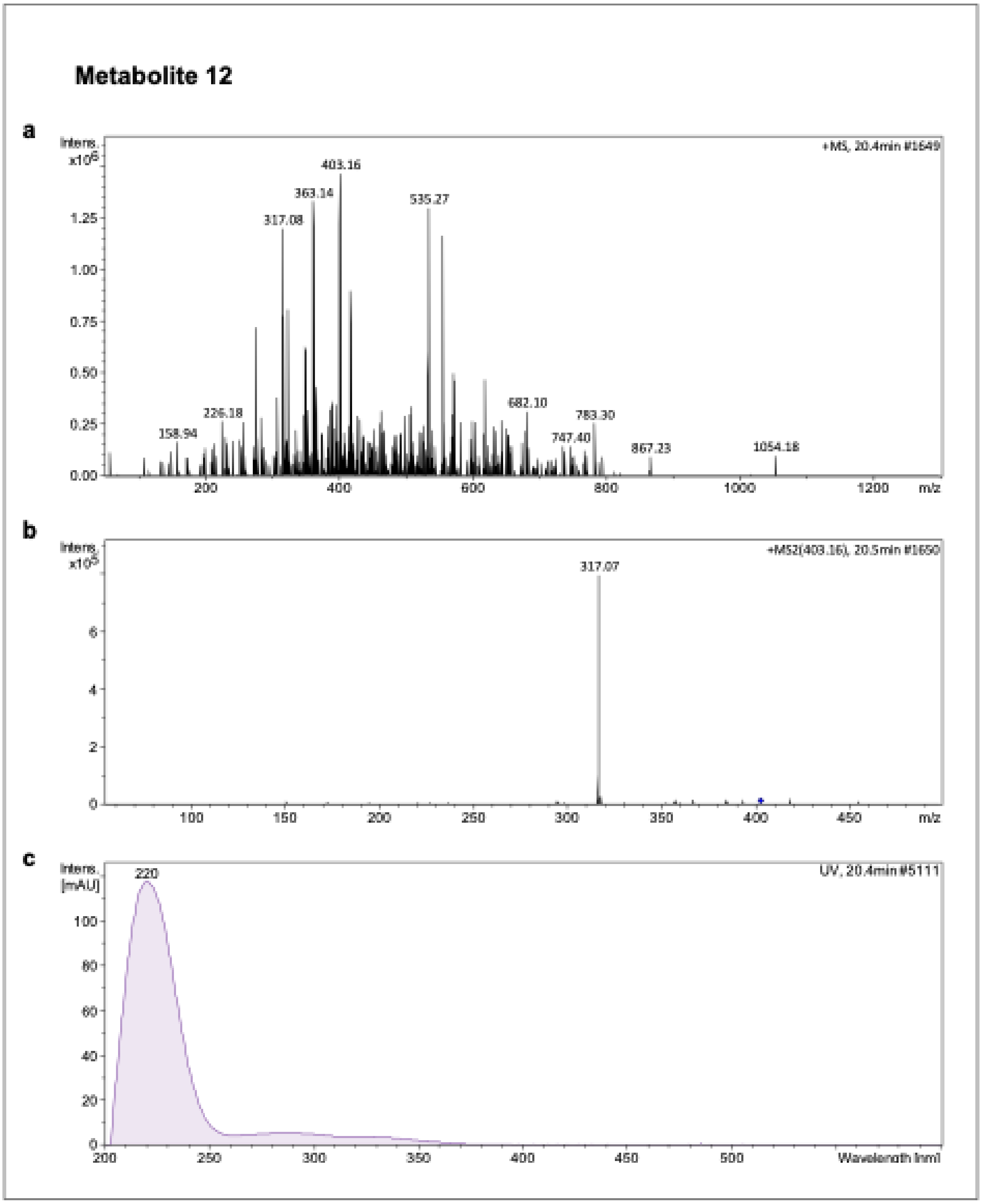

Figure S38. Spectrometric and spectroscopic data of metabolite **12**, indicated as a discriminant compound for *Vernonanthura polyanthes*, including MS spectra (**a**), MS^n spectra (**b**) and UV spectra (**c**).

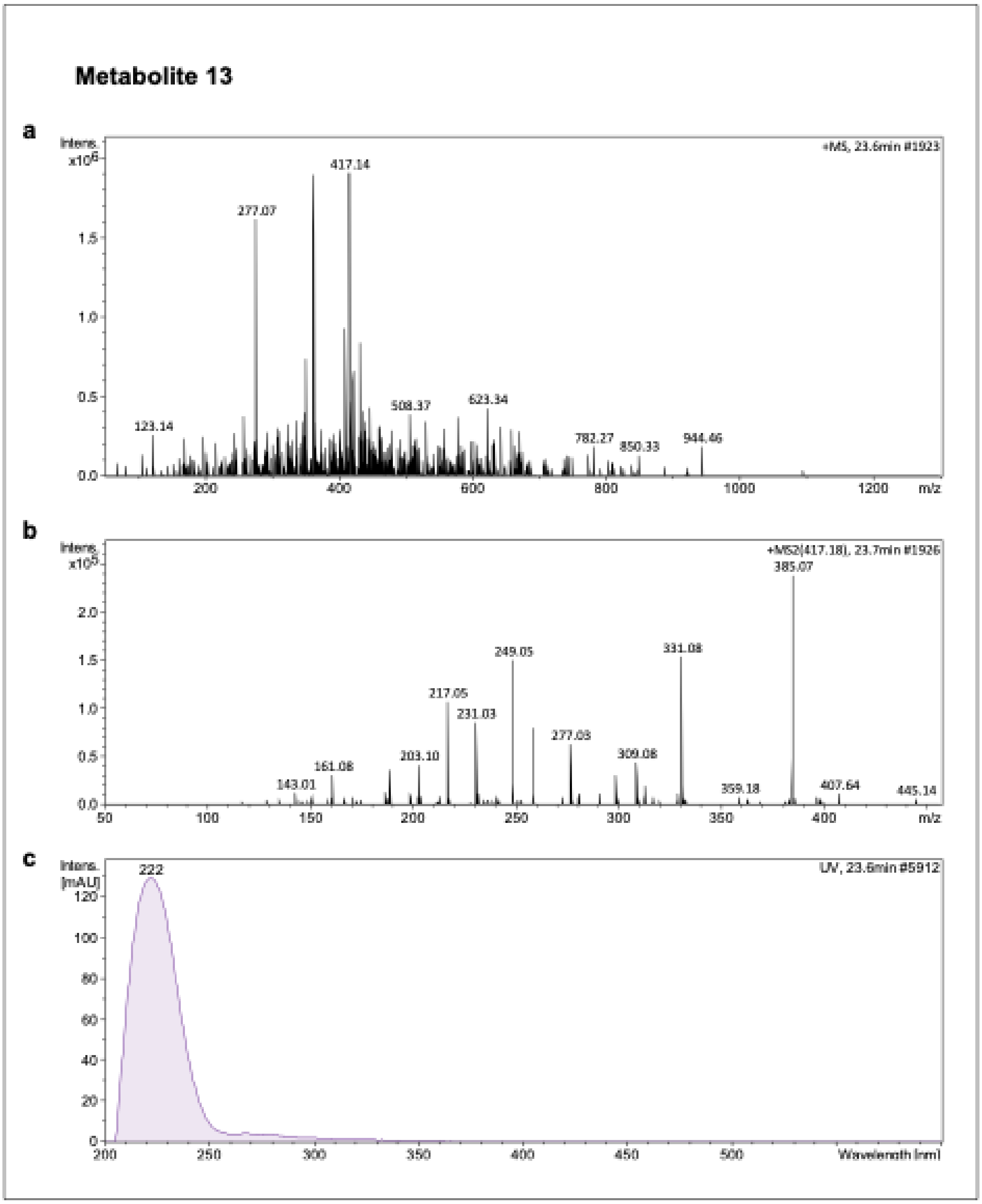

Figure S39. Spectrometric and spectroscopic data of metabolite **13**, indicated as a discriminant compound for *Vernonanthura polyanthes*, including MS spectra (**a**), MS^n spectra (**b**) and UV spectra (**c**).

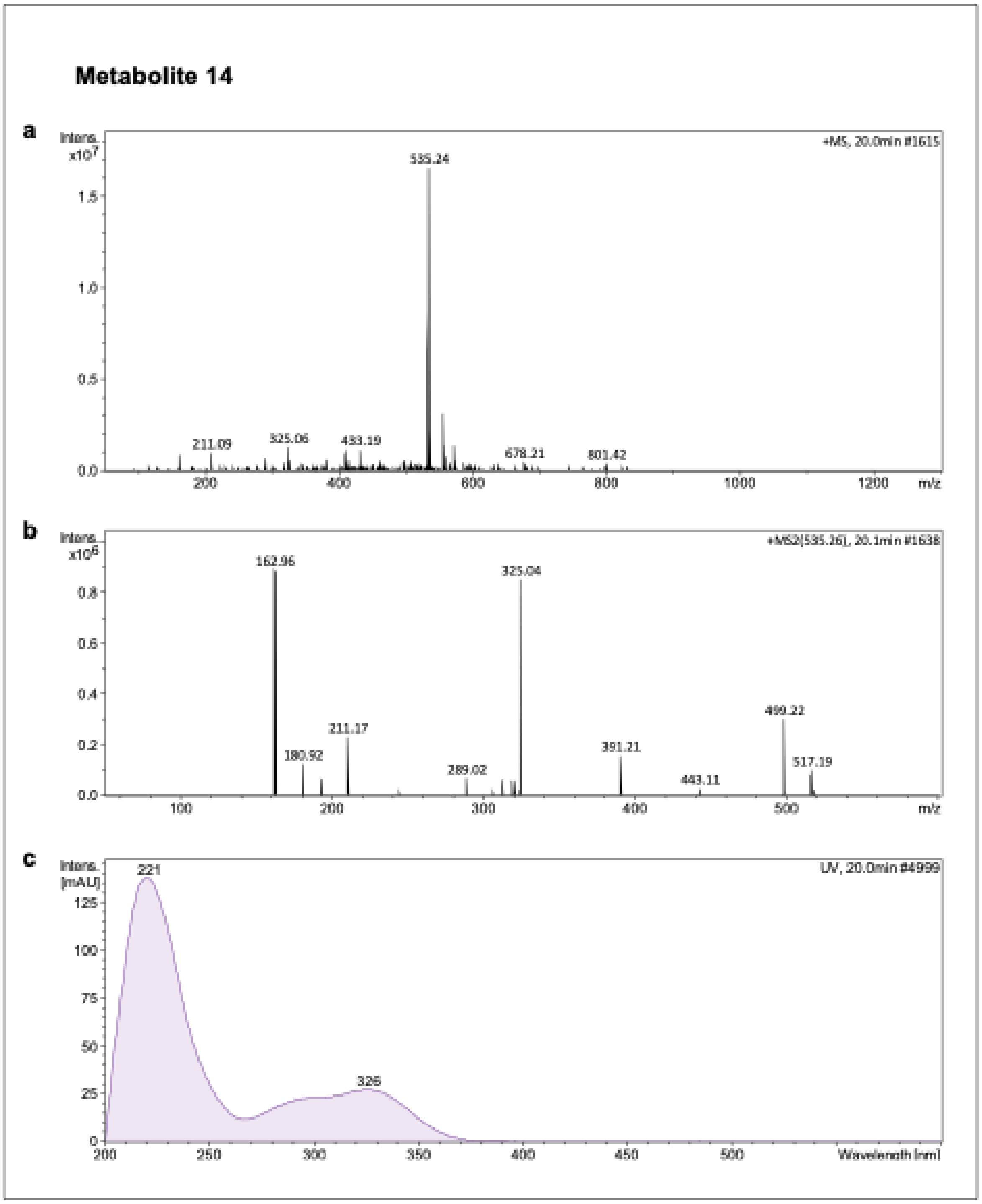

Figure S40. Spectrometric and spectroscopic data of metabolite **14**, indicated as a discriminant compound for *Vernonanthura polyanthes*, including MS spectra (**a**), MS^n spectra (**b**) and UV spectra (**c**).

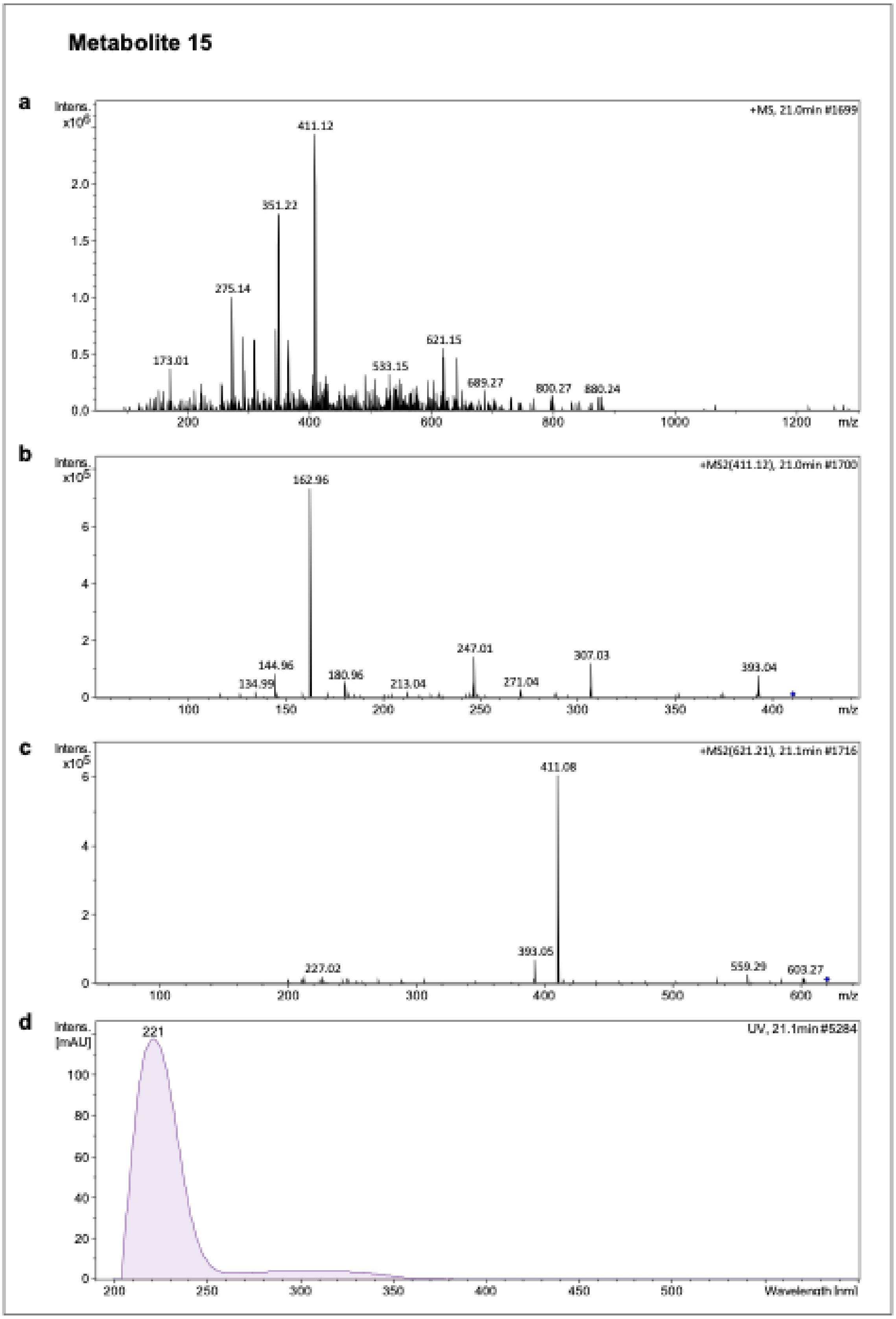

Figure S41. Spectrometric and spectroscopic data of metabolite **15**, indicated as a discriminant compound for *Vernonanthura polyanthes*, including MS spectra (**a**), MS^n spectra (**b** and **c**) and UV spectra (**d**).

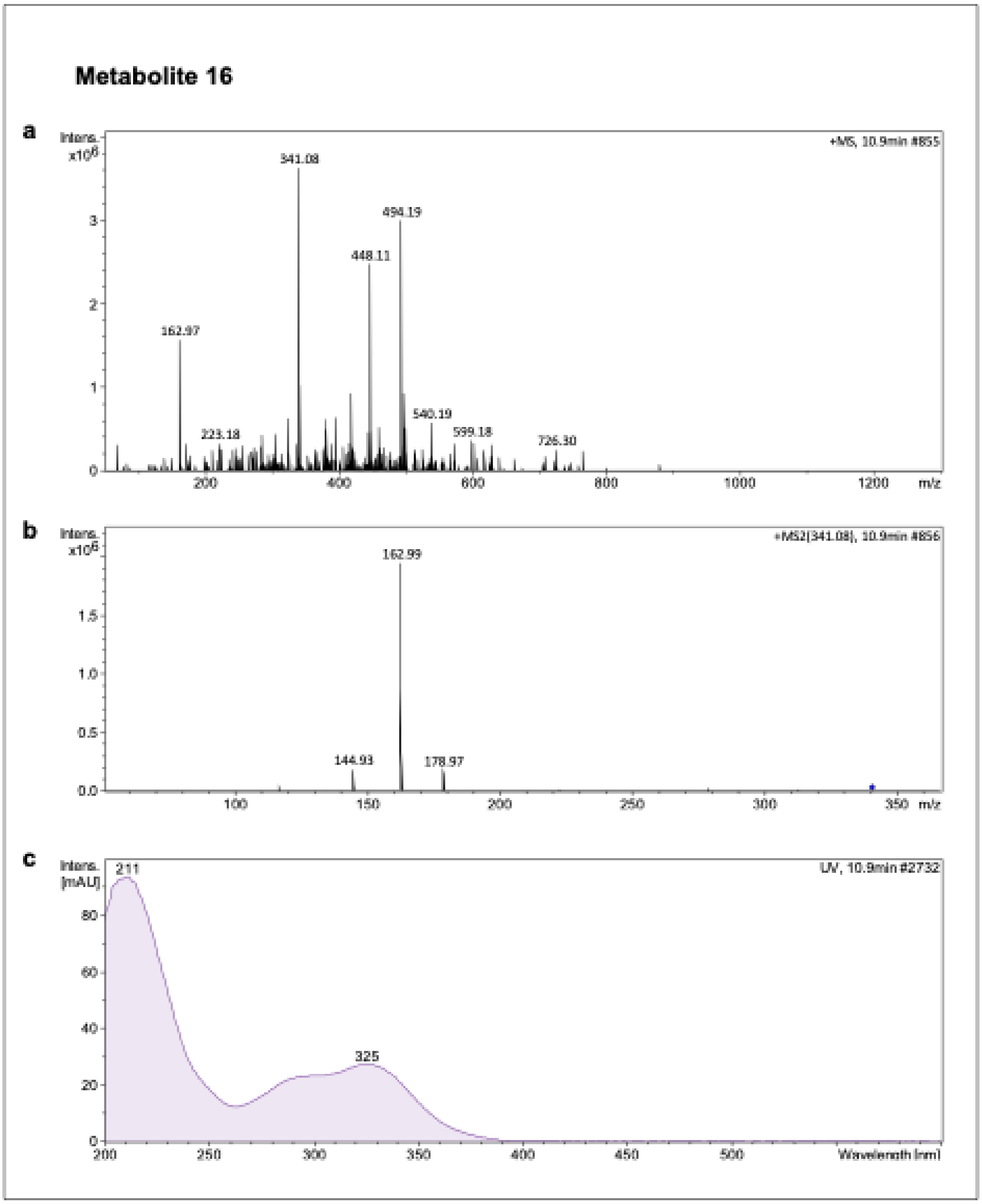

Figure S42. Spectrometric and spectroscopic data of metabolite **16**, indicated as a discriminant compound for *Vernonanthura polyanthes*, including MS spectra (**a**), MS^n spectra (**b**) and UV spectra (**c**).

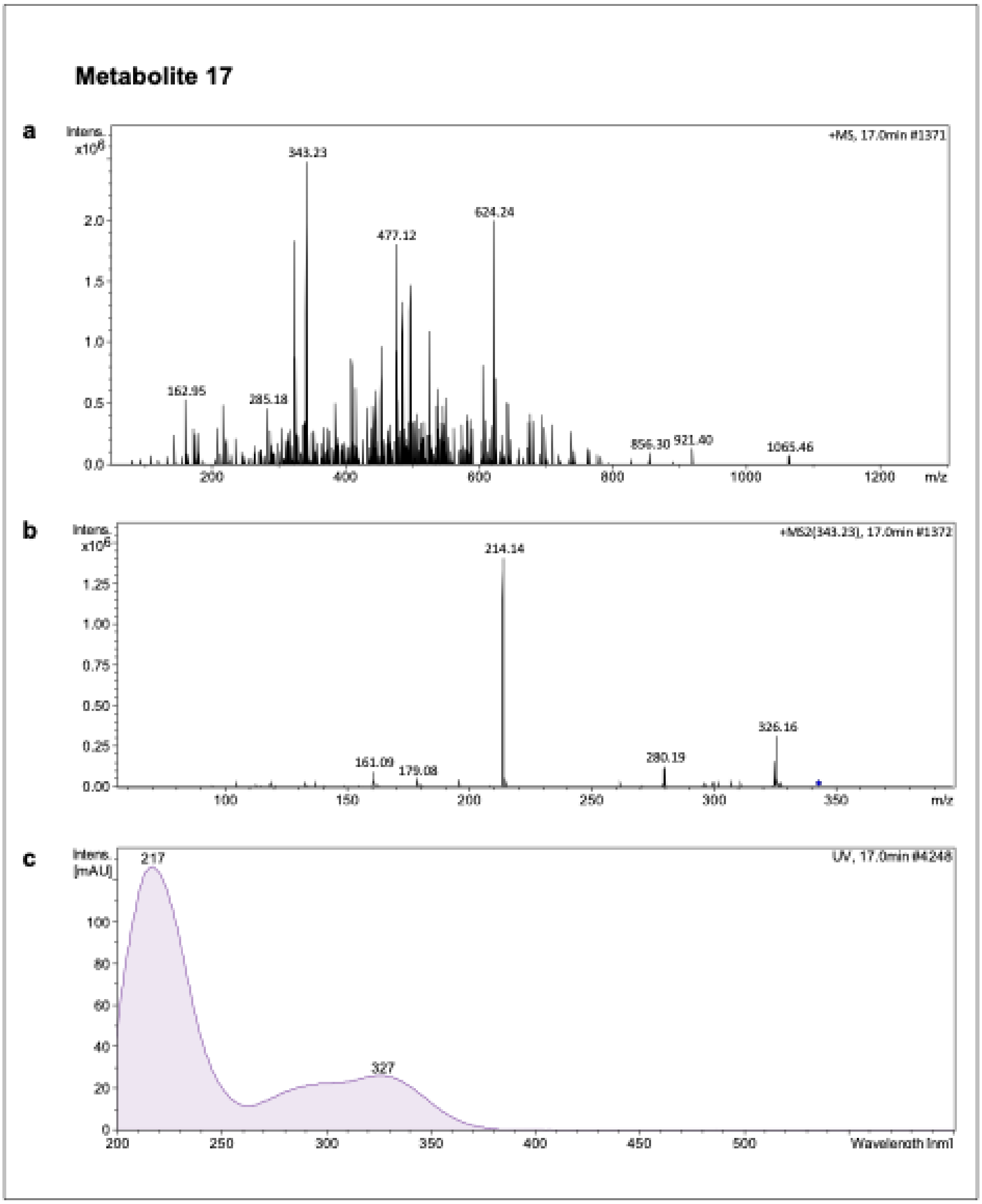

Figure S43. Spectrometric and spectroscopic data of metabolite **17**, indicated as a discriminant compound for *Vernonanthura polyanthes*, including MS spectra (**a**), MS^n spectra (**b**) and UV spectra (**c**).

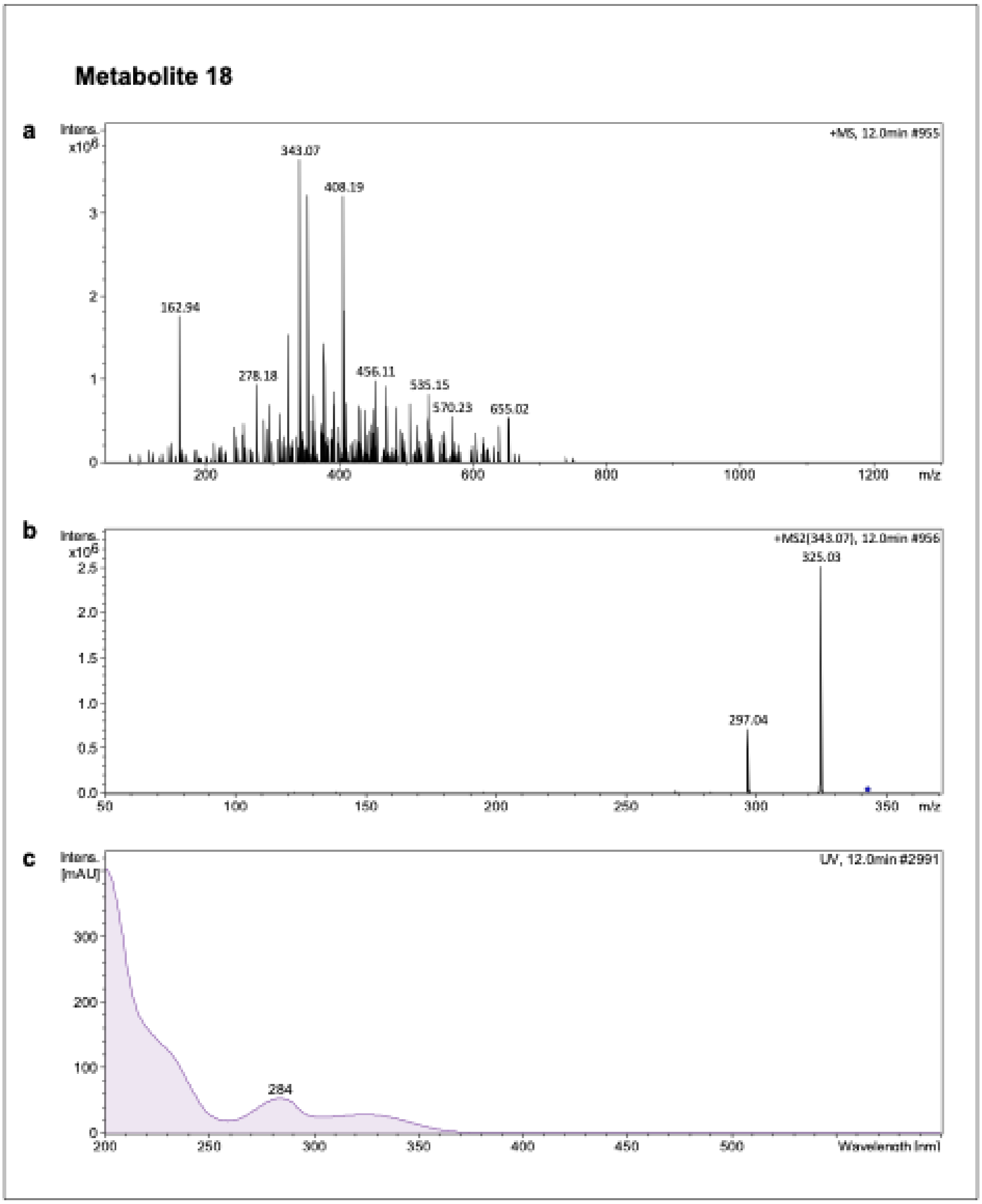

Figure S44. Spectrometric and spectroscopic data of metabolite **18**, indicated as a discriminant compound for *Vernonanthura polyanthes*, including MS spectra (**a**), MS^n spectra (**b**) and UV spectra (**c**).

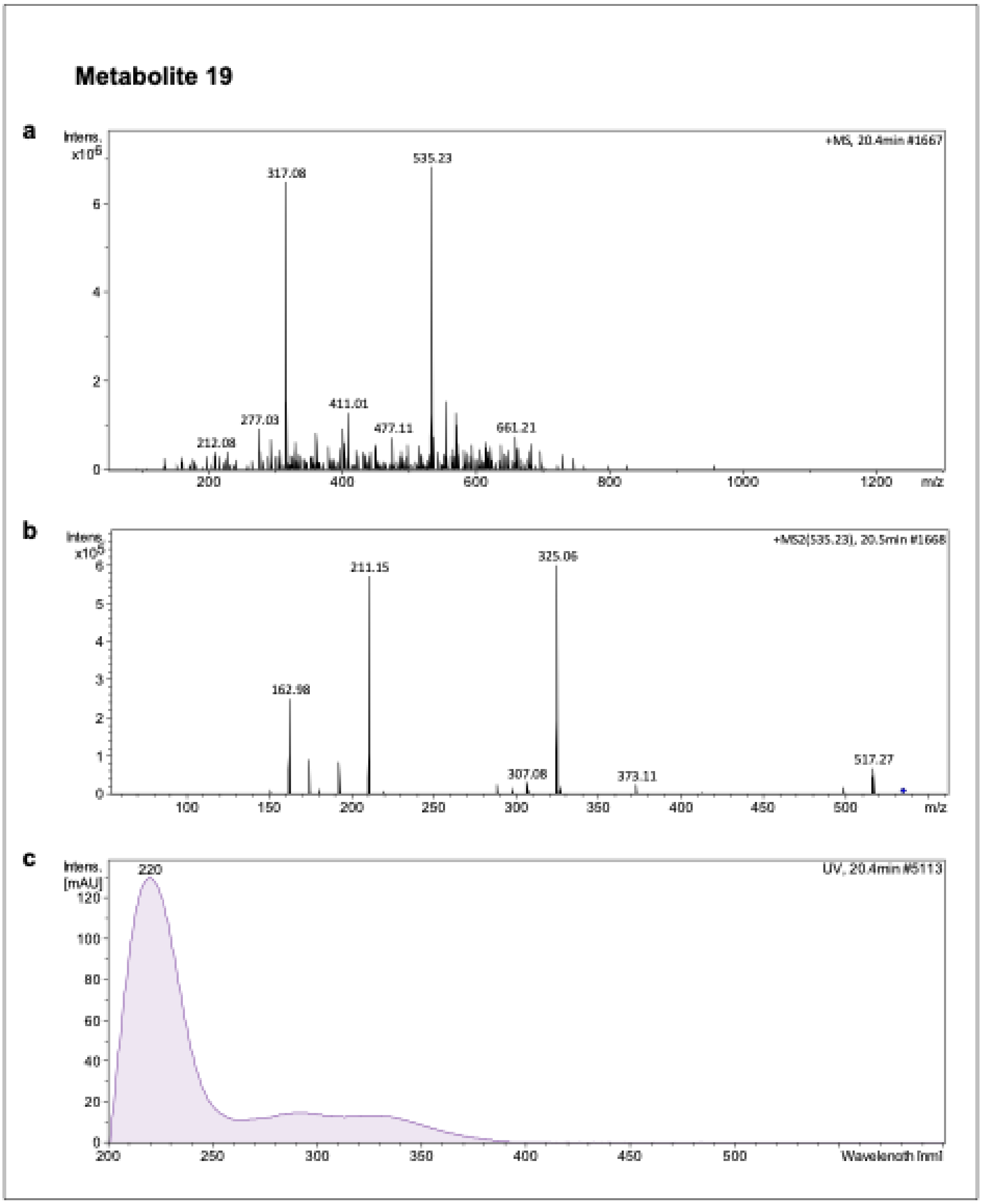

Figure S45. Spectrometric and spectroscopic data of metabolite **19**, indicated as a discriminant compound for *Vernonanthura polyanthes*, including MS spectra (**a**), MS^n spectra (**b**) and UV spectra (**c**).

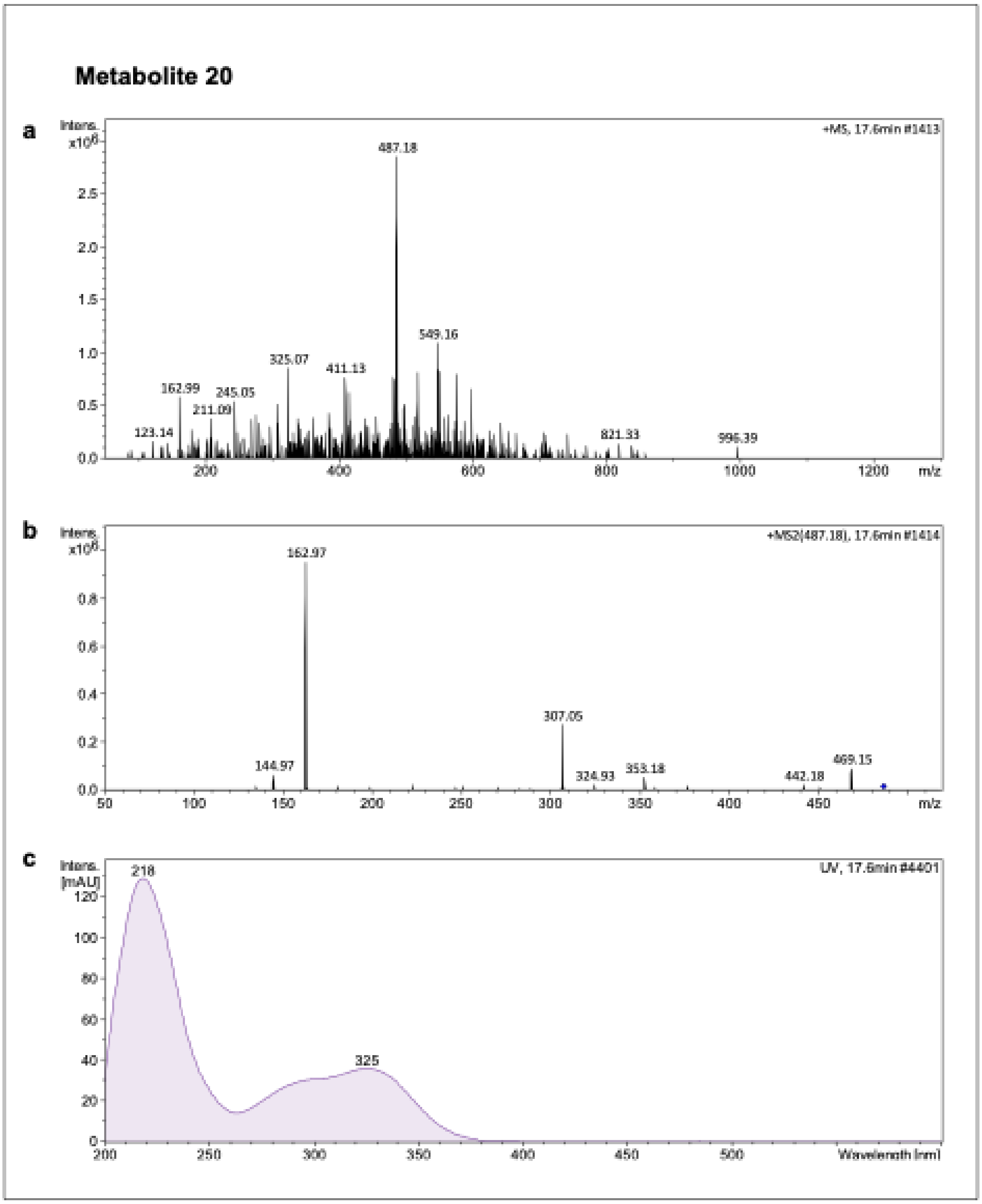

Figure S46. Spectrometric and spectroscopic data of metabolite **20**, indicated as a discriminant compound for *Vernonanthura polyanthes*, including MS spectra (**a**), MS^n spectra (**b**) and UV spectra (**c**).

Metabolite 21

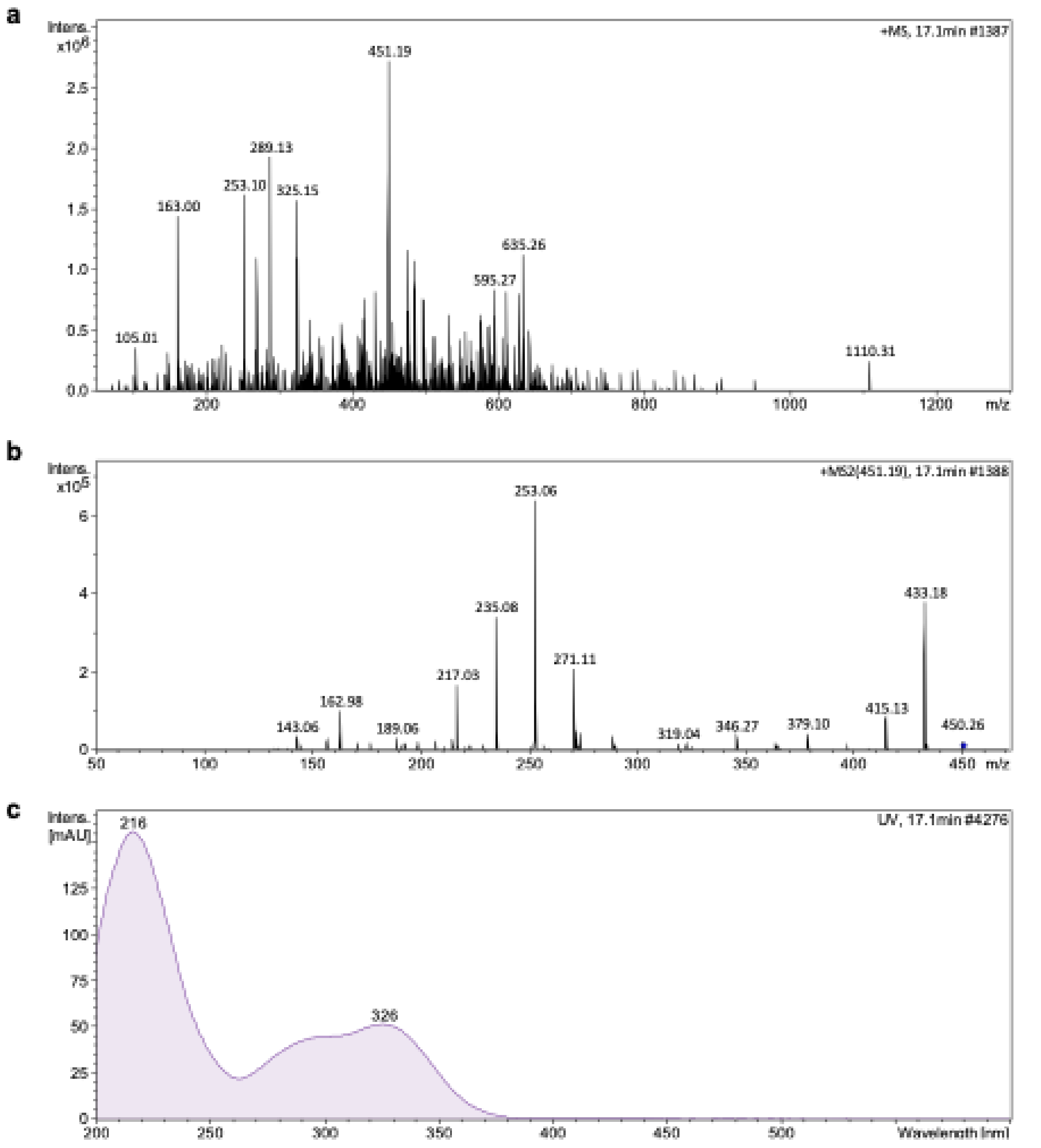

Figure S47. Spectrometric and spectroscopic data of metabolite **21**, indicated as a discriminant compound for *Vernonanthura polyanthes*, including MS spectra (**a**), MS^n spectra (**b**) and UV spectra (**c**).

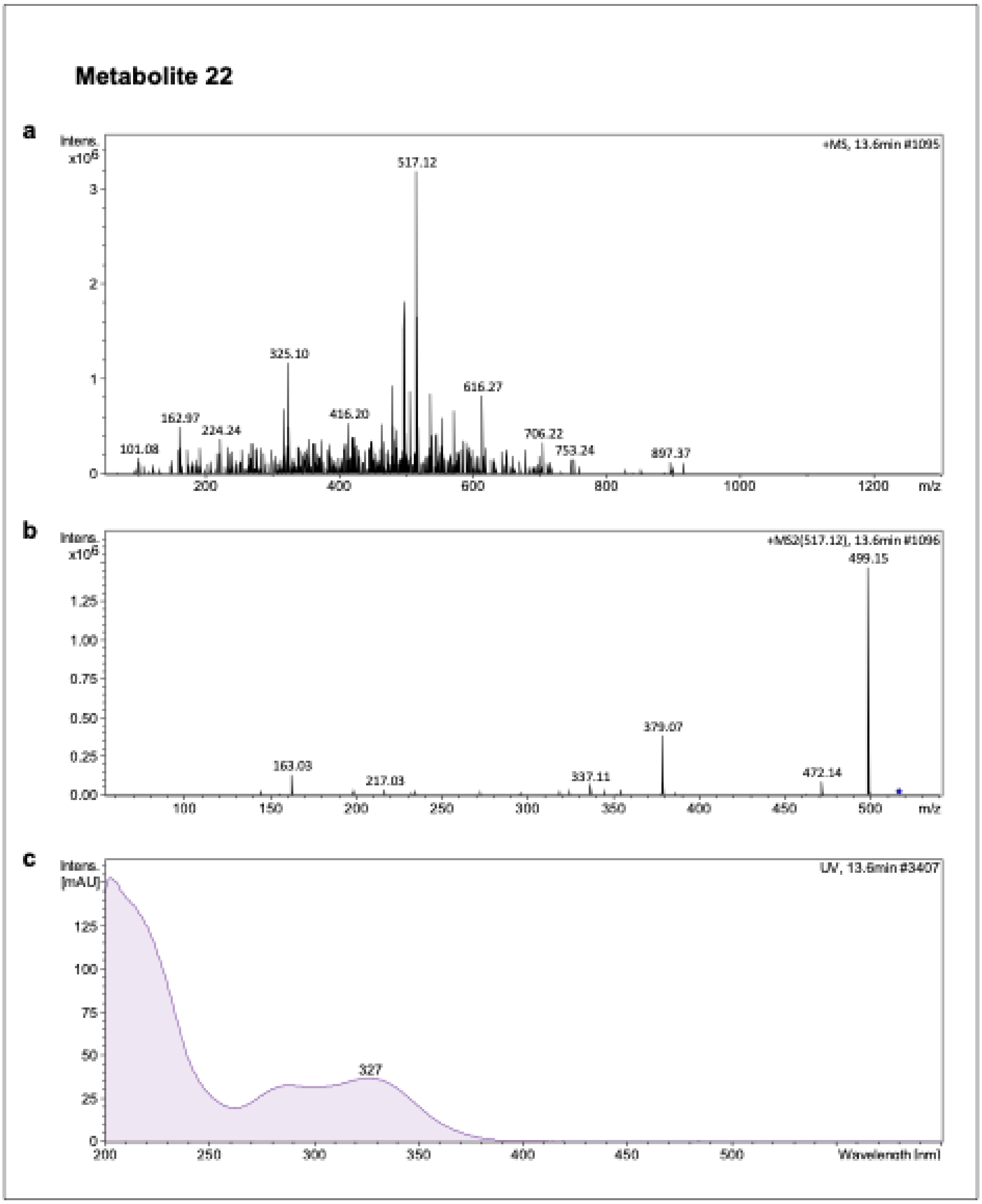

Figure S48. Spectrometric and spectroscopic data of metabolite **22**, indicated as a discriminant compound for *Vernonanthura polyanthes*, including MS spectra (**a**), MS^n spectra (**b**) and UV spectra (**c**).

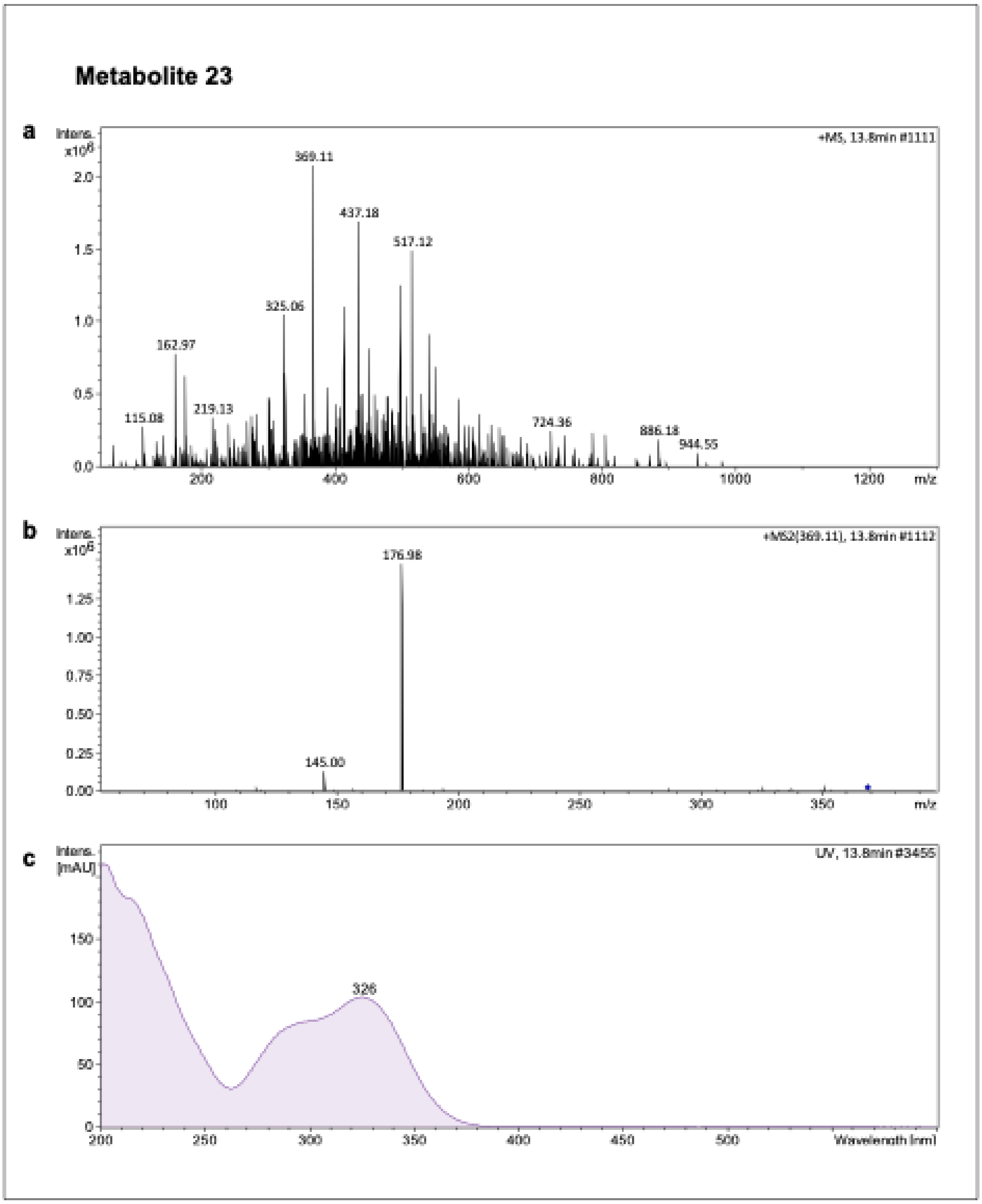

Figure S49. Spectrometric and spectroscopic data of metabolite **23**, indicated as a discriminant compound for *Vernonanthura polyanthes*, including MS spectra (**a**), MS^n spectra (**b**) and UV spectra (**c**).

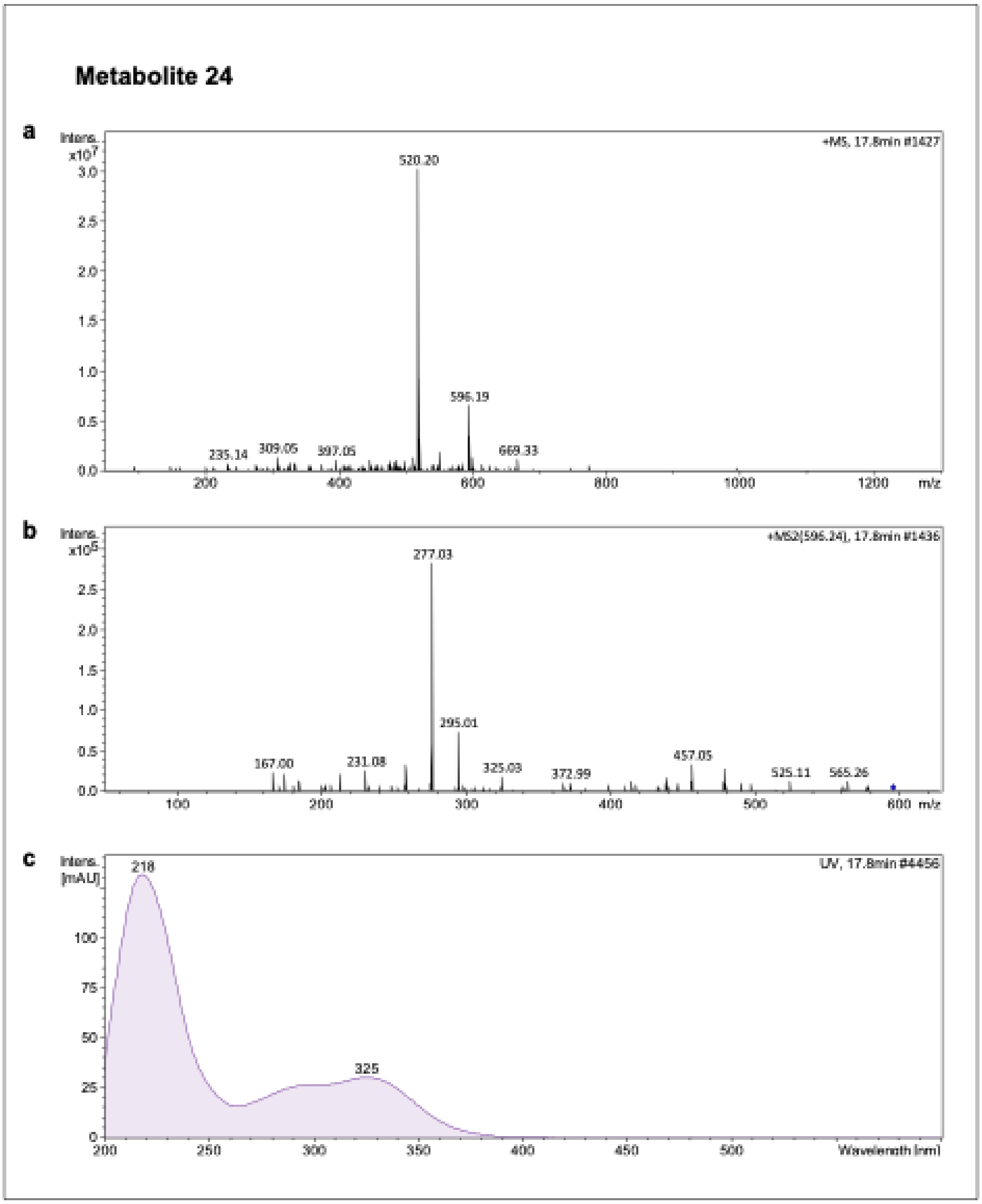

Figure S50. Spectrometric and spectroscopic data of metabolite **24**, indicated as a discriminant compound for *Vernonanthura polyanthes*, including MS spectra (**a**), MS^n spectra (**b**) and UV spectra (**c**).

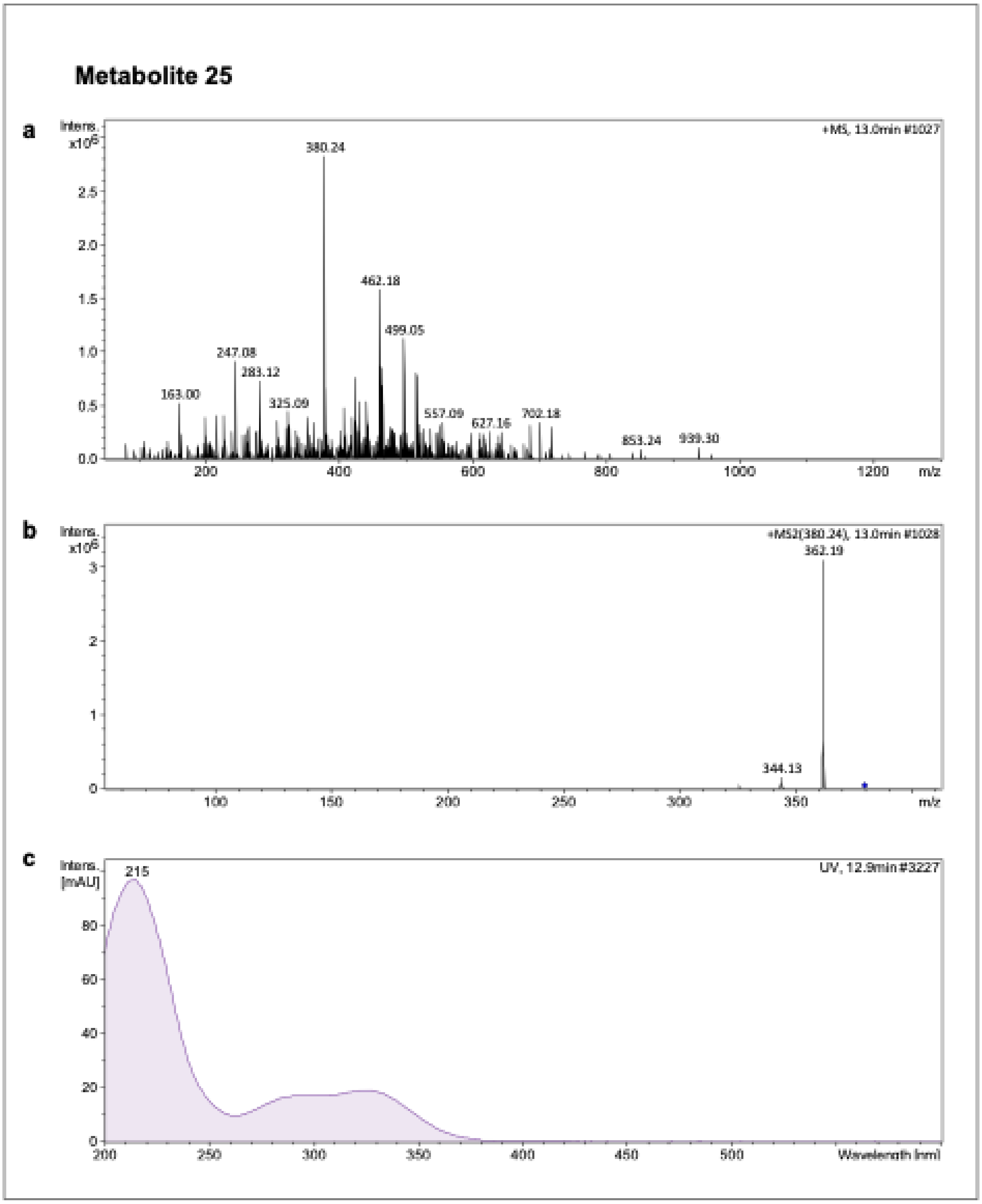

Figure S51. Spectrometric and spectroscopic data of metabolite **25**, indicated as a discriminant compound for *Vernonanthura polyanthes*, including MS spectra (**a**), MS^n spectra (**b**) and UV spectra (**c**).

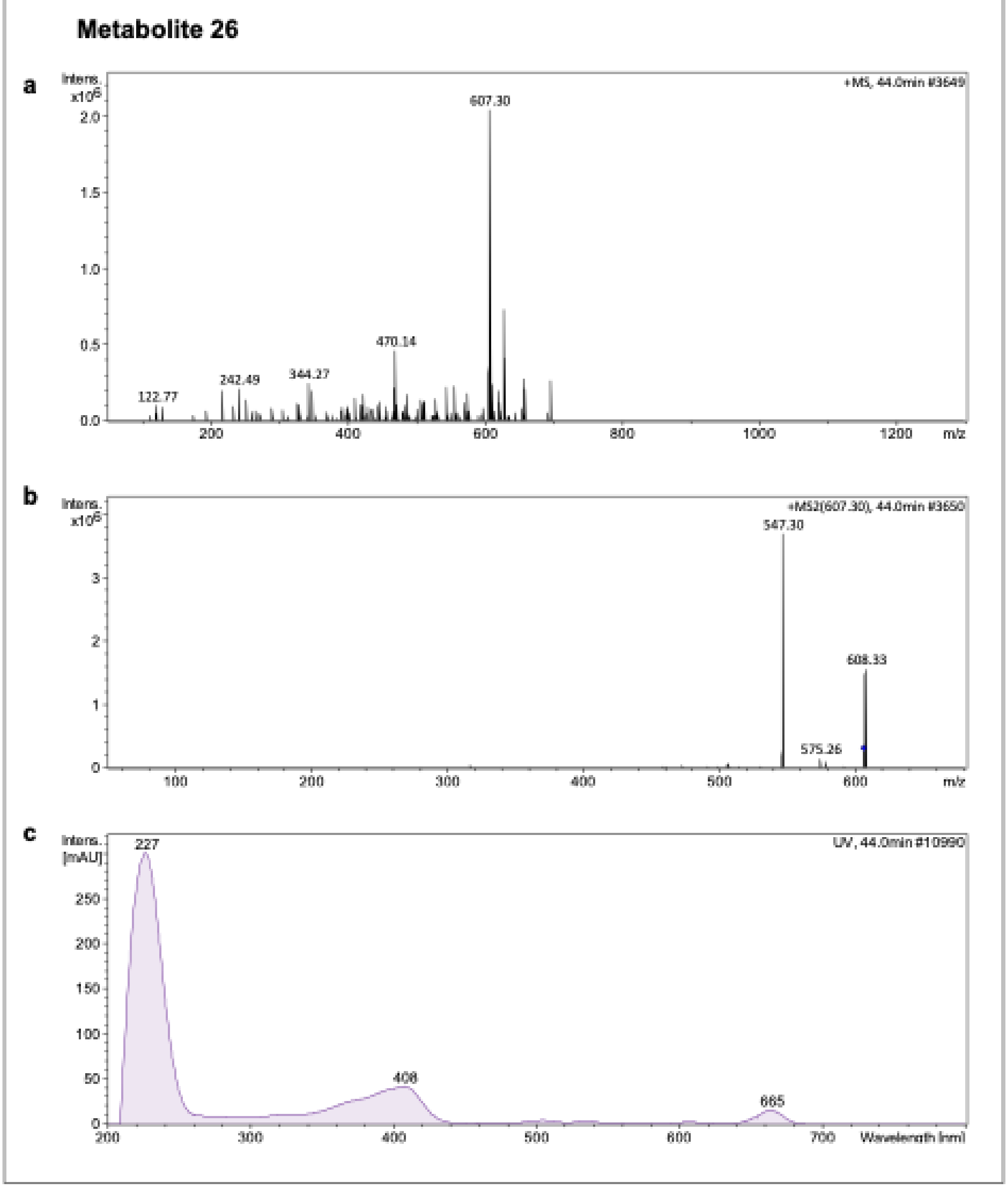

Figure S52. Spectrometric and spectroscopic data of metabolite **26**, indicated as a discriminant compound for *Piper aduncum*, including MS spectra (**a**), MS^n spectra (**b**) and UV spectra (**c**).

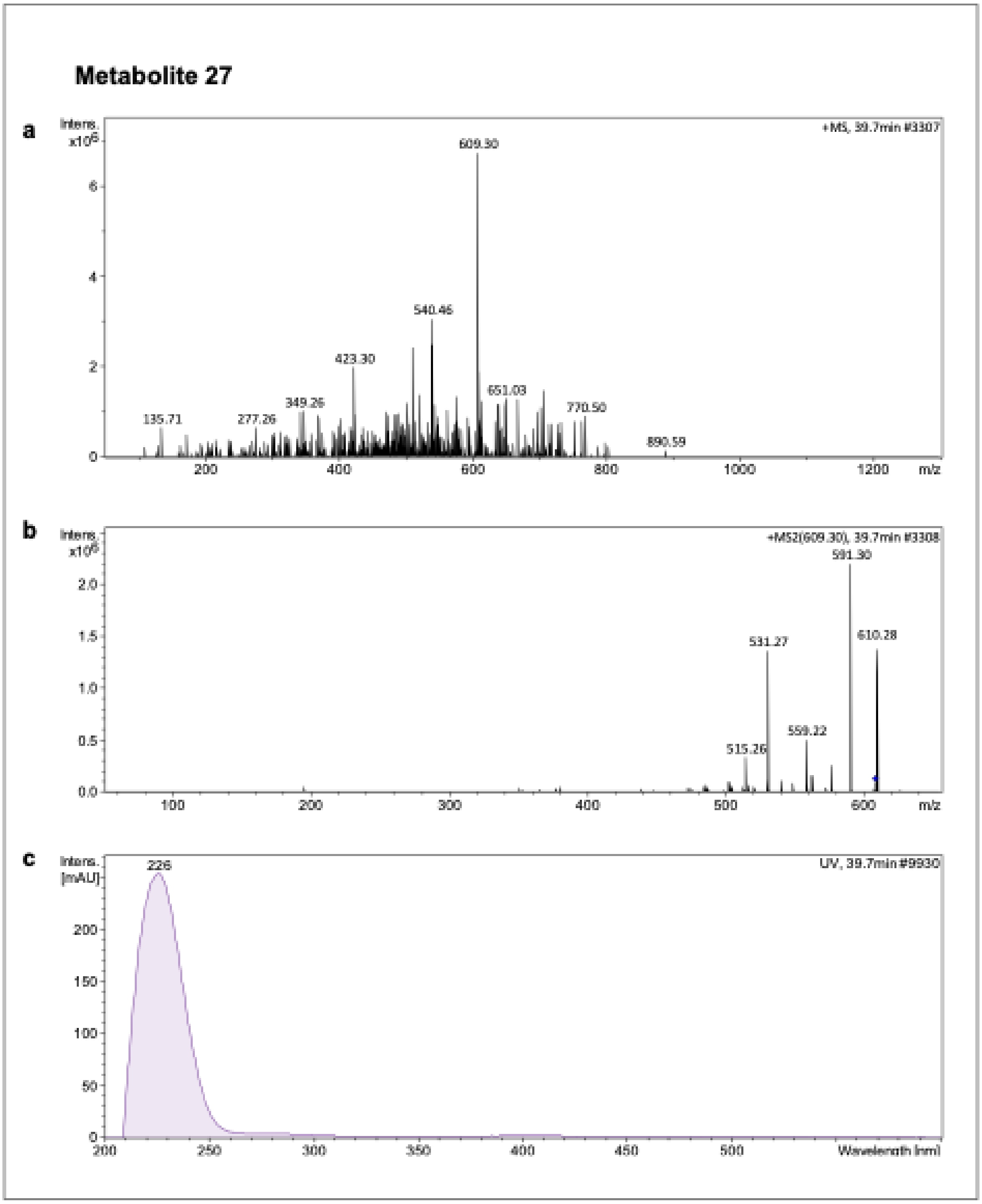

Figure S53. Spectrometric and spectroscopic data of metabolite **27**, indicated as a discriminant compound for *Piper aduncum*, including MS spectra (**a**), MS^n spectra (**b**) and UV spectra (**c**).

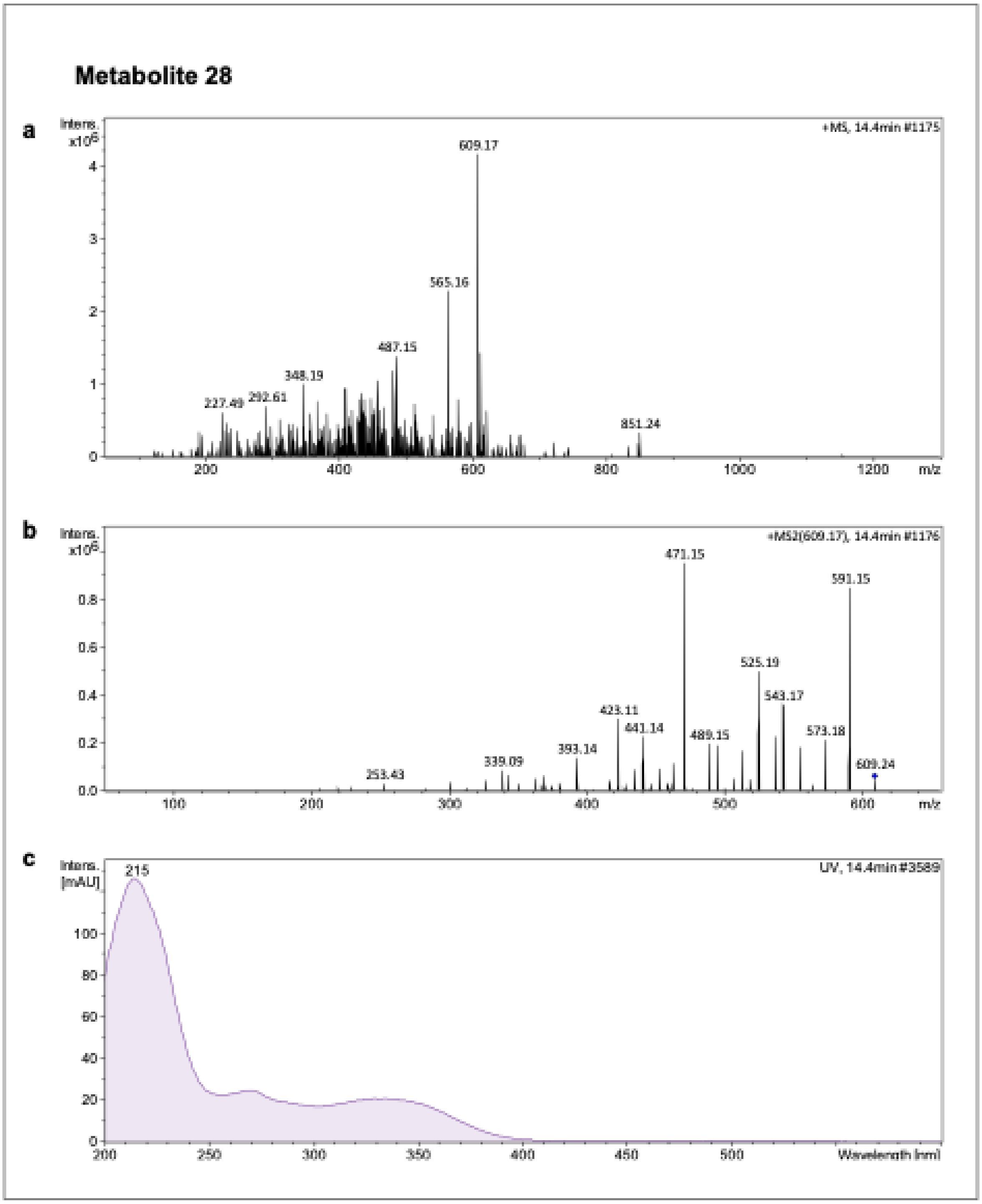

Figure S54. Spectrometric and spectroscopic data of metabolite **28**, indicated as a discriminant compound for *Piper aduncum*, including MS spectra (**a**), MS^n spectra (**b**) and UV spectra (**c**).

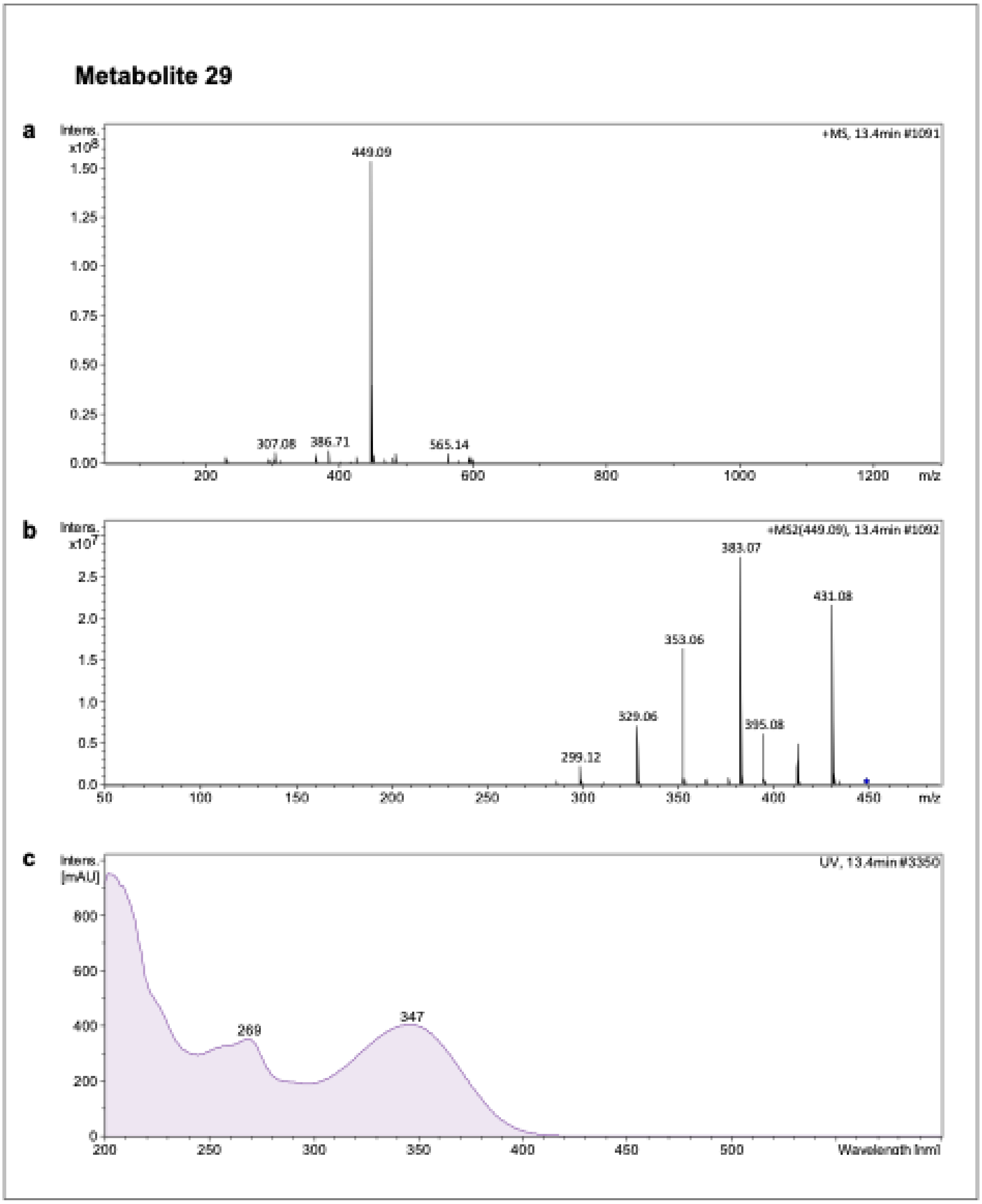

Figure S55. Spectrometric and spectroscopic data of metabolite **29**, indicated as a discriminant compound for *Piper aduncum*, including MS spectra (**a**), MS^n spectra (**b**) and UV spectra (**c**).

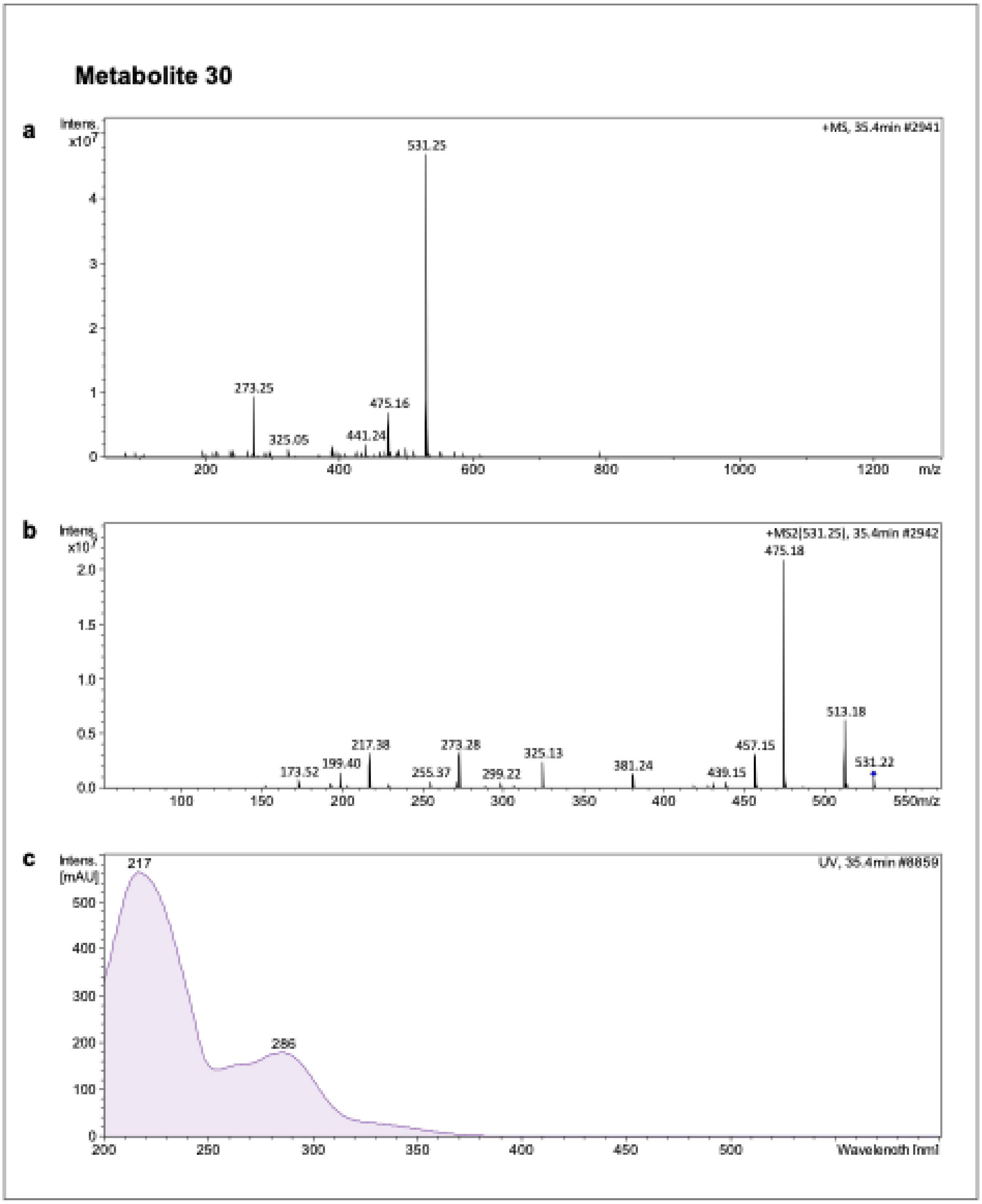

Figure S56. Spectrometric and spectroscopic data of metabolite **30**, indicated as a discriminant compound for *Piper aduncum*, including MS spectra (**a**), MS^n spectra (**b**) and UV spectra (**c**).

Metabolite 31

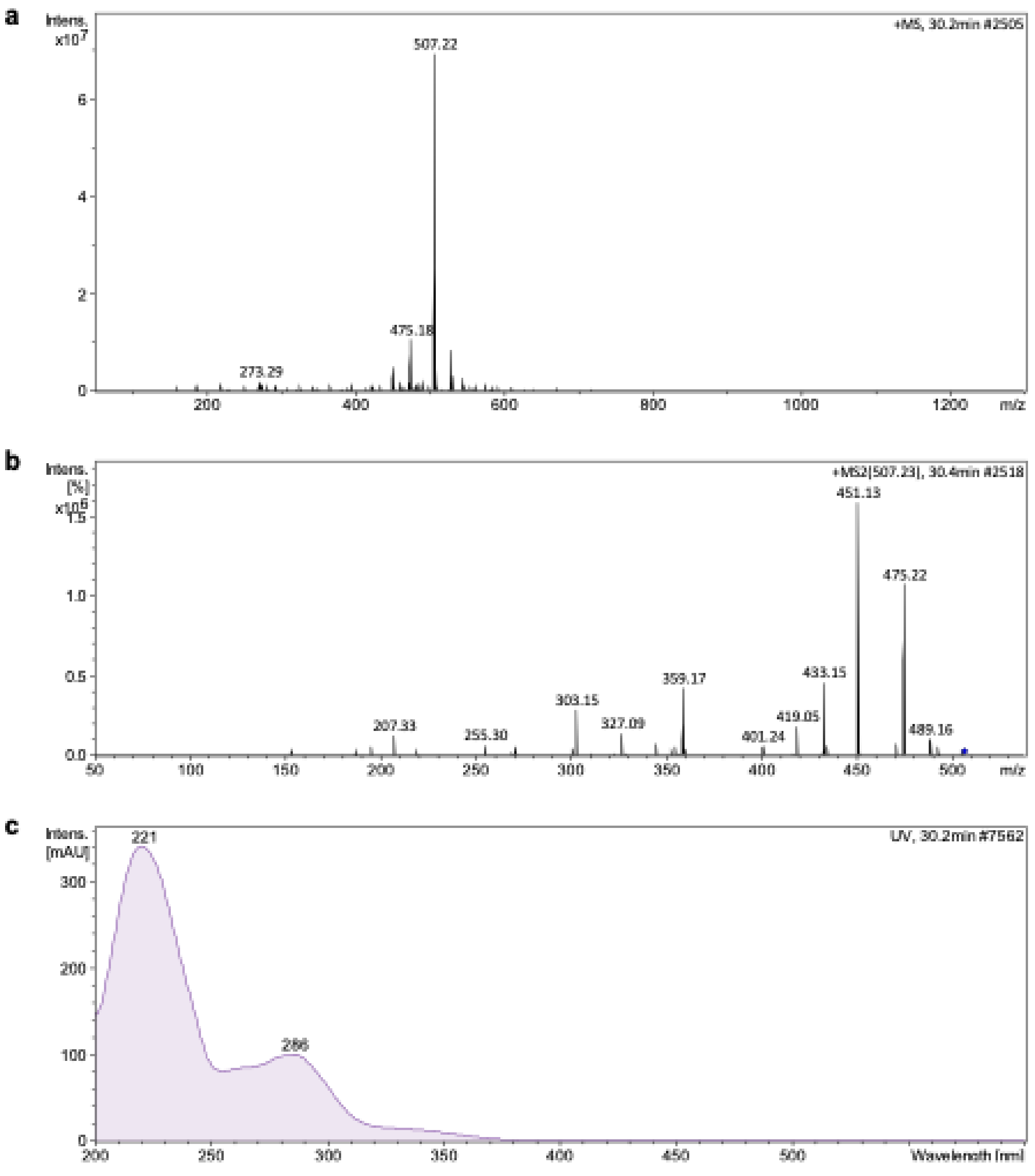

Figure S57. Spectrometric and spectroscopic data of metabolite **31**, indicated as a discriminant compound for *Piper aduncum*, including MS spectra (**a**), MS^n spectra (**b**) and UV spectra (**c**).

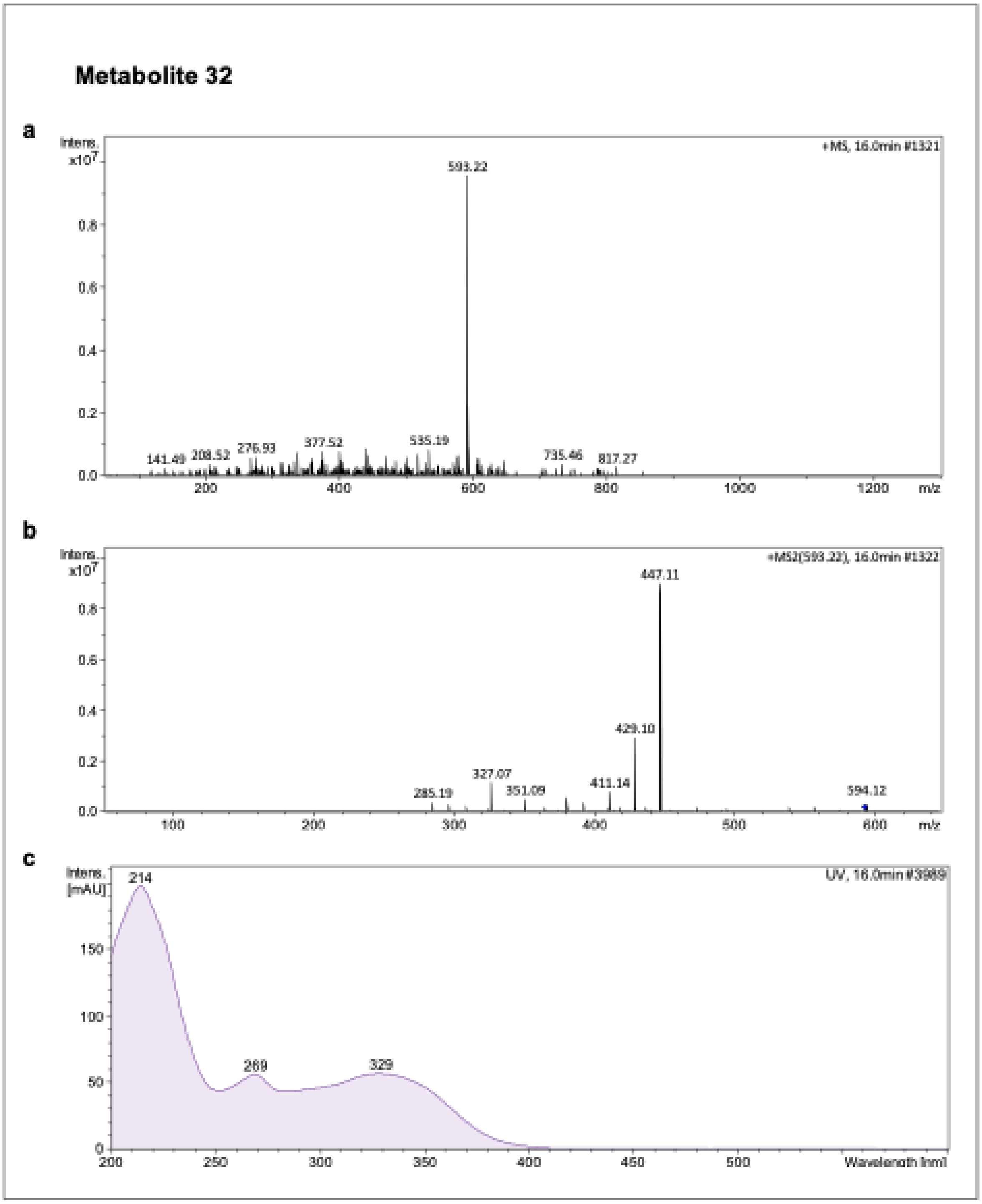

Figure S58. Spectrometric and spectroscopic data of metabolite **32**, indicated as a discriminant compound for *Piper aduncum*, including MS spectra (**a**), MS^n spectra (**b**) and UV spectra (**c**).

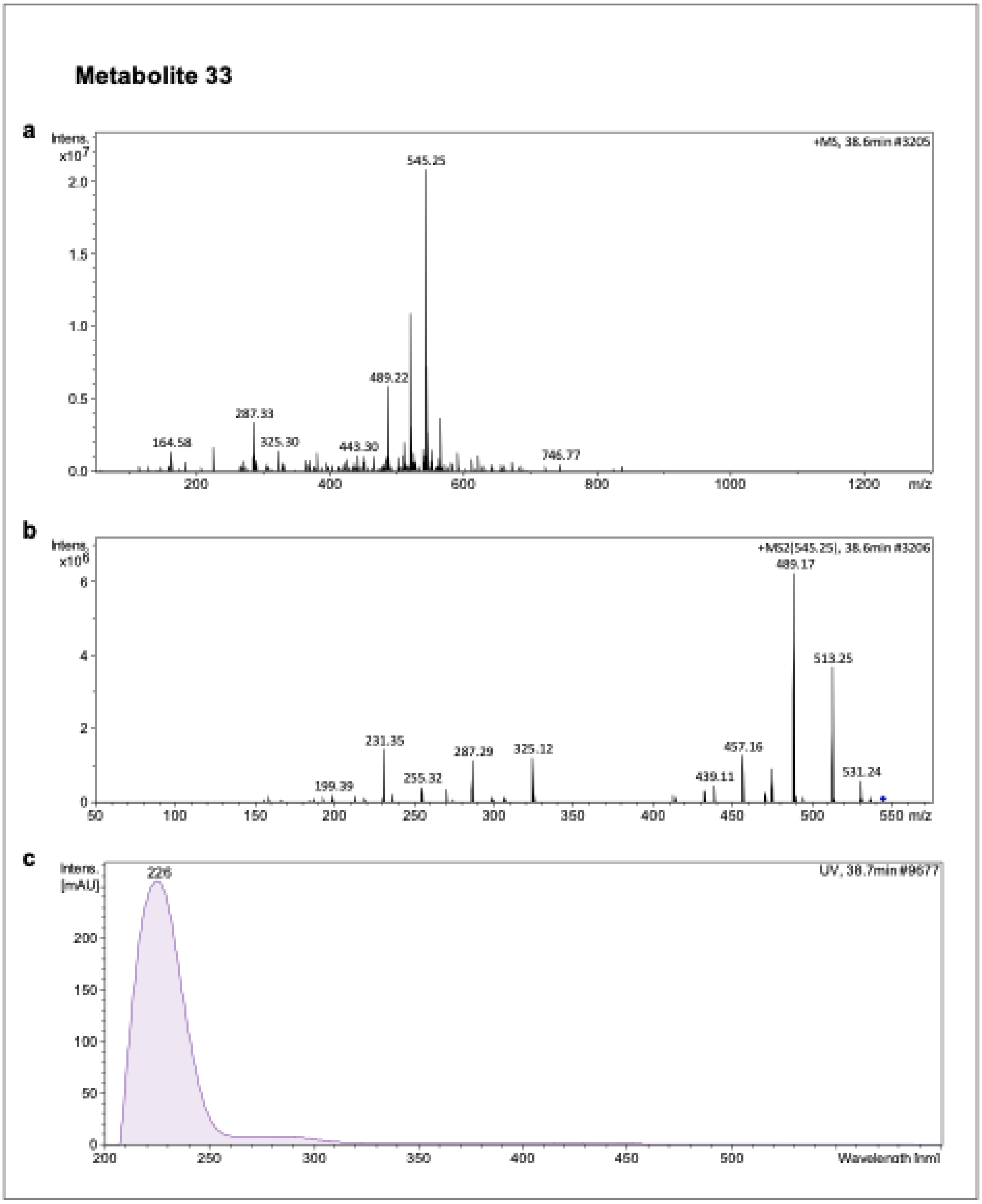

Figure S59. Spectrometric and spectroscopic data of metabolite **33**, indicated as a discriminant compound for *Piper aduncum*, including MS spectra (**a**), MS^n spectra (**b**) and UV spectra (**c**).

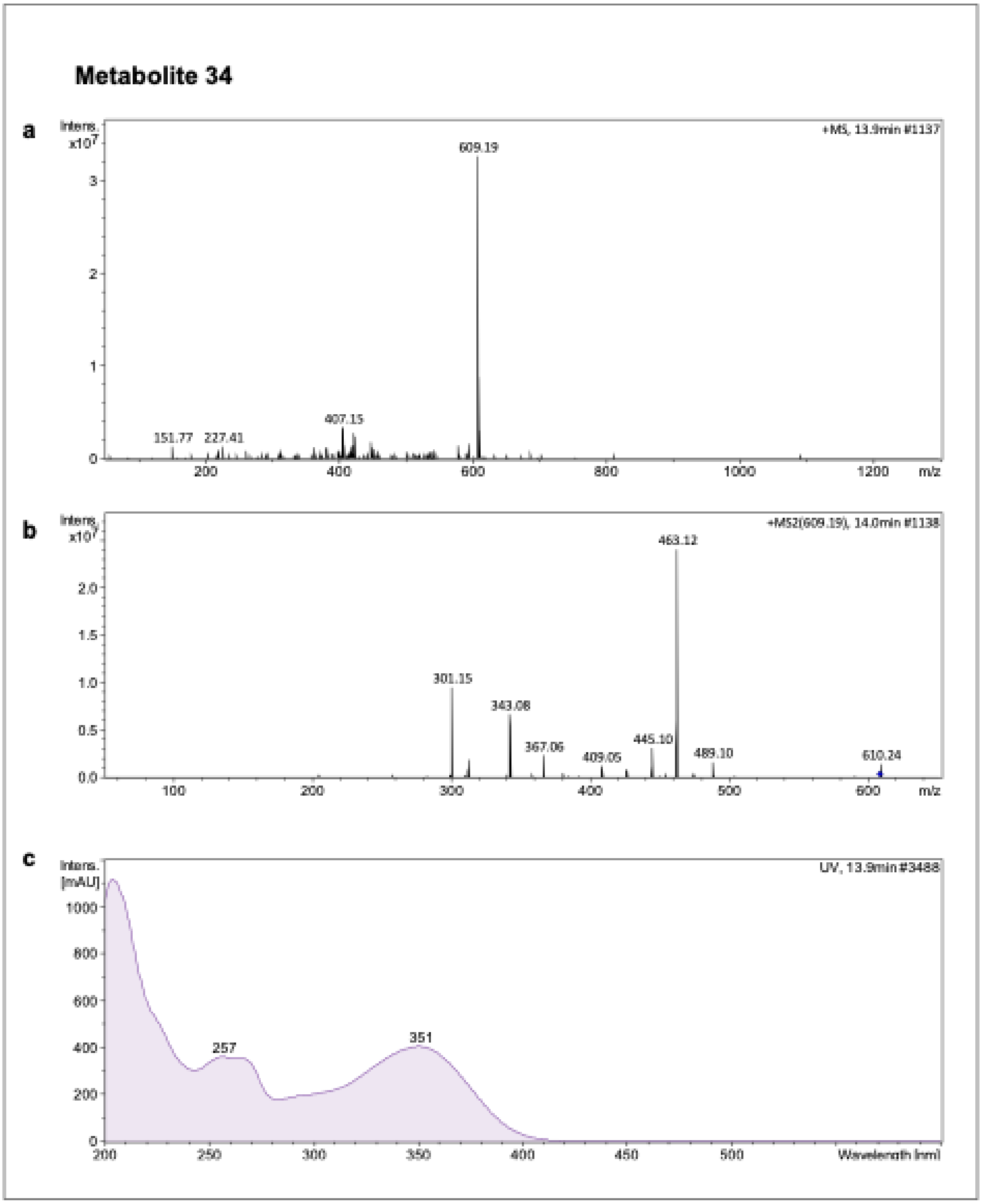

Figure S60. Spectrometric and spectroscopic data of metabolite **34**, indicated as a discriminant compound for *Piper aduncum*, including MS spectra (**a**), MS^n spectra (**b**) and UV spectra (**c**).

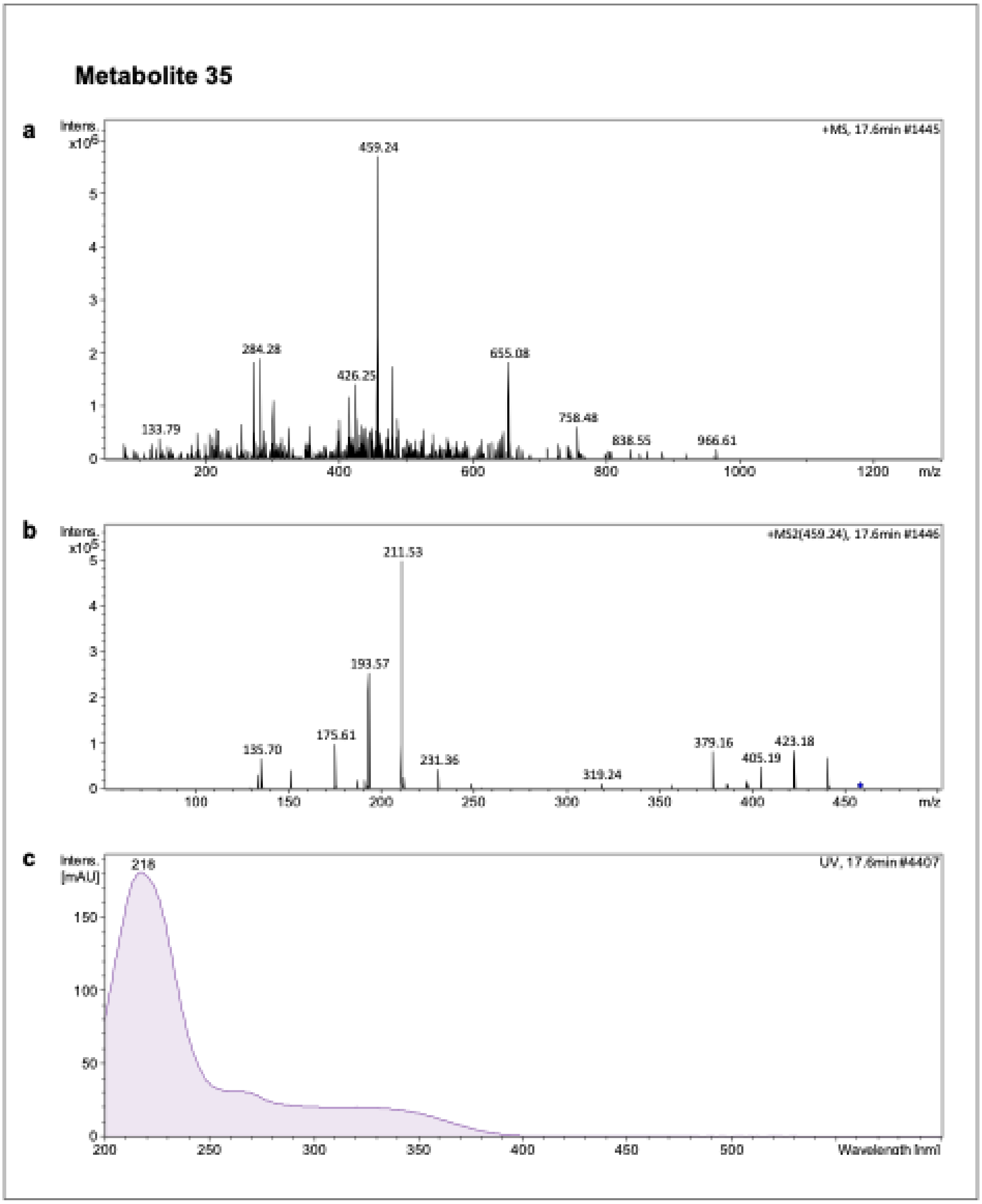

Figure S61. Spectrometric and spectroscopic data of metabolite **35**, indicated as a discriminant compound for *Piper aduncum*, including MS spectra (**a**), MS^n spectra (**b**) and UV spectra (**c**).

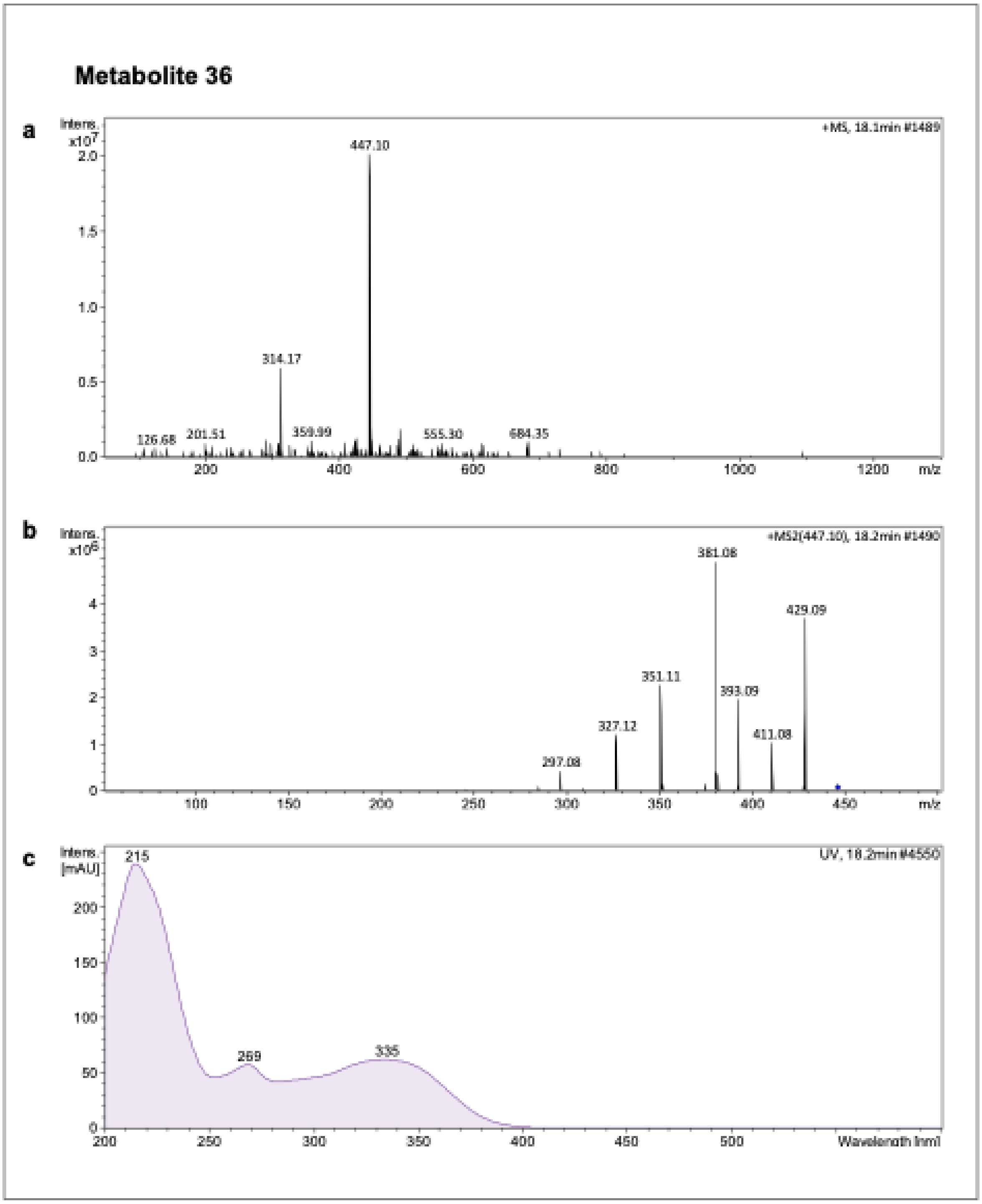

Figure S62. Spectrometric and spectroscopic data of metabolite **36**, indicated as a discriminant compound for *Piper aduncum*, including MS spectra (**a**), MS^n spectra (**b**) and UV spectra (**c**).

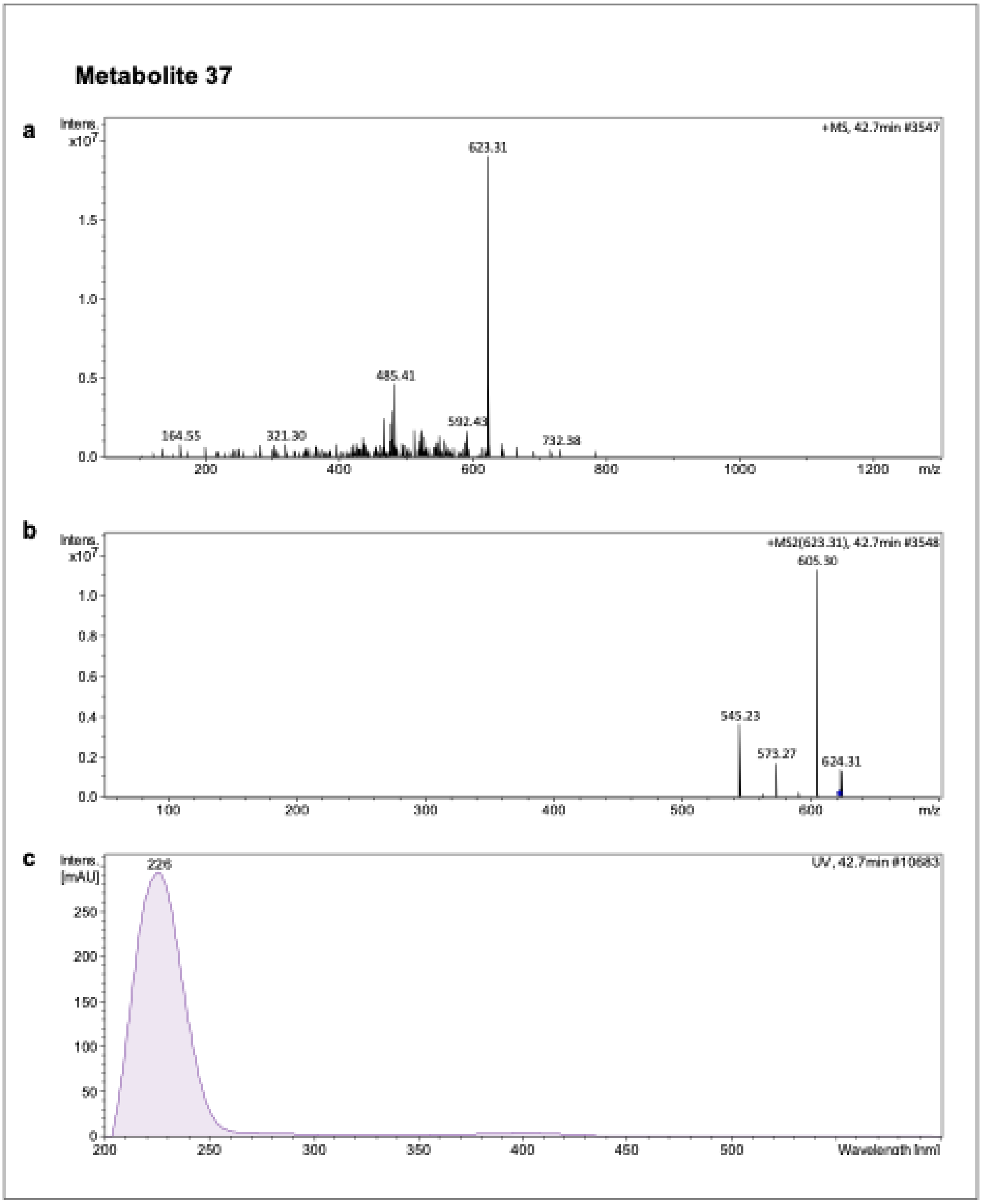

Figure S63. Spectrometric and spectroscopic data of metabolite **37**, indicated as a discriminant compound for *Piper aduncum*, including MS spectra (**a**), MS^n spectra (**b**) and UV spectra (**c**).

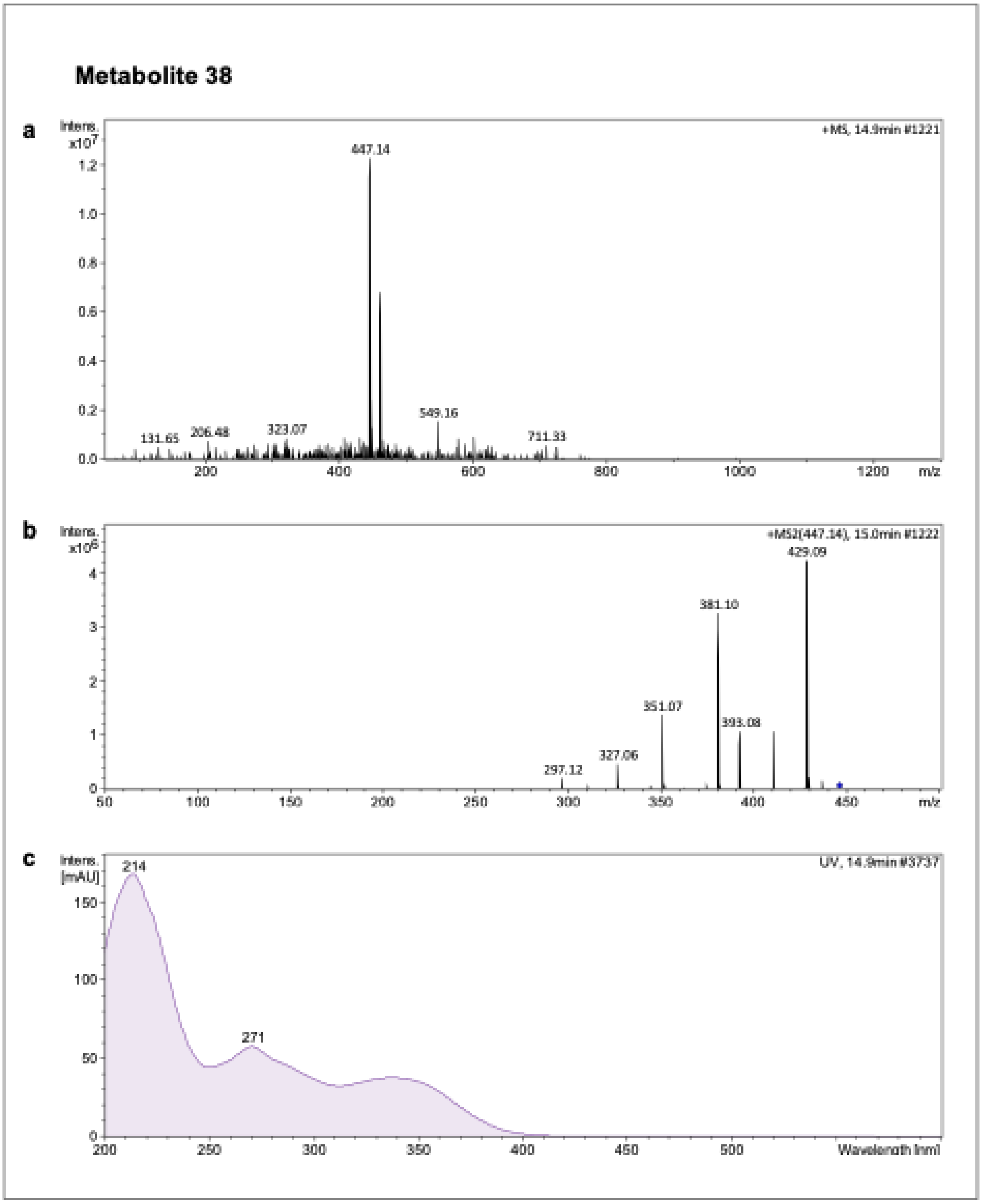

Figure S64. Spectrometric and spectroscopic data of metabolite **38**, indicated as a discriminant compound for *Piper aduncum*, including MS spectra (**a**), MS^n spectra (**b**) and UV spectra (**c**).

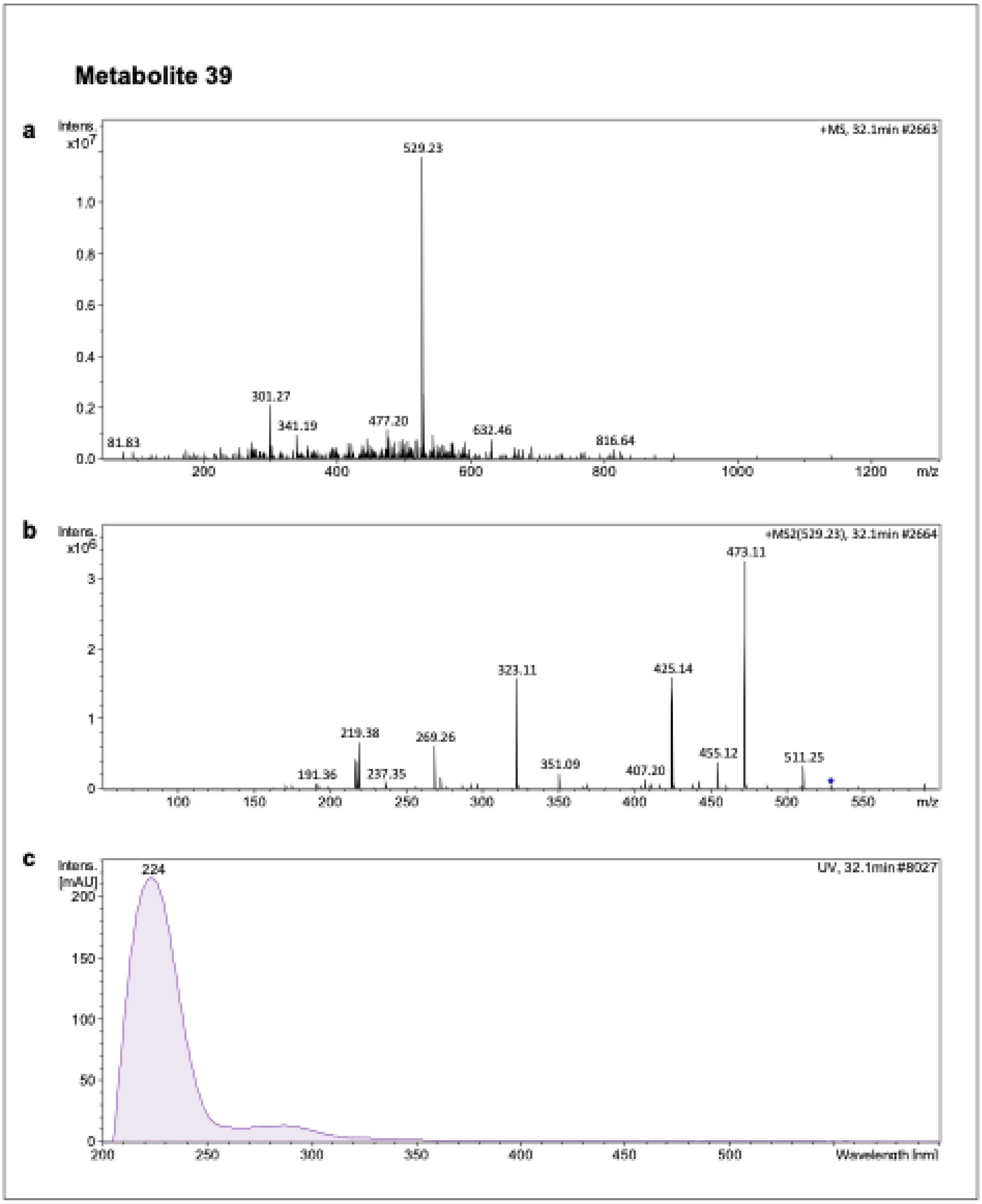

Figure S65. Spectrometric and spectroscopic data of metabolite **39**, indicated as a discriminant compound for *Piper aduncum*, including MS spectra (**a**), MS^n spectra (**b**) and UV spectra (**c**).

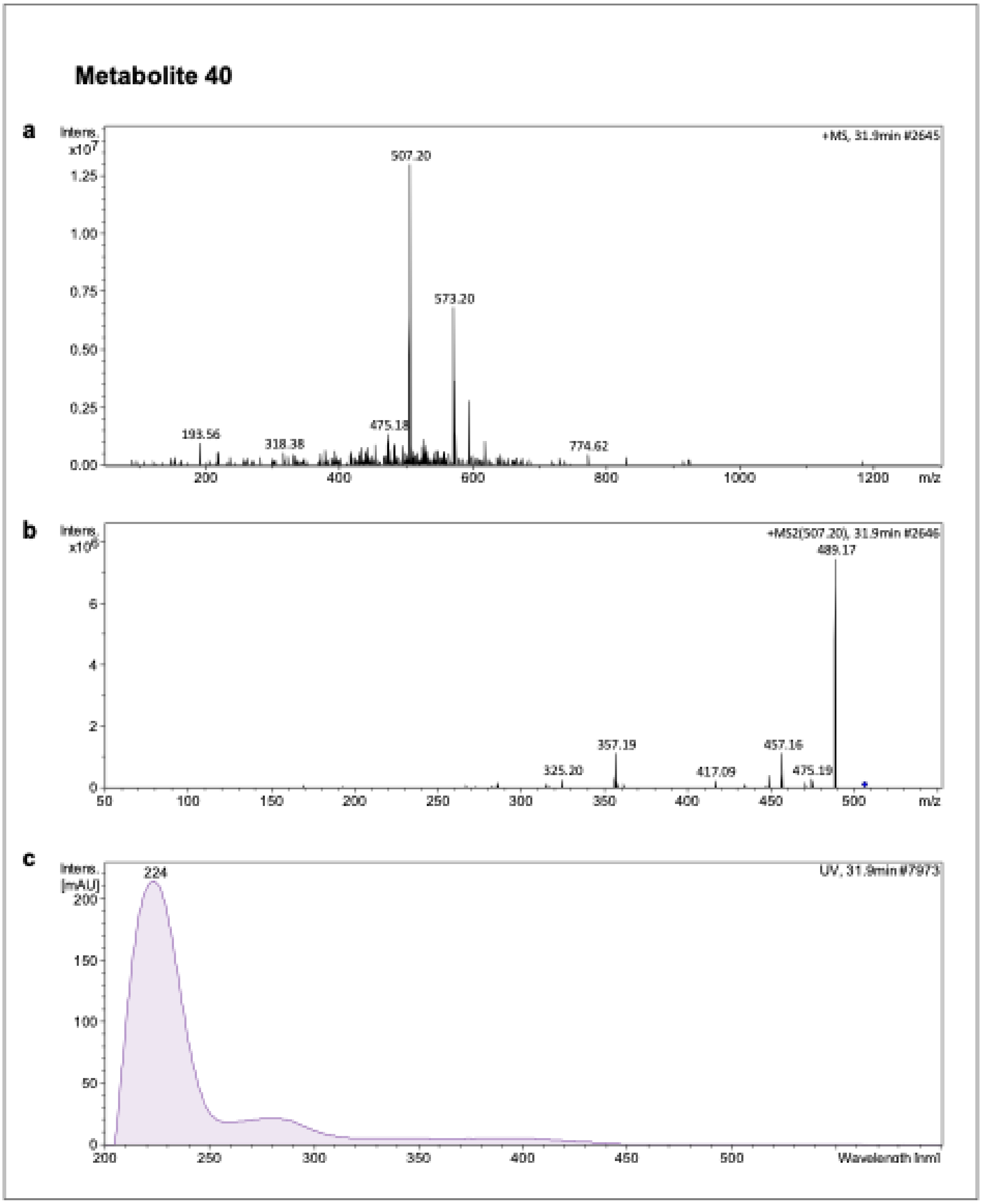

Figure S66. Spectrometric and spectroscopic data of metabolite **40**, indicated as a discriminant compound for *Piper aduncum*, including MS spectra (**a**), MS^n spectra (**b**) and UV spectra (**c**).

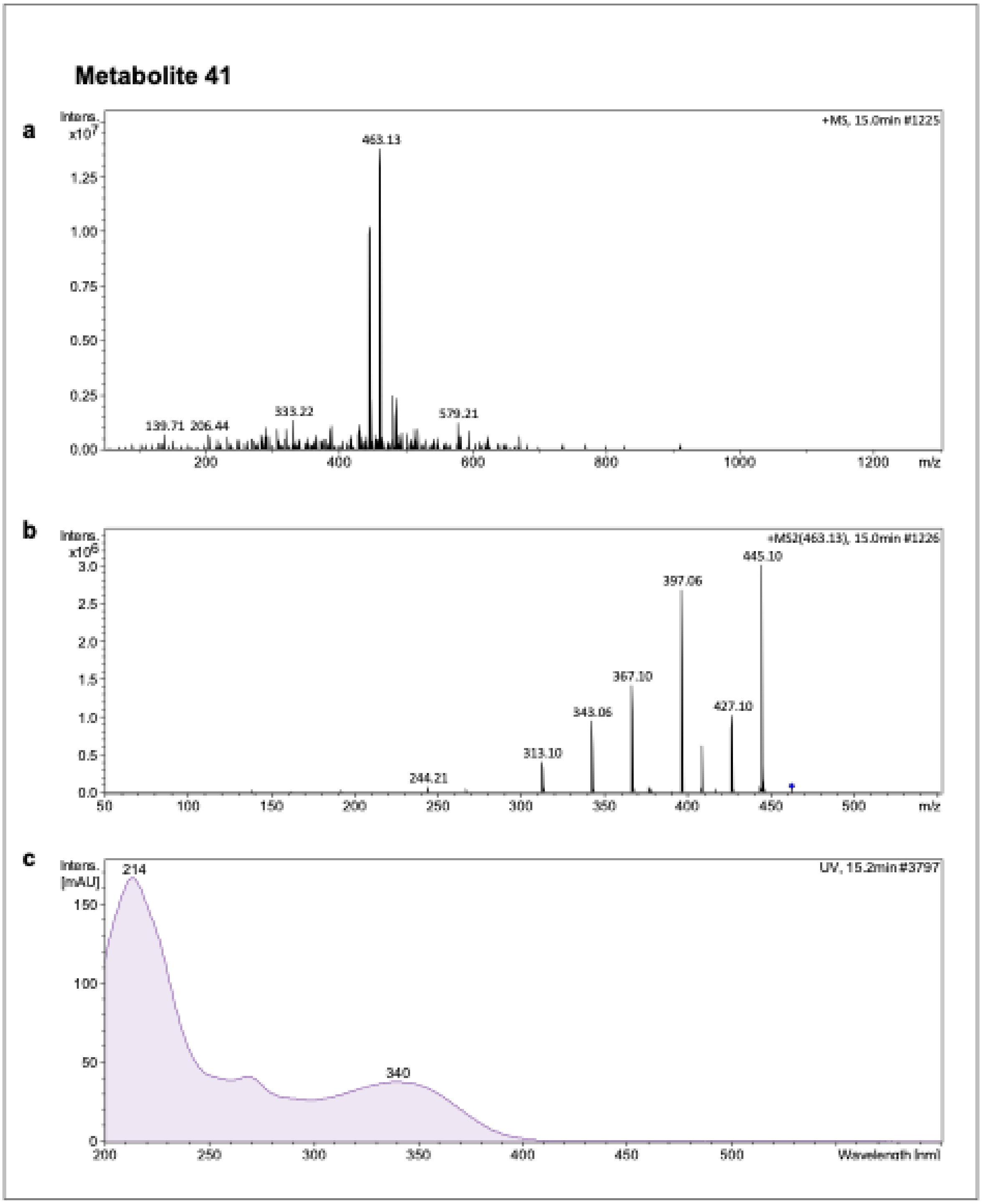

Figure S67. Spectrometric and spectroscopic data of metabolite **41**, indicated as a discriminant compound for *Piper aduncum*, including MS spectra (**a**), MS^n spectra (**b**) and UV spectra (**c**).

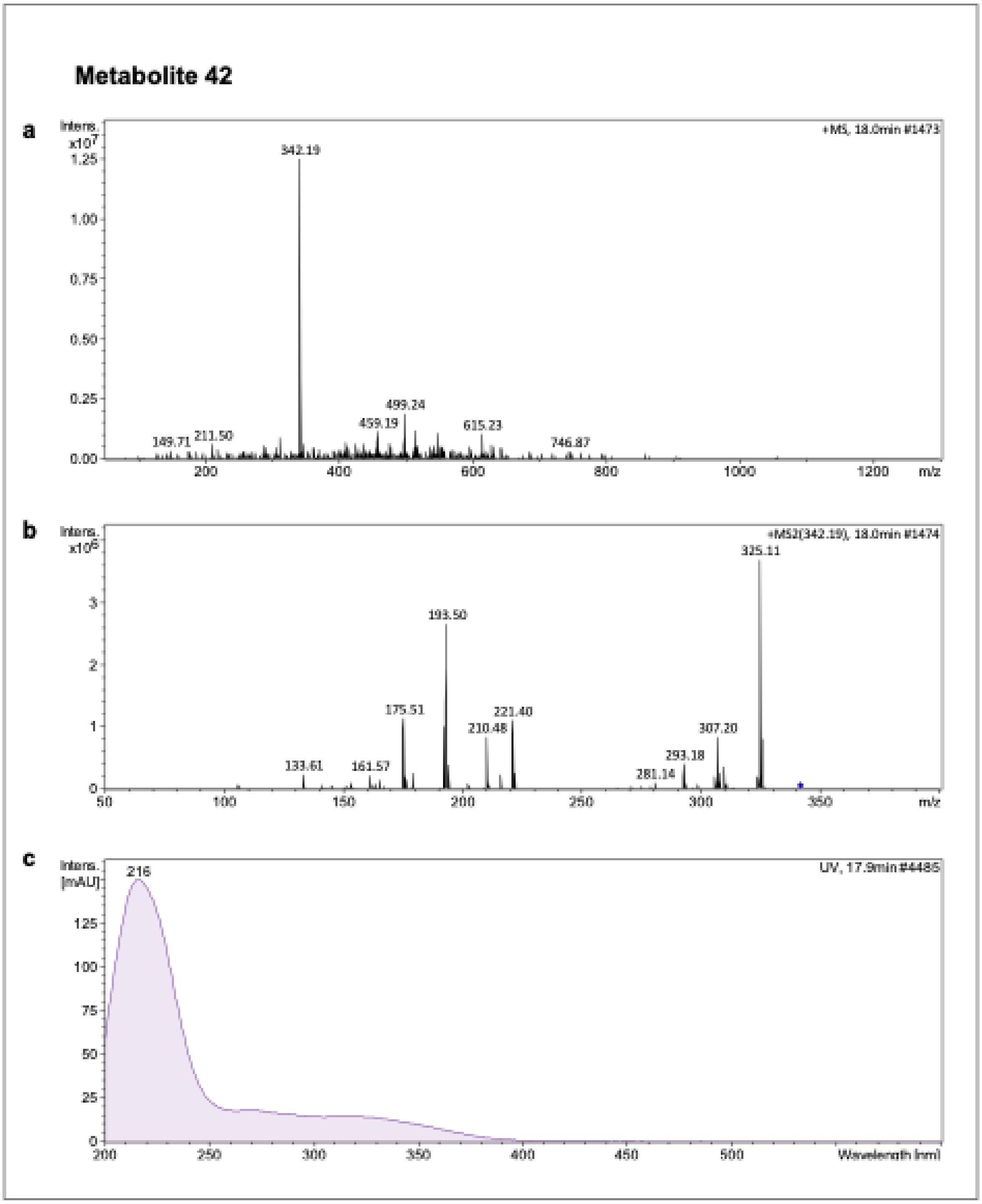

Figure S68. Spectrometric and spectroscopic data of metabolite **42**, indicated as a discriminant compound for *Piper aduncum*, including MS spectra (**a**), MS^n spectra (**b**) and UV spectra (**c**).

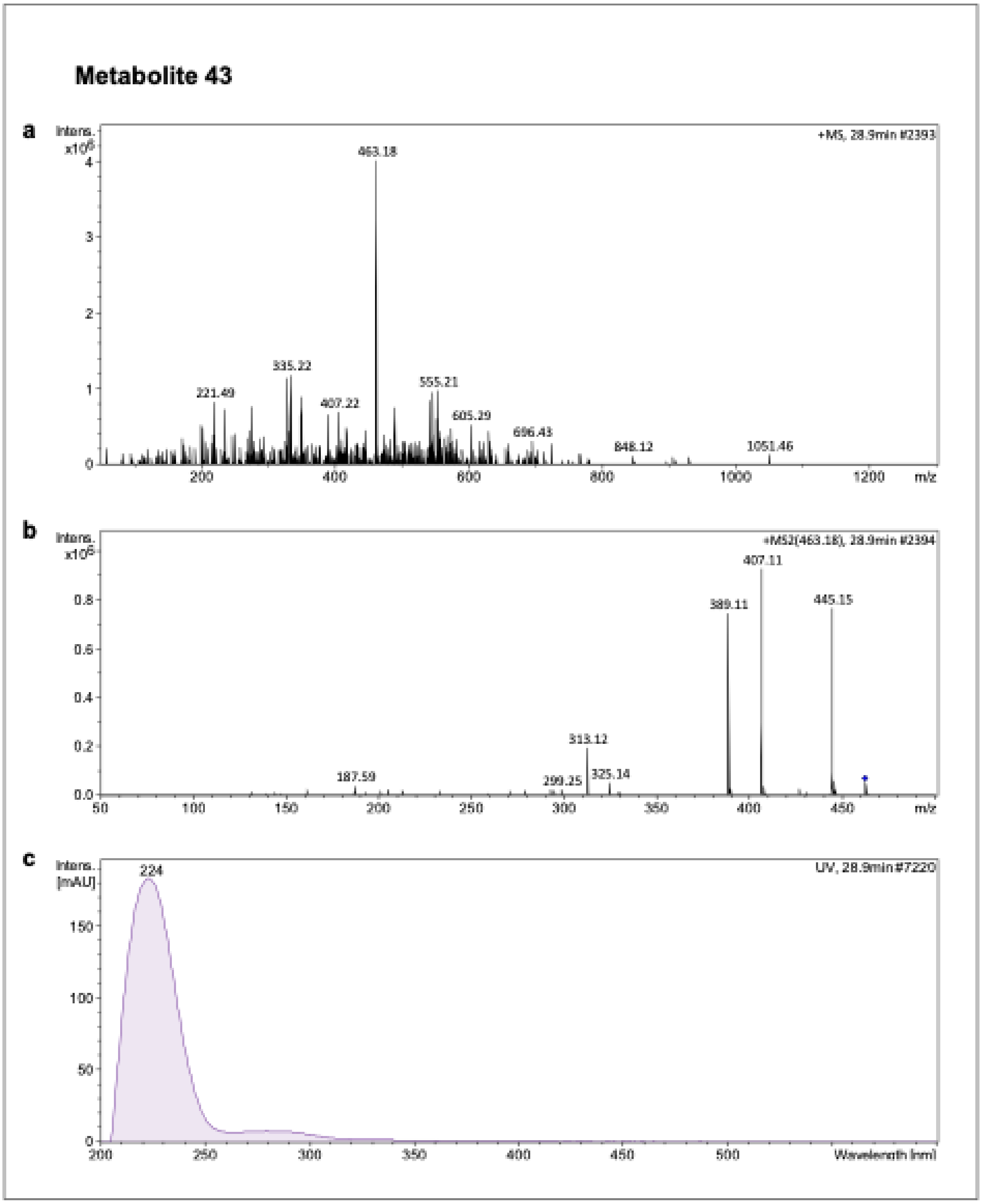

Figure S69. Spectrometric and spectroscopic data of metabolite **43**, indicated as a discriminant compound for *Piper aduncum*, including MS spectra (**a**), MS^n spectra (**b**) and UV spectra (**c**).

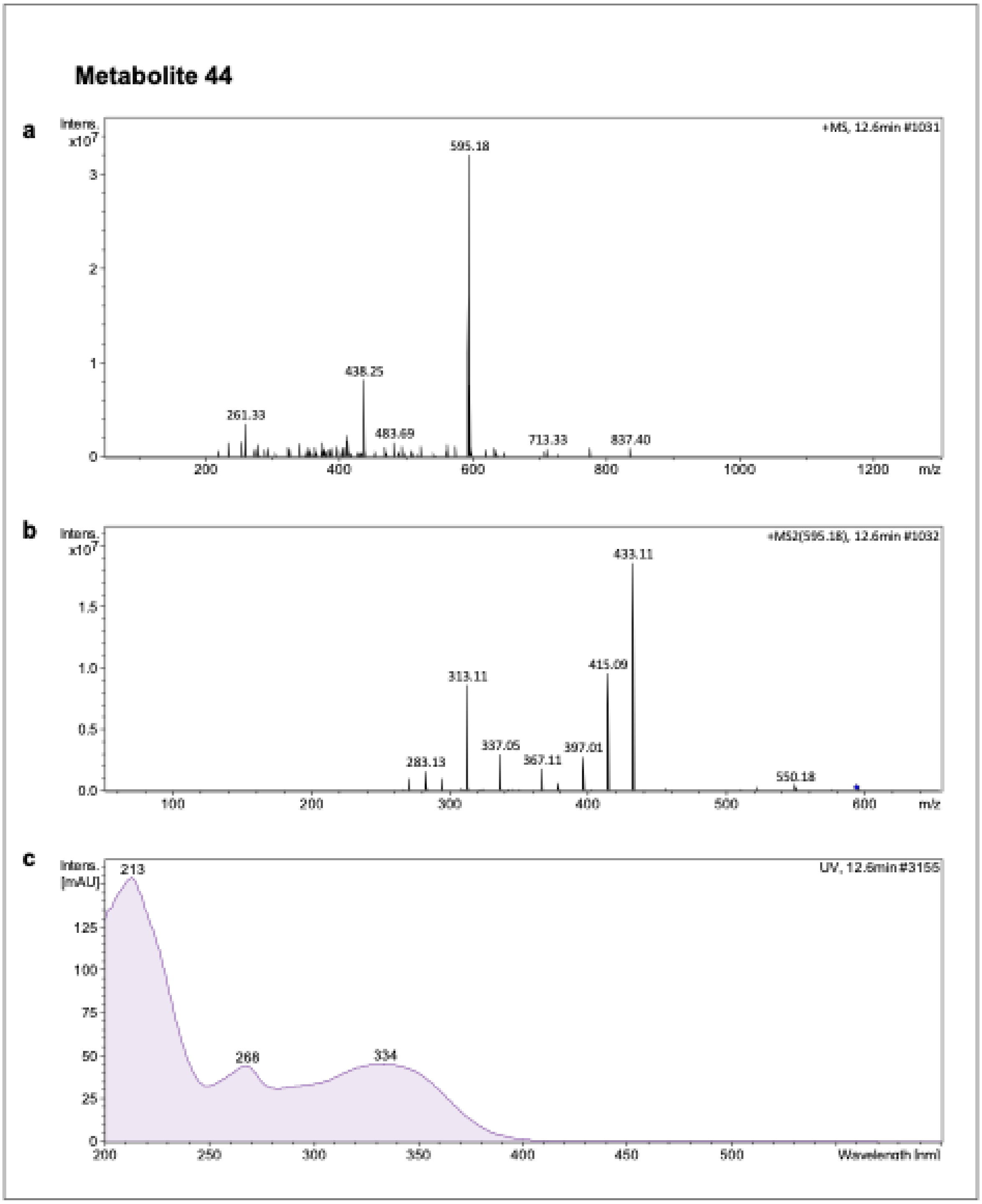

Figure S70. Spectrometric and spectroscopic data of metabolite **44**, indicated as a discriminant compound for *Piper aduncum*, including MS spectra (**a**), MS^n spectra (**b**) and UV spectra (**c**).

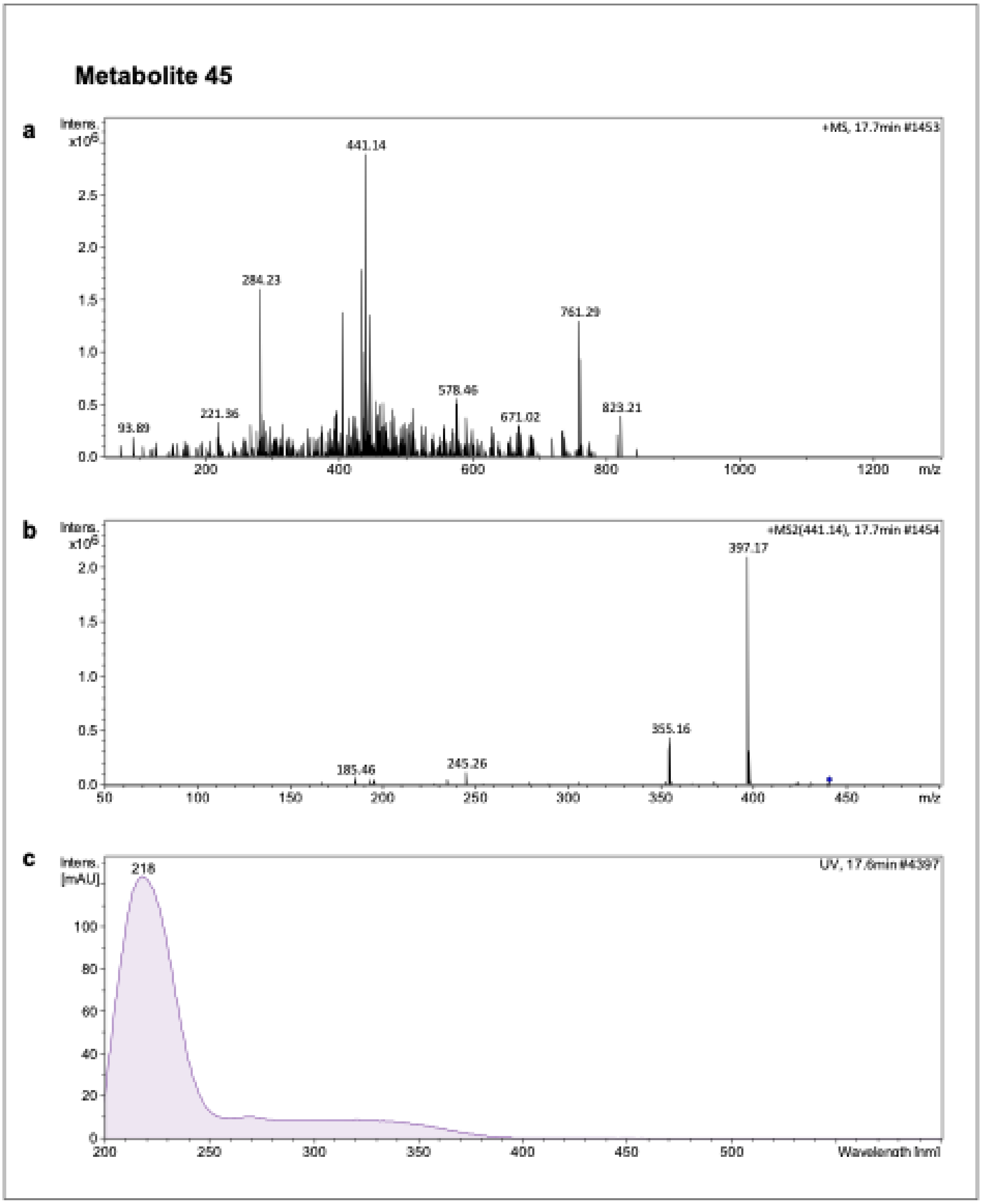

Figure S71. Spectrometric and spectroscopic data of metabolite **45**, indicated as a discriminant compound for *Piper aduncum*, including MS spectra (**a**), MS^n spectra (**b**) and UV spectra (**c**).

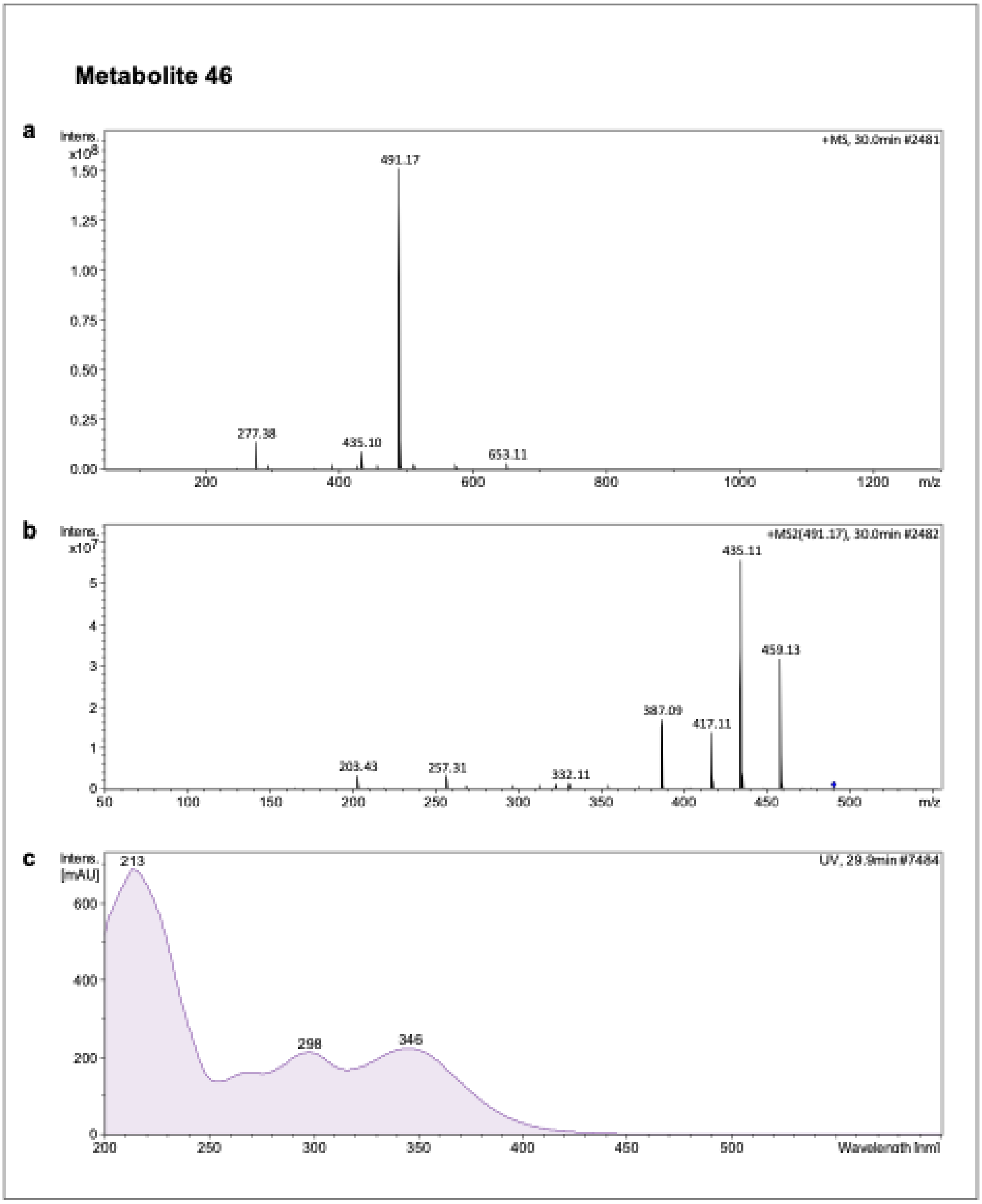

Figure S72. Spectrometric and spectroscopic data of metabolite **46**, indicated as a discriminant compound for *Piper aduncum*, including MS spectra (**a**), MS^n spectra (**b**) and UV spectra (**c**).

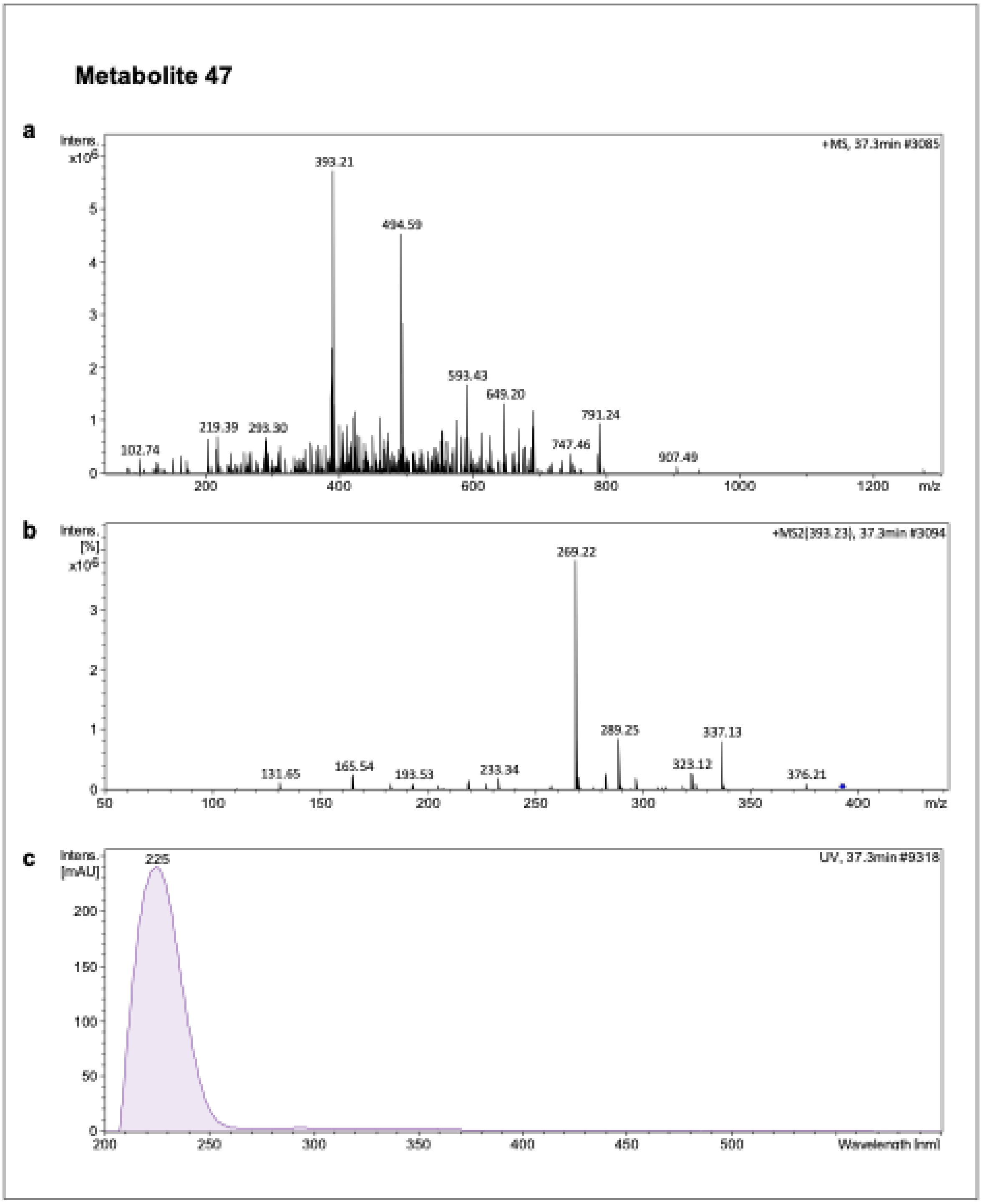

Figure S73. Spectrometric and spectroscopic data of metabolite **47**, indicated as a discriminant compound for *Piper aduncum*, including MS spectra (**a**), MS^n spectra (**b**) and UV spectra (**c**).

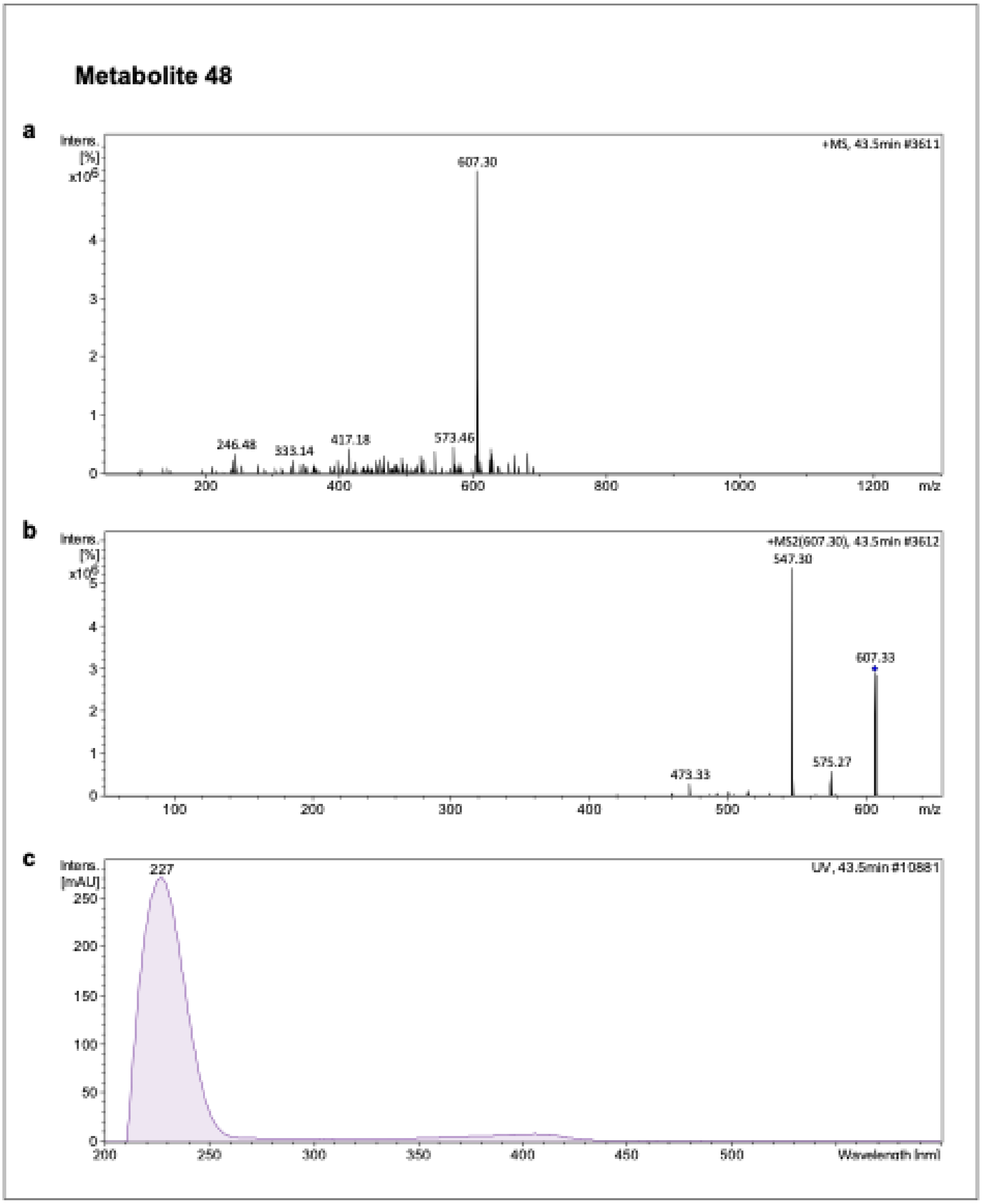

Figure S74. Spectrometric and spectroscopic data of metabolite **48**, indicated as a discriminant compound for *Piper aduncum*, including MS spectra (**a**), MS^n spectra (**b**) and UV spectra (**c**).

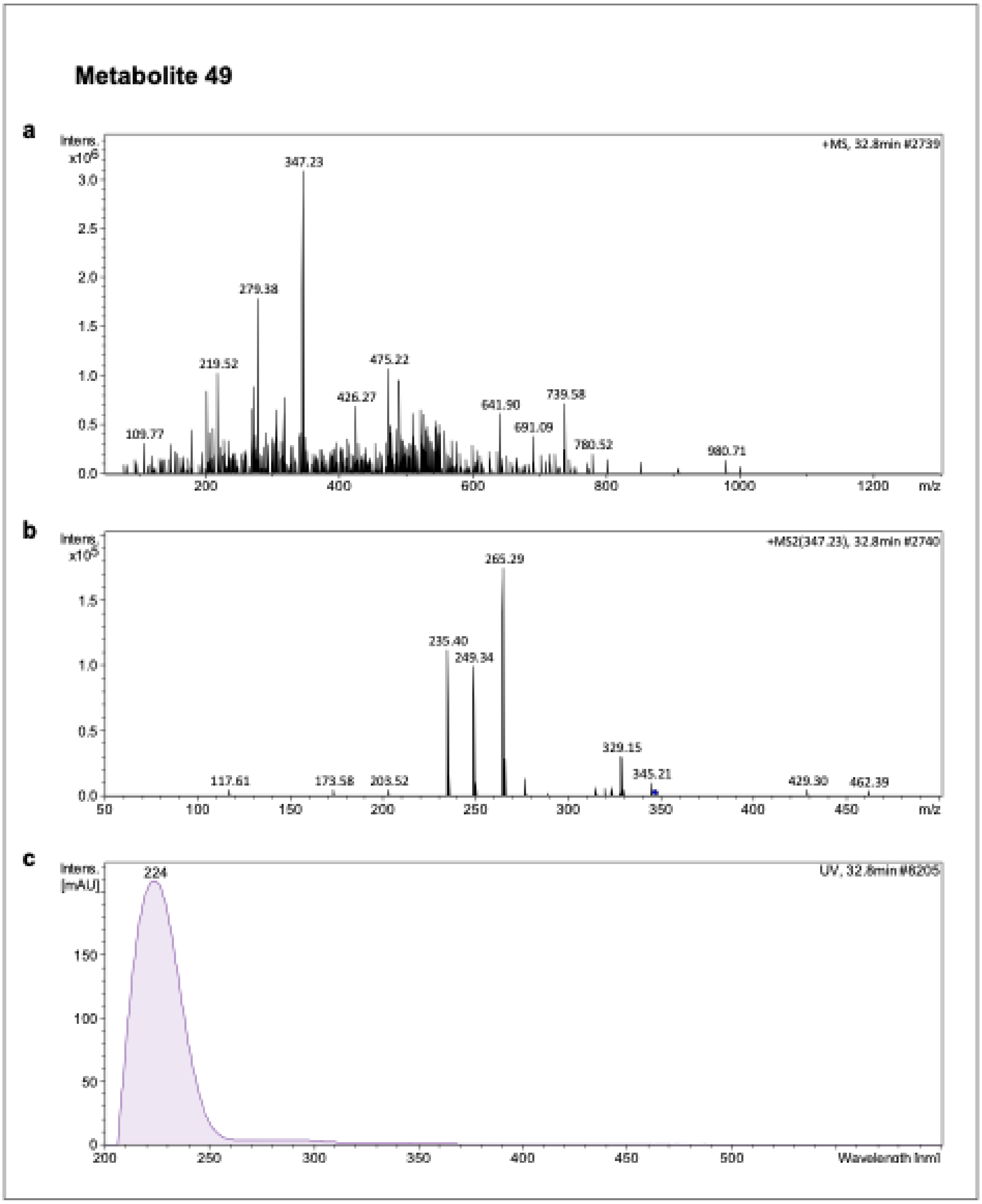

Figure S75. Spectrometric and spectroscopic data of metabolite **49**, indicated as a discriminant compound for *Piper aduncum*, including MS spectra (**a**), MS^n spectra (**b**) and UV spectra (**c**).

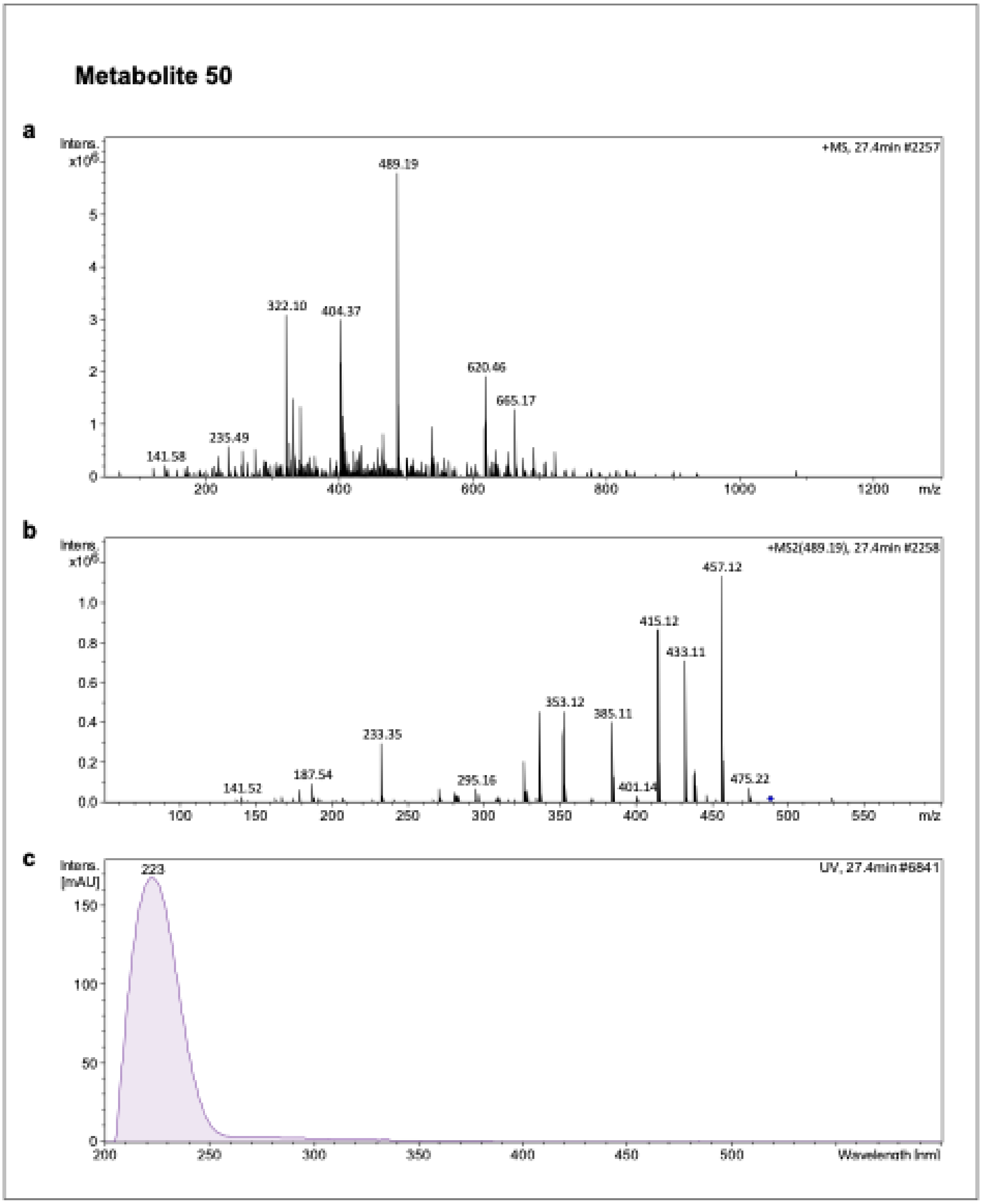

Figure S76. Spectrometric and spectroscopic data of metabolite **50**, indicated as a discriminant compound for *Piper aduncum*, including MS spectra (**a**), MS^n spectra (**b**) and UV spectra (**c**).
